# Nanobody‐Decorated Lipid Nanoparticles for Enhanced mRNA Delivery to Tumors In Vivo

**DOI:** 10.1002/adhm.202500605

**Published:** 2025-07-04

**Authors:** Pol Escudé Martinez de Castilla, Vincenzo Verdi, Willemijn de Voogt, Mariona Estapé Sentí, Arnold C. Koekman, Julian Rietveld, Sven van Kempen, Qiangbing Yang, Juliette van Merris, Guido Jenster, Martin E. van Royen, Marcel H. Fens, Sander A. A. Kooijmans, Wytske M. van Weerden, Guillaume van Niel, Pieter Vader, Raymond M. Schiffelers

**Affiliations:** ^1^ CDL Research University Medical Center Utrecht Utrecht 3584CX The Netherlands; ^2^ Institut de Psychiatrie et Neurosciences de Paris Université Paris Cité INSERM 1266 Paris 75014 France; ^3^ Laboratory of Cancer Biology and Genetics National Cancer Institute National Institutes of Health (NIH) Bethesda MD 20892 United States; ^4^ Department of Pathology University Medical Center Utrecht Utrecht 3584CX The Netherlands; ^5^ Department of Urology Erasmus MC Rotterdam 3015GD The Netherlands; ^6^ Department of Pathology Erasmus MC University Medical Center Rotterdam Rotterdam 3015GD The Netherlands; ^7^ Department of Pharmaceutics Utrecht Institute for Pharmaceutical Sciences (UIPS) Utrecht University Utrecht 3584CG The Netherlands; ^8^ Centre de Recherche en Cancérologie et Immunologie Intégrée Nantes Angers (CRCI2NA) Nantes Université Nantes 44000 France; ^9^ Department of Experimental Cardiology University Medical Center Utrecht Utrecht 3584CX The Netherlands

**Keywords:** mRNA‐lipid nanoparticles, nanobody, prostate cancer, prostate specific membrane antigen (PSMA), targeted delivery

## Abstract

Prostate cancer (PCa) ranks as the fifth leading cause of cancer‐related deaths among men worldwide. In 10–20% of the cases, PCa progresses to an incurable, castration‐resistant stage. Castration‐resistant PCa cells often overexpress prostate‐specific membrane antigen (PSMA), a membrane protein that may serve as their Achilles' heel. Over the past decades, RNA‐based therapeutics have emerged as promising treatments for a vast array of diseases, including cancer. In this study, with the ultimate goal of developing a targeted therapy for PCa, lipid nanoparticles (LNPs) are decorated with an anti‐PSMA nanobody using click chemistry with a PEG‐lipid. Direct stochastic optical reconstruction microscopy (dSTORM) and cluster analysis confirm the presence of at least one nanobody on the surface of 80% of LNPs. These anti‐PSMA LNPs exhibit enhanced and specific uptake, and mRNA transfection in PSMA+ cancer cells both in vitro and in a Zebrafish (ZF) metastatic PCa xenograft model. Additionally, in a mouse PSMA‐positive xenograft model, systemic administration results in increased LNP accumulation, but not functional mRNA delivery. These findings underscore both the potential and the challenges of using a PSMA‐targeted lipid nanoparticle system for mRNA delivery into advanced prostate cancer tumors.

## Graphical abstract

This study presents a targeted mRNA‐LNP platform engineered with anti‐PSMA nanobodies for prostate cancer therapy. Using different in vivo models, the authors demonstrate selective uptake and mRNA delivery in PSMA^+^ cancer cells. However, while targeting is enhanced, mRNA delivery efficacy is limited by tissue penetration, tumor necrosis, and immune cell uptake, underscoring key challenges in nanoparticle‐based cancer treatments.

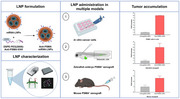

## 1 Introduction

Prostate cancer (PCa) remains a significant health burden globally, ranking as the fifth most lethal cancer among men.^[^ ^1^ ^]^ Initially, the disease typically progresses slowly and asymptomatically, characterized by the replacement of normal prostate cells with proliferating abnormal cells, eventually leading to glandular dysfunction.^[^ ^2^ ^]^ In its localized form, particularly in early stages, a range of treatment options is available, spanning from expectant management to radiotherapy and surgery.^[^ ^3^ ^]^ Central to PCa progression is the androgen receptor (AR), which regulates prostate cell growth in response to androgen stimulation.^[^ ^4^ ^]^ Androgen deprivation therapy (ADT) serves as a frontline treatment for metastatic PCa, aiming to achieve castration levels of testosterone to impede tumor growth.^[^ ^5^ ^]^

However, despite treatment advances, 10–20% of PCa cases progress to an incurable stage known as castration‐resistant prostate cancer (CRPC).^[^ ^6^ ^]^ CRPC often arises from mutations in the androgen receptor gene and the generation of splice variants, facilitating malignant PCa cell proliferation even under low androgen conditions.^[^ ^7^, ^8^ ^]^ This stage presents a significant therapeutic challenge due to its resistance to conventional treatments.

In the absence of effective therapies, RNA‐based therapeutics offer a promising approach for CRPC treatment. RNA drugs can modify the expression of any gene and, in recent decades, they have shown promise for various diseases, including cancer.^[^ ^9^ ^]^ More specifically, messenger RNA (mRNA) therapy holds great potential in cancer treatment by delivering specific genes which affect tumor growth,^[^ ^10^ ^]^ such as suicide genes or genes that flag the tumor cells to the immune system. However, one of the major challenges in using RNA therapeutics is their delivery into the target cell cytoplasm.

In particular, lipid nanoparticles (LNPs) have emerged as highly promising RNA delivery systems, offering novel strategies to enhance the delivery and efficacy of these next‐generation therapies.^[^ ^11^ ^]^ Moreover, LNPs are characterized as versatile drug delivery systems (DDS) due to their modularity, biocompatibility, and capacity to encapsulate an extensive range of therapeutic payloads, spanning from small molecules to nucleic acids, including siRNA and mRNA.^[^ ^12^, ^13^, ^14^, ^15^, ^16^, ^17^ ^]^ Lipid nanoparticles have demonstrated remarkable efficacy in treating liver diseases and genetic disorders by RNA interference (RNAi), as well as in developing effective mRNA vaccines for infectious diseases such as COVID‐19.^[^ ^16^ ^]^

Despite their therapeutic potential, mRNA‐LNPs’ clinical utility in cancer is hindered by limited accumulation at the tumor site, primarily due to predominant accumulation in the liver.^[^ ^18^, ^19^ ^]^ In this context, active targeting strategies present a promising opportunity to address this challenge. Active targeting can be achieved by decorating the surface of LNPs with structures that recognize surface epitopes on the desired target cell. The identification of heavy chain‐only antibodies in camelids prompted investigation into single‐domain antibodies, referred to as nanobodies (VHHs), as targeting agents. Compared to antibodies and antibody variable fragments, VHHs are smaller (≈15–20 kDa), exhibit low immunogenicity, and are more resistant to changes in temperature and pH.^[^ ^20^ ^]^ Coupling VHHs to the surface of a nanoparticle can improve its cellular uptake and therefore lead to enhanced functional cargo delivery in specific subsets of cells expressing the targeted antigen.^[^ ^21^, ^22^, ^23^ ^]^ Therefore, VHHs are promising ligands for enhancing the targeting capacity of LNPs.

Remarkably, 90% of CRPCs display elevated expression of prostate‐specific membrane antigen (PSMA),^[^ ^24^ ^]^ a plasma membrane glycoprotein with low levels of expression in normal tissues. PSMA‐targeted imaging and therapy are current clinical practices in prostate cancer management utilizing radionuclide‐conjugated small PSMA‐binding tracers.^[^ ^25^ ^]^ This highlights PSMA's potential as a target for novel therapeutic strategies aimed at addressing advanced stages of PCa. Indeed, the integration of PSMA‐targeting ligands onto different types of nanoparticles has previously been shown to enhance uptake and cargo delivery in PSMA^+^ tumor models in vivo.^[^ ^26^, ^27^, ^28^, ^29^ ^]^ However, there are few, if any, published studies on the use of a PSMA‐nanobody as a targeting ligand for LNPs.

In this study, with the ultimate aim of developing a targeted therapy against PCa, we employed click chemistry and post‐insertion to generate mRNA‐LNPs decorated with a PSMA‐binding nanobody.^[^ ^30^ ^]^ We then characterized our particles through dSTORM imaging to verify that VHH was present on the surface of the LNPs. We investigated the uptake/biodistribution and functional mRNA transfection of these anti‐PSMA LNPs in vitro in cancer cell lines and in zebrafish and mouse xenograft models. By combining the advantages of live imaging in zebrafish with the translational relevance of murine models, we aimed to elucidate the potential of anti‐PSMA LNPs as a viable strategy for targeted cancer therapy.

## 2 Results

### 2.1 Formulating and Characterizing anti‐PSMA LNPs

**Figure 1:**
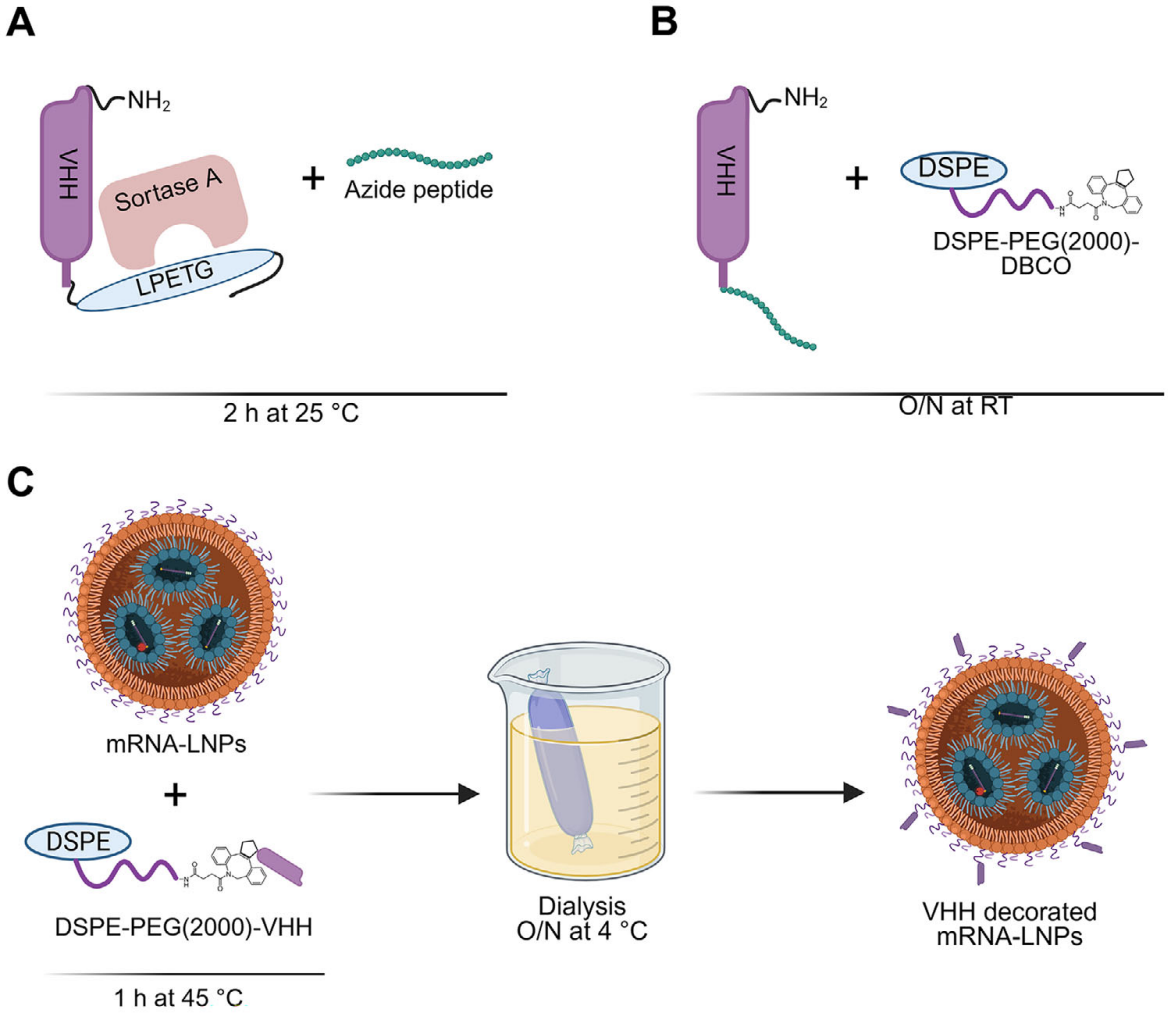
Schematic overview illustrating click chemistry and the post‐insertion of VHHs onto the surface of LNPs. A) Sortase A cuts LPETG (sortase A tag) to allow coupling of an azide peptide to the C‐terminal of VHH. B) Click chemistry reaction between Azide‐VHH and DBCO‐PEG‐lipid. C) Post‐insertion of DSPE‐PEG‐VHH onto the surface of LNPs followed by dialysis overnight to obtain VHH‐decorated mRNA‐LNPs.

The overall aim of our research was to enhance the specificity and efficiency of LNPs for targeted cancer mRNA delivery. Our approach focused on decorating the surface of LNPs with PSMA‐targeting nanobodies through post‐insertion of the VHH clicked to a PEG‐lipid. Click chemistry was preferred to other coupling methods that rely on copper‐catalysis or random maleimide‐thiol coupling, because of its high biocompatibility, specificity and efficiency.^[^ ^31^ ^]^ Through the click chemistry approach we employed, azide residues can be added to the C‐terminus of any targeting ligand through Sortase A‐mediated transpeptidation.^[^ ^32^ ^]^ For azide introduction to VHHs, it is essential for VHHs to have an LPETG sequence as the recognition of this sequence by Sortase A leads to the replacement of glycine with Gly3‐azide, resulting in the formation of VHH‐azide (**Figure** **1**A). Following this, azide residues react with DBCO‐conjugated‐PEG‐lipids through a click chemistry reaction (Figure 1B), and the resulting DSPE‐PEG‐VHHs can then be post‐inserted onto the surface of LNPs (Figure 1C).

We first optimized the click chemistry reaction and post‐insertion by finding the optimal Sortase A concentration (1 or 5 µm) and VHH:DSPE‐PEG(2000) ratio (1:1, 1:3 or 1:10) using gel electrophoresis analysis. To this end, samples were obtained from various stages of VHH click chemistry to a PEG‐lipid (Figure S1A, Supporting Information) and from post‐inserted LNPs (Figure S1B, Supporting Information). Subsequently, the samples were subjected to gel electrophoresis analysis, and the gels were stained for total protein content. The results of the analyzed gel in Figure S1A (Supporting Information) showed that 1 µm concentration of Sortase A was preferable to 5 µm, as this condition exhibited the least amount of undesired subproducts (band at 16 kDa). At the same time, the 1:3 ratio of VHH to DSPE‐PEG(2000) exhibited reduced VHH multimerization and allowed a higher conjugation of the VHH to the PEG‐lipid as seen by the band appearing at ≈25 kDa (Figure S1A, Supporting Information). We concluded then that the condition resulting in the optimal conjugation of VHH to DSPE‐PEG(2000) with minimal undesired products was 1 µm concentration of Sortase A and a 1:3 ratio of VHH to DSPE‐PEG(2000) (Figure S1A, Supporting Information). From this point onward, we decided to produce our targeted‐LNPs using these conditions.

Furthermore, the targeted‐LNPs final solution was passed through a Vivaspin 100 kDa filter. Using dot blotting with an anti‐Myc antibody, we detected the presence of VHH on LNPs (identified by Cy5‐mRNA), and no leakage of the bound VHH was observed in the flowthrough (Figure S1C, Supporting Information).

### 2.2 dSTORM Imaging of Anti‐PSMA LNPs Confirmed at Least One VHH on the Surface of 80% of the Particles

**Figure 2:**
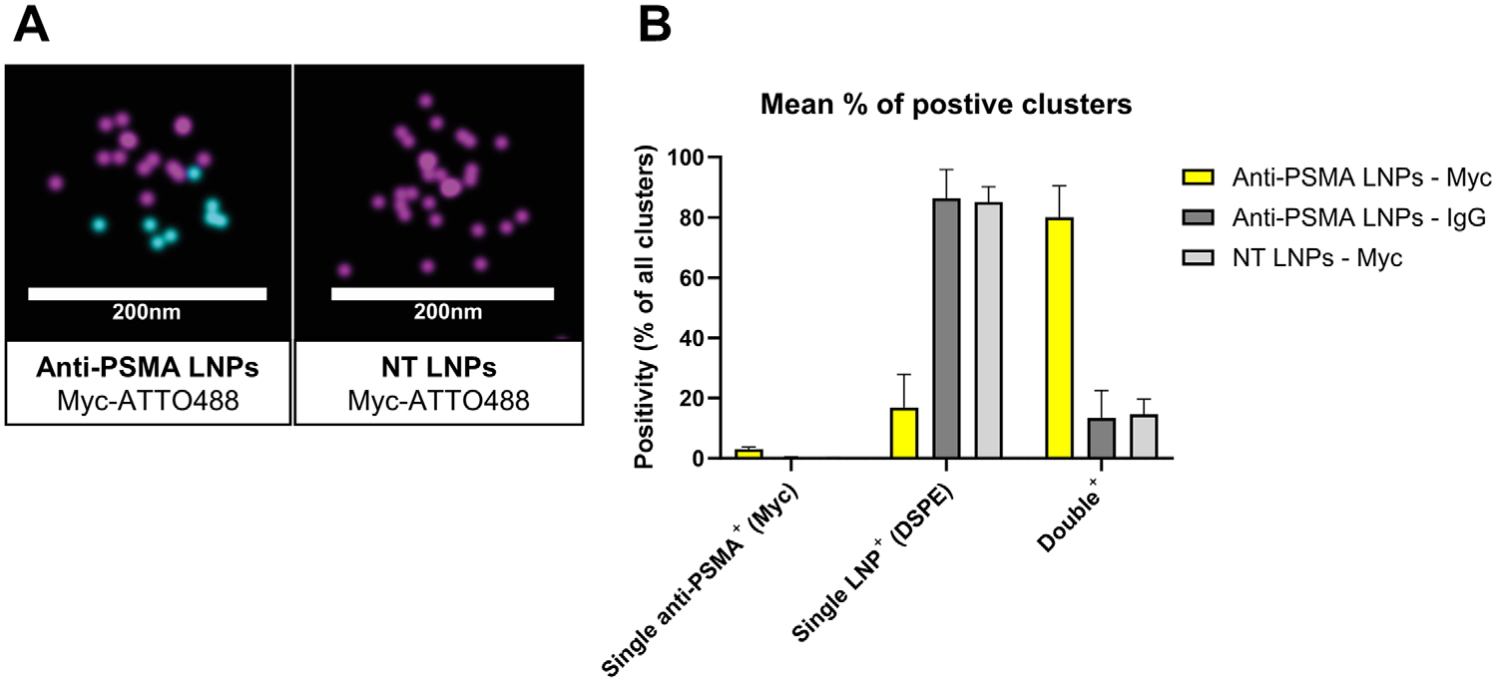
Through dSTORM imaging and cluster analysis we identified VHH on the surface of anti‐PSMA LNPs. LNPs were labeled with Cy5‐DSPE (pink), and VHH was detected after incubation with 10 µg mL^−1^ anti‐Myc‐Atto 488 antibody (cyan). A) ONI Super‐resolution microscopy image of a frame with clusters for anti‐PSMA LNPs (left) and nontargeted LNPs (NT LNPs, right). B) Through clustering analysis, we determined that ≈80% of anti‐PSMA LNPs contained at least one VHH on their surface. TIRF acquisition was performed with a Nanoimager‐S (ONI) and CODI online analysis tool was used for the filtering of localizations and HDBSCAN cluster analysis. Data represent mean ± SD (*n* = 2 technical replicates).

We formulated DSPE‐Cy5 labeled LNPs (**Figure** **2**A, pink) and post‐inserted them with anti‐PSMA VHH as previously described. For this experiment, the molar percentage of VHH‐PEG‐lipid added to the LNPs was 0.2%, in relation to the total lipid concentration of the LNPs. We incubated the anti‐PSMA LNPs with an Anti‐Myc‐Atto 488 antibody (Figure 2A, cyan), which recognizes the Myc‐tag in the VHH, or with mouse IgG‐Atto 488 control. We then performed dSTORM imaging to confirm VHH inclusion onto the surface of anti‐PSMA LNPs (Figure 2A, left) and VHH absence in DSPE‐PEG(2000) post‐inserted LNPs (nontargeted LNPs) (Figure 2A, right), using anti‐Myc antibody and mouse IgG control antibodies. Through clustering analysis of double positive clusters (LNP & VHH), we determined that ≈80% of anti‐PSMA LNPs contained at least one VHH on their surface (Figure 2B).

### 2.3 Enhanced Receptor‐Mediated LNP Uptake and mRNA Transfection in PSMA^±^ Cells Treated with Anti‐PSMA LNPs In Vitro

With the ultimate goal to develop a targeted therapy, we developed anti‐PSMA decorated LNPs encapsulating Cy5‐labeled EGFP mRNA for specific and enhanced transfection in PSMA^+^ cancer cells. Throughout this study, mRNA‐LNPs with different post‐inserted DSPE‐PEG‐VHH percentages and ratios were synthesized and characterized by size, polydispersity index, zeta potential, and mRNA encapsulation efficiency. An overview of their characteristics can be found in Table S1 (Supporting Information).

**Figure 3:**
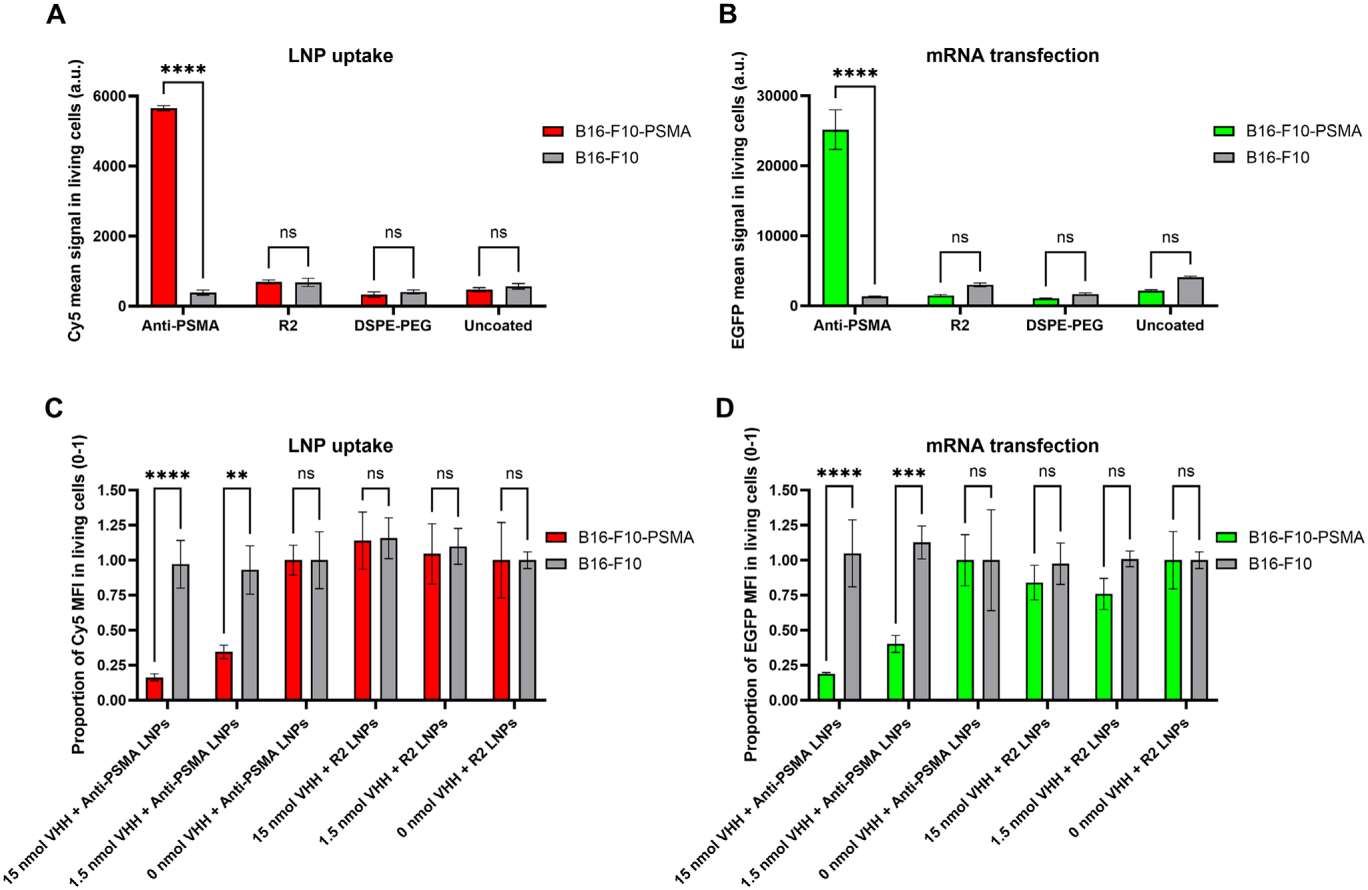
LNP uptake and mRNA transfection of anti‐PSMA LNPs in PSMA^+^ cells are receptor‐mediated. A,B) Flow cytometry analysis for uptake (A) and mRNA transfection (B) 24 h after addition of 200 ng of targeted‐LNPs (0.2% DSPE‐PEG(2000)‐VHH post‐insertion) encapsulating Cy5‐labeled EGFP mRNA in B16‐F10‐PSMA & B16‐F10 cell lines. In parallel, B16‐F10‐PSMA & B16‐F10 cells were subjected to 5‐min incubations with 15, 1.5, or 0 nmols of unconjugated anti‐PSMA VHH. After the VHH incubation, we added 200 ng of anti‐PSMA or R2‐LNPs (0.2% DSPE‐PEG(2000)‐VHH post‐insertion) encapsulating Cy5‐labeled EGFP mRNA to each well. 24 h after LNP addition, we measured the LNP uptake and mRNA transfection results by flow cytometry. C,D) Results are shown as a proportion of the LNP uptake (C) and mRNA transfection (D) for each of the LNP treatments in respect to their paired conditions without pre‐treatment of unconjugated anti‐PSMA‐VHH. A Two‐Way ANOVA with Šídák's correction for multiple comparisons test was performed comparing the mean signals of each condition in between the different cell lines. ****, *p*‐value < 0.0001; ***, *p*‐value < 0.001; **, *p*‐value < 0.01; *, *p*‐value < 0.05; ns: no significant difference. Data represent mean ± SD (*n* = 3 wells) with at least 5000 cells per well.

We started by validating the PSMA expression of the cell lines to be used with a PSMA‐antibody through flow cytometry (Figure S2, Supporting Information). We subsequently evaluated our anti‐PSMA LNPs (0.2% VHH‐DSPE‐PEG post‐insertion) in vitro using two mouse melanoma cell lines that were identical except for their PSMA expression: B16‐F10 cells (PSMA^−^) and B16‐F10‐PSMA cells (PSMA^+^). Anti‐PSMA LNPs exhibited a 14x increase in uptake (**Figure** **3**A) and a 18x increase in functional mRNA delivery (Figure 3B) in B16‐F10‐PSMA cells compared to B16‐F10 cells (Figure 3A,B). In order to compare our targeted‐LNPs to untargeted LNPs, we decided to include DSPE‐PEG LNPs, uncoated LNPs and VHH‐R2‐DSPE‐PEG post‐inserted LNPs (R2‐LNPs) as controls. DSPE‐PEG LNPs were used as a control for the effect of post‐insertion of 0.2% DSPE‐PEG(2000) and R2‐LNPs were used as a negative control as VHH‐R2 is not expected to specifically bind any cells or tissues. R2‐LNPs along with DSPE‐PEG and uncoated LNPs showed much lower LNP uptake (Figure 3A) and mRNA transfection (Figure 3B) compared to anti‐PSMA LNPs in B16‐F10‐PSMA cells. Furthermore for R2, DSPE‐PEG and uncoated LNPs, differences in uptake (Figure 3A) and transfection (Figure 3B) were nonsignificant between B16‐F10 and B16‐F10‐PSMA. There were no major differences in uptake and transfection between R2, DSPE‐PEG and uncoated LNPs at this % of post‐inserted DSPE‐PEG (0.2%). Moreover, we also tested our targeted LNPs in two human PCa cell lines with different PSMA expression patterns; namely DU145 (PSMA^−^) and LNCaP (PSMA^+^). Similarly, anti‐PSMA LNPs exhibited 22x increase in uptake and a 29x increase in mRNA transfection when compared to R2‐LNPs in LNCaP cells (Figure S3, Supporting Information), whereas the uptake and mRNA transfection of the different LNPs led to nonsignificant differences in DU145.

We also wanted to elucidate what would be the best molar% of post‐inserted DSPE‐PEG(2000)‐VHH in order to maximize the uptake and transfection of our targeted LNPs. We decided to post‐insert 1 and 0.2% of DSPE‐PEG(2000)‐VHH onto the surface of our LNPs, and we tested them in vitro in B16‐F10 and B16‐F10‐PSMA cells (Figure S4, Supporting Information). From those experiments, we inferred that 0.2% post‐inserted anti‐PSMA was more favorable in terms of enhanced uptake (Figure S4A, Supporting Information) and transfection (Figure S4B, Supporting Information) in B16‐F10‐PSMA cells, with still low signals in B16‐F10 (PSMA^−^) cells. At the same time, we could validate that the percentage DSPE‐PEG(2000) inversely correlates with mRNA transfection of the particles; almost completely abolishing it with 1% of post‐inserted DSPE‐PEG(2000) (Figure S4B, Supporting Information).

To prove that anti‐PSMA LNPs uptake and mRNA transfection are PSMA‐receptor mediated, we conducted a transfection assay pre‐treating B16‐F10 and B16‐F10‐PSMA cells with different amounts of free unconjugated anti‐PSMA‐VHH (Figure 3C,D). A significant decrease in uptake (Figure 3C) and functional transfection (Figure 3D) was observed in anti‐PSMA LNP‐treated B16‐F10‐PSMA cells at unconjugated anti‐PSMA VHH amounts of 1.5 and 15 nmol. For these concentrations, we reached comparable absolute values for uptake and transfection as those exhibited by R2‐LNPs treatment. On the contrary, no changes were observed in anti‐PSMA LNPs uptake and transfection upon pre‐treatment with unconjugated VHH in B16‐F10 cells. No statistically significant differences were found regarding uptake (Figure 3C) and transfection for R2‐LNPs (Figure 3D) in both cell lines when comparing 0, 1.5, and 15 nmol of anti‐PSMA VHH pre‐treatment conditions. These results validated that the observed enhanced uptake and transfection of anti‐PSMA LNPs in PSMA^+^ cells are PSMA‐receptor mediated.

### 2.4 Anti‐PSMA LNPs Show Targeted mRNA Delivery to PCa Cells in a Zebrafish Xenograft Model

After the in vitro validation of our targeted LNPs in various PSMA^+^ and PSMA^−^ cell lines we wanted to find out if the enhanced LNP uptake and mRNA transfection were also replicable in vivo. To investigate this, we opted to examine the behavior of anti‐PSMA and R2 DSPE‐Rhodamine LNPs encapsulating BFP‐coding mRNA in *Casper* mutant zebrafish (*Danio rerio*) embryos xenografted with LNCaP cells. Zebrafish (ZF) embryos offer the unique opportunity to investigate LNP dynamics, mRNA delivery, and particle fate at a single‐particle scale in a transparent model organism where it is possible to engraft human tumor cells.^[^ ^33^, ^34^, ^35^, ^36^ ^]^ The rapid development and accessibility of the ZF embryos allow for real‐time visualization and screening of fluorescently labeled tumor cells and LNP formulations, facilitating the study of drug delivery mechanisms for cancer research.^[^ ^37^ ^]^ Given that cancer metastases are the primary cause of death for most PCa patients^[^ ^38^ ^]^ and that effective treatments are available for localized PCa^[^ ^39^ ^]^ we wanted to test our PSMA targeting‐LNPs in a metastatic PCa ZF model.

**Figure 4:**
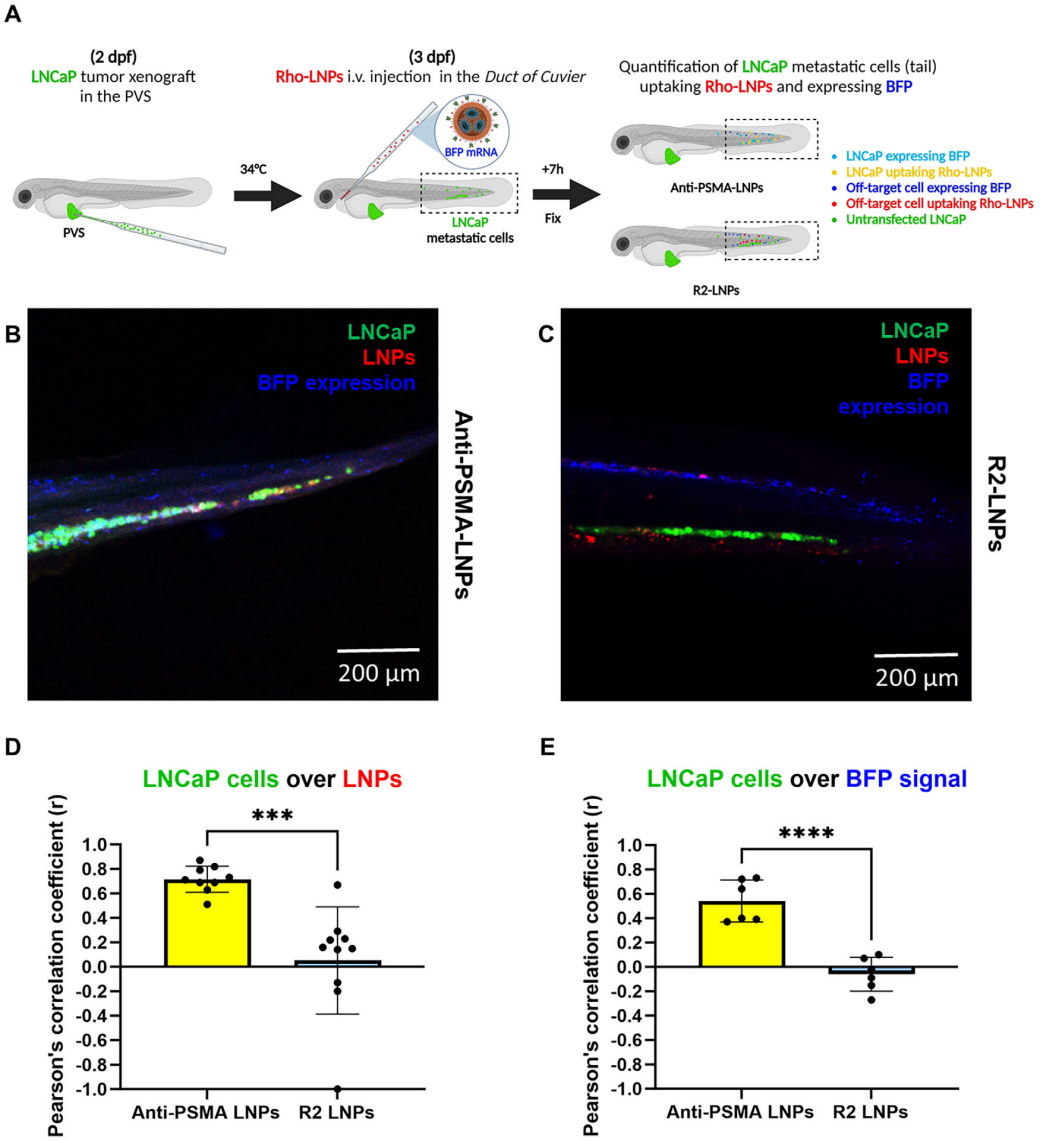
Anti‐PSMA LNPs show targeted mRNA delivery to metastatic PCa cells in a Zebrafish Xenograft model. A) Time frame of in vivo experiments in ZF Casper embryos. LNCaP (PSMA^+^) cells were engrafted in the PVS at 2 dpf. LNPs were i.v. injected in the Duct of Cuvier at 3 dpf. After 7 h, embryos were fixed with 4% PFA in egg‐water. Imaging was performed using a Leica SP5 confocal microscope. Color legend: Green = LNCaP cells marked with Membright‐488. Red = LNPs containing 0.2% DSPE‐Rhodamine. Blue = BFP protein expression. Injected dose: ≈1 mg kg^−1^ mRNA. Injection volume: 2 nL. PVS = perivitelline space; i.v. = intravenous; ZF = zebrafish; dpf = day post fertilization. B,C) ZF caudal biodistribution of anti‐PSMA LNPs (B, red) or R2‐LNPs (C, red) and respective merge with LNCaP cells (green) and BFP expressing cells (blue) in the tail. D,E) Pearson's correlation coefficients (r) of LNCaP cells (green) over LNPs (red) signals (D), and LNCaP cells (green) over BFP (blue) signals (E) comparing anti‐PSMA LNPs vs R2‐LNPs conditions, respectively. Anti‐PSMA LNPs showed enhanced targeting (B,D) and functional BFP mRNA transfection in LNCaP cells (B,E) when compared to R2‐LNPs (C,D and C,E). Pearson's colocalization coefficients (r) between the different channels were calculated on Fiji‐ Image J software. Significance of the differences between groups was determined by a two‐tailed unpaired *t*‐test, for datasets previously tested with a normal (Gaussian) distribution by Shapiro–Wilk test. Data represent mean ± SD of *n* = 10 (D), *n* = 6 (E) embryos per condition. ****, *p*‐value < 0.0001; ***, *p*‐value < 0.001.

We engrafted LNCaP cells in the perivitelline space (PVS) of the ZF embryos at 2 days post fertilization (dpf), keeping embryos overnight at 34 °C to allow LNCaP cells to migrate toward the caudal region (**Figure** **4**A). A dose corresponding to ≈1 mg kg^−1^ mRNA LNPs was intravenously (i.v.) injected in the *Duct of Cuvier*, and after 7 h embryos were PFA‐fixed and imaged for LNP biodistribution and functional mRNA transfection. The ZF caudal biodistribution (red) and functional mRNA transfection (blue) of anti‐PSMA LNPs (Figure 4B), compared to R2‐LNPs (Figure 4C), demonstrated significantly enhanced uptake (Figure 4D) and mRNA transfection (Figure 4E) in LNCaP cells (green). Various examples of the tail pictures that we used to quantify signal colocalization for anti‐PSMA LNPs (Figure S5A–D, Supporting Information) and for R2‐LNPs (Figure S5E,F, Supporting Information) can be visualized in the different channels (red, blue, green and bright field) and their respective merged combinations. For a complete overview of the LNP biodistribution in ZF embryos, 20x images were recomposed together to visualize the entire embryo (Figure S5G,H, Supporting Information). As can be seen in the entire embryo recomposed images, most of the LNPs ended up in the tail region and only a small percentage reached the PVS, where the LNCaP cells were engrafted. More detailed images from the caudal region revealed a tendency for both Anti‐PSMA‐LNPs and R2‐LNPs to accumulate in the cells near the vasculature (Figure S5I–M, Supporting Information).

### 2.5 Anti‐PSMA LNPs Show Enhanced Uptake in PSMA^±^ Mouse Xenografts Upon Systemic Administration

After successfully demonstrating enhanced accumulation and functional mRNA transfection of our targeted LNPs in a xenografted ZF model, we sought to determine if these effects would persist in a mouse xenograft model. Since mice share more of their genes with humans, with ≈85% of protein‐coding regions being identical on average,^[^ ^40^ ^]^ compared to ≈70% between humans and zebrafish,^[^ ^41^ ^]^ they were a logical choice for further testing. Given that LNPs are known to interact with the immune system upon systemic administration.^[^ ^42^, ^43^ ^]^ it was essential to evaluate their performance in immunocompetent mice.

To study the biodistribution and functional transfection of our anti‐PSMA LNPs, we subcutaneously implanted B16‐F10‐PSMA cells into the lateral flank of C57BL/6NCrl mice, which is the same strain from which the original parental cell line of these tumors was derived. For this purpose, when the engrafted tumors reached 300 mm^3^ in volume, we intravenously injected 0.5 mg kg^−1^ of firefly luciferase mRNA encapsulated in DSPE‐Cy5‐labeled anti‐PSMA or R2 LNPs (0.2% DSPE‐PEG(2000)‐VHH post‐inserted) or PBS.

**Figure 5:**
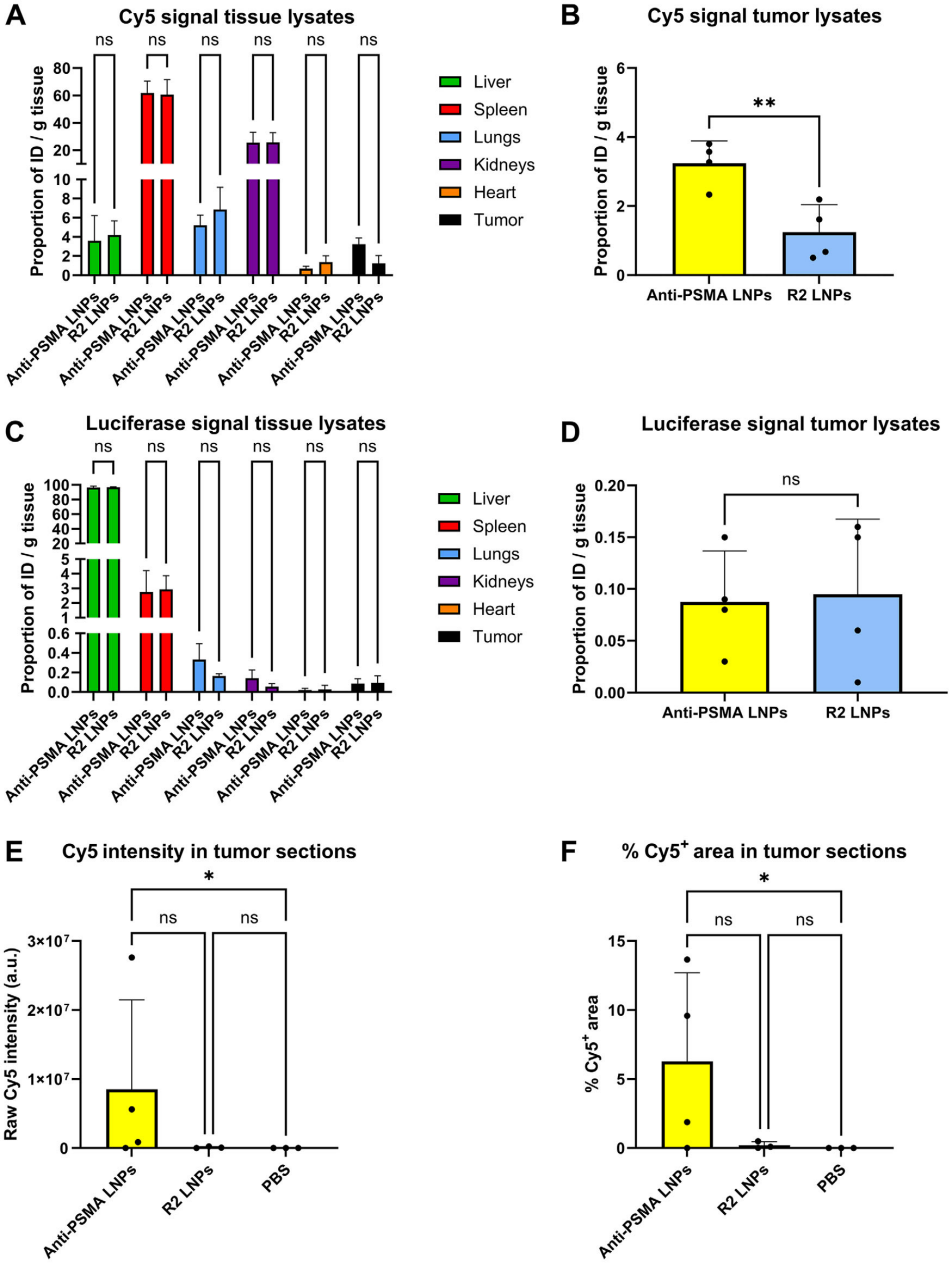
Anti‐PSMA LNPs show enhanced tumor accumulation but not functional mRNA transfection in mouse tumors after 24 h. A,B) Measurement of Cy5 signal in organ tissue lysates (B) and in tumor lysates alone. C,D) Measurement of luciferase signal in organ tissue lysates (D) and in tumor lysates alone. Biodistribution of LNPs by quantification of Cy5 signal (A,B) and functional mRNA transfection by quantification of luciferase signal (C,D) are expressed as percentage of the injected dose per gram (% ID/g) of tissue. E,F) Quantification of Cy5 signal from tumor sections of animals treated with anti‐PSMA LNPs, R2 LNPs or PBS determined by Cy5 raw intensity (E) and % of Cy5^+^ area (F). For the graphs (A,C) a two‐way ANOVA with Šidák correction for multiple comparisons test was performed comparing the mean signals from each organ between the treatment groups. For the graphs (B,D) statistical differences were assessed with a two‐tailed unpaired *t*‐test. For the graphs (E,F) statistical differences were assessed with a One Way Anova nonparametric Kruskal–Wallis test with Dunn's multiple comparisons. Data represent mean ± SD (*n* = 4 animals) for graphs A–D and *n* = 3‐4 animals for graphs E and F. ****, *p*‐value < 0.0001; ***, *p*‐value < 0.001; **, *p*‐value < 0.01; *, *p*‐value < 0.05; ns: no significant difference.

Tissue distribution was assessed 24 h post LNP administration through Cy5 fluorescence spectroscopy of tissue lysates (**Figure** **5**A). Both anti‐PSMA and R2 LNPs mainly accumulated in the spleen (≈60% ID/g), followed by the kidneys (≈20% ID/g), the lungs (5–7% ID/g) and the liver (3–4% ID/g) without any significant differences between treatments. As for tumor accumulation (Figure 5B), anti‐PSMA LNPs (3.24% ID/g) exhibited a significant increase when compared to R2 LNPs (1.24% ID/g). When we normalized the tumor tissue lysates by protein content instead of by tissue weight, we also observed a notable increase in tumor accumulation for anti‐PSMA LNPs (Figure S6A, Supporting Information).

When it comes to luciferase expression (Figure 5C), the vast majority of the signal came from the liver (96–97% ID/g), and there were no significant differences between treatments. Upon closer examination of tumor luciferase expression (Figure 5D), the level of expression was quite low, with ≈0.09% ID/g in tumors for both anti‐PSMA and R2 LNP‐treated animals. Normalizing the tumor tissue lysates by protein content confirmed again that there were no significant differences in protein expression between treatments (Figure S6B, Supporting Information). Additionally, we charted the ratios of tumor‐to‐liver and tumor‐to‐spleen for LNP accumulation (Figure S6C,D, Supporting Information) and mRNA transfection (Figure S6E,F, Supporting Information). Once again, anti‐PSMA LNPs yielded higher tumor/liver and tumor/spleen ratio values for LNP accumulation compared to R2‐LNPs. No significant differences were found between anti‐PSMA LNPs and R2‐LNPs regarding tumor‐to‐liver and tumor‐to‐spleen ratio values for mRNA transfection.

Tumor tissues were cryosectioned for histological analysis, in order to complement the tissue lysates analysis for quantification of LNP uptake within the tumors. For that purpose, 15 images of each of 2 distal tumor cryosections from each tumor were captured at 20x magnification and quantified by means of a custom‐made script for Cy5 raw intensity (Figure 5E) and % of Cy5^+^ area (Figure 5F). Representative pictures of the tumor sections for each of the treatment groups (Figure S7A–C, Supporting Information) can be found together with an example of quantification of Cy5 signal by subtraction of Cy3 autofluorescence (Figure S7D, Supporting Information). From our analysis we could infer how in tumors from animals treated with anti‐PSMA LNPs there was on average a higher Cy5 intensity (83‐fold, Figure 5E) and % of Cy5^+^ area (31‐fold, Figure 5F) compared to R2 LNPs; although not statistically significant.

Finally, a portion of the extracted tumors was processed into single cell suspensions to determine LNP uptake in different cellular populations, namely immune cells (CD31^−^/CD45^+^), endothelial cells (CD31^+^/CD45^−^) and CD31^−^/CD45^−^ cells, where we expected to find the B16‐F10‐PSMA tumor cells. We first characterized the abundance of each cell population in relation to single living cells, and we inferred that ≈60% of the cells were immune cells, ≈0–0.1% were endothelial cells, and ≈35–40% were CD31^−^/CD45^−^ cells (Figure S8A–C, Supporting Information). Regarding LNP accumulation (Cy5 MFI) in immune and endothelial cells, no significant differences were appreciated between treatments (Figure S8D,E, Supporting Information). Focusing on CD31^−^/CD45^−^ cells, we could see a trend toward enhanced accumulation for anti‐PSMA LNPs compared to R2 LNPs (1.87‐fold MFI increase, Figure S8F, Supporting Information).

## 3 Discussion

In this study, we successfully demonstrated the potential of PSMA‐targeted LNPs in delivering mRNA specifically to PSMA^+^ cancer cells in vivo. Our approach employed anti‐PSMA nanobodies post‐inserted onto LNPs through click‐chemistry with a PEG‐lipid conjugate. Through gel electrophoresis analysis of the VHH‐lipid click chemistry products, (Figure S1A,B, Supporting Information) Anti‐c‐Myc dot blot of the final targeted‐LNP solution (Figure S1C, Supporting Information) and dSTORM analysis (Figure 2), we confirmed that 80% of anti‐PSMA LNPs displayed at least one VHH molecule on their surface, indicating successful nanobody decoration.

The specificity and efficacy of our targeted LNPs were validated through multiple experiments. In vitro, these anti‐PSMA LNPs demonstrated enhanced uptake and transfection in PSMA^+^ cancer cells, such as B16‐F10‐PSMA (Figure 3A,B) and LNCaP (Figure S3, Supporting Information), compared to nontargeted LNPs and PSMA^−^ cells (B16‐F10 and DU145). Furthermore, our in vitro LNP uptake and transfection results with previous incubation of unconjugated VHH demonstrated that the enhanced LNP uptake and mRNA transfection of anti‐PSMA LNPs in PSMA^+^ cells are PSMA‐receptor mediated (Figure 3C,D).

Moving to in vivo studies, this specificity and efficacy were further confirmed in a zebrafish PCa xenograft model, where targeted LNPs showed enhanced accumulation and transfection in PSMA^+^ prostate cancer cells that had metastasized to the caudal region (Figure 4D,E). Despite relatively high LNP accumulation, BFP expression (mRNA transfection) was rather limited in tumor cells (Figure 4B; Figure S5A–D, Supporting Information). Conversely, the presence of nongreen cells exhibiting BFP expression was prominent. The morphology and tissue distribution of these BFP‐expressing cells suggest they may be scavenger immune cells (e.g., caudal patrolling macrophages); however, this identification remains hypothetical and requires further validation. Future studies using transgenic ZF strains with GFP‐expressing immune cells might elucidate the nature of these off‐target cells. Additionally, since we did not use a ZF strain with fluorescently labeled macrophages, we cannot rule out the possibility that part of the LNCAP‐associated signal might actually be debris phagocytosed by macrophages. If this were the case, a portion of the BFP signal co‐localizing with the green LNCAP signal could also originate from macrophages that have phagocytosed LNCAP cellular debris.

Furthermore, since imaging occurred 7 h post‐LNP administration due to the slowing heartbeat of immobilized ZF embryos, a potential explanation for our observations could be that LNPs might adhere to the LNCaP cell membranes and take longer to endocytose, unlike macrophages which rapidly phagocytosed them. Moreover, our observations revealed major localization of anti‐PSMA LNPs in LNCaP cells and macrophages situated proximal to the caudal ZF vasculature (Figure S5I–M, Supporting Information), indicating reduced tissue penetration capacity of the LNPs.

We also extended our investigation to a mouse PSMA^+^ xenograft model to evaluate the effects of systemic administration of these LNPs in a mammalian model. Even though the results indicated a clear trend toward enhanced LNP uptake in PSMA^+^ tumors as demonstrated by tissue lysates (Figure 5B), histology analysis (Figure 5E,F) and flow cytometry (Figure S8, Supporting Information), these did not lead to increased tumor luciferase expression (Figure 5D). We hypothesize that the reason for this discrepancy from our previous in vitro and ZF experiments, where increased uptake consistently led to increased mRNA transfection, might be linked to the highly necrotic status of the tumors. We theorized that necrotic B16‐F10‐PSMA cells would still express PSMA on their cell membranes and thus interact with anti‐PSMA LNPs, but due to their necrotic condition, this enhanced LNP capture would not lead to increased luciferase expression.

As solid tumors grow to a certain size, their inner regions often have limited blood supply, subjecting the cells to metabolic stresses such as hypoxia and nutrient deficiency, which can lead to necrosis.^[^ ^44^, ^45^, ^46^ ^]^ For this purpose we investigated if the tumor size, measured with an electronic caliper at the moment of euthanasia, had any influence on cell viability, LNP uptake or functional mRNA delivery. Our results determined that there was no major influence of tumor size in cell viability, LNP uptake or functional mRNA delivery (Figure S9, Supporting Information). We then looked deeper into the necrotic status of the tumors through different methods; namely zombie aqua and trypan blue staining in tumor cell suspensions and by having expert pathologists determine the percentage of necrosis based on two independent H&E cryosections of each tumor (Figure S10, Supporting Information). Our findings indicate that tumors from animals treated with anti‐PSMA LNPs exhibit a trend in increased percentage of necrosis compared to R2‐LNPs and PBS treated animals (Figure S11, Supporting Information). To further investigate whether the increased tumor necrosis observed in anti‐PSMA LNP‐treated animals was a consequence of LNP uptake, we examined liver tissue— an organ that exhibits similar LNP accumulation per gram of tissue to the tumor and shows the highest mRNA expression—24 h post‐injection. H&E‐stained liver sections from mice treated with either anti‐PSMA LNPs, R2‐LNPs or PBS were assessed by two independent pathologists and no histological evidence of necrosis or major toxicity signs were observed (Figure S12, Supporting Information). These findings support the hypothesis that the differences in tumor necrosis levels were possibly circumstantial and not caused by LNP accumulation.

Furthermore, to evaluate whether mRNA delivery efficiency is influenced by the tumor microenvironment (TME), we conducted a correlation analysis between the extent of tumor necrosis and luminescence signal in tumors from animals treated with either anti‐PSMA LNPs or R2‐LNPs. As shown in Figure S13 (Supporting Information), an inverse correlation was observed in both treatment groups: greater tumor necrosis was associated with lower luciferase activity. This indicates that reduced mRNA expression in certain tumors was primarily attributable to their necrotic state, rather than inefficient LNP‐mediated delivery.

Moreover, when analyzing the tumor suspension flow cytometry results in detail, we noticed that the percentage of Cy5^+^ cells for both LNP treatments was three–sevenfold lower in living cells (Figure S8G, Supporting Information) than in dead cells (Figure S8H, Supporting Information). The same trend was observed for both LNP treatments when comparing the Cy5 MFI of CD31^−^/CD45^−^ living cells (Figure S8F, Supporting Information) with that of CD31^−^/CD45^−^ dead cells (Figure S8I, Supporting Information), with the former being six–sevenfold lower. Altogether, these findings suggest that LNPs to a large extent associated to dying/dead cells. It is noteworthy to mention that most of the Cy5 signal from tumor cryosections came from areas with low DAPI intensity, potentially denoting that the LNPs were mainly found in necrotic areas. These results underscore the importance of tumor viability for delivery efficacy and highlight the challenges of targeted nanoparticle delivery within heterogeneous TMEs. Further research would be needed to ultimately confirm whether, in a less necrotic tumor xenograft mouse model, anti‐PSMA LNPs exhibit enhanced uptake leading to increased transfection, and to unequivocally determine whether the observed necrosis in this study was not caused by anti‐PSMA LNP uptake.

Another important point to consider is that LNPs formulated with C12‐200 ionizable lipid, although effective at delivering RNA in mice^[^ ^47^, ^48^, ^49^, ^50^ ^]^ and nonhuman primates,^[^ ^47^ ^]^ have been reported to cause liver necrosis in mice at higher doses^[^ ^51^ ^]^ and lack clinical translatability due to poor tolerability. This study used it as a proof of concept for anti‐PSMA LNP formulation, but future research should focus on less immunogenic biodegradable ionizable lipid alternatives.

Inspired by a recent review on the challenges of nanoparticle penetration across tissues,^[^ ^52^ ^]^ an overview of the envisioned sequential barriers for the systemic delivery of anti‐PSMA LNPs to tumors is presented below: 1) Possible loss of targeting capacity due to LNP protein corona formation: The protein corona, formed when nanoparticles interact with plasma proteins, alters their surface, reducing targeting efficiency and increasing clearance by the mononuclear phagocytic system. Strategies like using stealth polymers, and protein corona shields can potentially be employed to modulate this effect and preserve targeting ability.^[^ ^53^ ^]^ 2) RES‐mediated clearance of LNPs from the liver and the spleen: The RES efficiently captures and clears LNPs from the circulation, reducing their circulation time and tumor accumulation.^[^ ^54^ ^]^ 3) Extravasation through the endothelium into the tumor: LNPs must pass through the endothelial cell lining of blood vessels. However, in tumor vessels, proinflammatory cytokines often increase endothelial cell gaps.^[^ ^55^ ^]^ allowing LNPs to move from the bloodstream into the tumor. 4) Penetration through the tumor interstitial space and tumor cell transfection: The tumor stroma is characterized by a modified extracellular matrix (ECM) with elevated presence of fibroblasts that produce growth factors, chemokines, and adhesion molecules, all of which contribute to the difficulty of nanoparticles penetrating the tissue.^[^ ^52^, ^56^ ^]^ To take it one step further, even macromolecules such as IgGs typically accumulate at the outer edges of the tumor rather than penetrating to its core.^[^ ^57^ ^]^

In our studies, while anti‐PSMA LNPs demonstrated enhanced uptake and delivery of mRNA to PCa cells in ZF, the limited tissue penetration and off‐target uptake by immune cells suggest there is still room for optimization. Strategies to enhance cancer cell uptake by reducing immune cell interactions with LNPs could be crucial for advancing LNP‐based therapies for solid tumors, but systemic administration of mRNA‐LNPs targeting solid tumors presents itself with the above mentioned intrinsic difficulties. The TME plays a critical role in shaping nanoparticle biodistribution and cellular uptake within the tumor. In addition to tumor viability, factors such as the density and phenotype of immune cells, extracellular matrix composition, and vascular permeability can all modulate LNP performance. The TME is populated by a significant number of tumor‐associated macrophages (TAMs), primarily of the M2 phenotype, which contribute to pro‐tumor activities.^[^ ^58^ ^]^ Recent studies have demonstrated that TAMs and other phagocytes take up a big proportion of the injected LNPs, limiting their access to tumor cells.^[^ ^59^ ^]^ One potential strategy to mitigate nonspecific uptake by TAMs could be the incorporation of “don't‐eat‐me” signals like CD47 motifs in the LNP surface. A recently published study has shown that the combination of CD47 peptides and antibodies on the surface of LNPs lead to reduced phagocytic clearance and off‐target effects and improved efficiency for cell‐specific delivery to endothelial cells, T cells, or hematopoietic stem cells (HSCs).^[^ ^60^ ^]^

Alternatively, an increasing number of studies suggest that rather than avoiding nanoparticle uptake by immune cells in the TME, directly targeting and reprogramming these cells may offer a promising new avenue for cancer immunotherapy.^[^ ^59^, ^61^, ^62^, ^63^, ^64^ ^]^ LNP‐mediated immunotherapies, such as CAR‐T‐cell or CAR‐macrophage therapies, could offer a promising approach by delivering chimeric antigen receptor (CAR) mRNA or DNA to immune cells,^[^ ^65^ ^]^ either in circulation (e.g., T cells) or within the TME (e.g., TAMs). As an example of this approach, genetically modifying immune cells to express a PSMA binder could potentially be used to target them toward CRPC cells. This strategy has the potential to overcome barriers like limited tumor penetration and harness immune cells as therapeutic agents, turning off‐target sinks for LNPs from a liability into an opportunity.

This study lays the groundwork for developing more effective and safer anti‐PSMA mRNA LNPs, with the ultimate goal of improving treatment for CRPC. By addressing the highlighted challenges, we can work toward creating personalized therapeutic strategies that not only effectively target PCa but also reduce collateral damage to healthy tissues. Ultimately, this line of research holds the promise of extending survival rates and improving the quality of life for patients with CRPC.

## 4 Experimental Section

### Materials

1,1′‐[[2‐[4‐[2‐[[2‐[bis(2‐hydroxydodecyl)amino]ethyl](2‐hydroxydodecyl)amino]ethyl]‐1‐piperazinyl]ethyl]imino]bis‐2‐dodecanol (C12‐200) (GVK Biosciences, India), cholesterol (Merck, Darmstadt, Germany), 1,2‐distearoyl‐sn‐glycero‐3‐phosphocholine (DSPC) (Lipoid, Ludwigshafen am Rhein, Germany), 1,2‐dimyristoyl‐rac‐glycero‐3‐methoxypolyethylene glycol‐2000 (DMG‐PEG2000), 1,2‐distearoyl‐sn‐glycero‐3‐phosphoethanolamine‐N‐[methoxy(polyethylene glycol)‐2000] (DSPE‐PEG2000), 1,2‐distearoyl‐sn‐glycero‐3‐phosphoethanolamine‐N‐Cyanine 5 (18:0 Cy5 PE), 1,2‐dioleoyl‐sn‐glycero‐3‐phosphoethanolamine‐N‐lissamine rhodamine B sulfonyl (18:1 Liss Rhod PE) (Avanti Polar Lipids, Alabama, USA), were dissolved in 100% ethanol (Merck). 1,2‐distearoyl‐sn‐glycero‐3‐phosphoethanolamine‐N‐[dibenzocyclooctyl(polyethylene glycol)‐2000] (DSPE‐PEG(2000)‐DBCO) (Avanti Polar Lipids) was dissolved in DMSO. Gly3‐azide (H‐(Gly)3‐Lys(N3)‐OH*HCl, Triglycyl‐epsilon‐azido‐L‐lysine hydrochloride) was purchased from Iris Biotech GMBH (Marktredwitz, Germany). Talon Superflow was obtained from Cytiva Life Sciences (Massachusetts, USA). Imidazole was purchased from Merck Millipore (Massachusetts, USA). Anti‐c‐Myc antibody (clone 9E10) was produced in‐house.

### IVT mRNA Synthesis

Firefly luciferase mRNA, BFP mRNA, EGFP mRNA, and EGFP mRNA labeled with Cy5 were produced inhouse. DNA sequences encoding luciferase, BFP, or EGFP were cloned into a proprietary mRNA production plasmid containing a T7 RNA polymerase promoter, a 5' untranslated region (UTR) derived from HIV gp160, a 3' UTR derived from mouse α‐globin, and a 120‐nucleotide poly(A) tail. Capped mRNA was synthesized using the VENI all‐in‐one mRNA Synthesis Kit (Leish Bio, The Netherlands) following the manufacturer's instructions. Briefly, the reaction mixture contained 1× T7 reaction buffer, 10 mm DTT, 5 mm each of ATP, CTP, GTP, and N1‐methylpseudo‐UTP (m1ΨTP), 50 ng µL^−1^ template DNA, and 5 mm cap 1 analog. For Cy5‐labeled mRNA, Cy5‐UTP (Enzo Life Sciences, New York, USA) was incorporated at a 1:3 ratio with m1ΨTP during transcription. Reactions were incubated for 2 h at 37 °C followed by treatment with 2 U µL^−1^ TURBO DNase (Thermo Fisher Scientific, Massachusetts, USA) for 15 min at 37 °C to eliminate the DNA template. The mRNA was purified by precipitation with an equal volume of precipitation solution, followed by two washes with cold 70% ethanol. The final mRNA pellet was then resuspended in nuclease‐free water. mRNA concentration was determined using a DS‐11 Spectrophotometer (DeNovix, Delaware, USA), and the mRNA (500 ng) was heat‐denatured and analyzed by electrophoresis on a 1.5% agarose gel containing 0.01% (v/v) GelRed nucleic acid stain. GeneRuler 1 kb Plus DNA Ladder (Thermo Fisher Scientific) was used as a referential molecular weight marker.

### Preparation of Cloning Vectors

Anti‐PSMA VHH original sequence was obtained from a previously published study.^[^ ^30^ ^]^ In order to produce anti‐PSMA VHH and R2‐VHH, the previously described protocol was followed.^[^ ^31^ ^]^ A c‐Myc epitope was inserted at the C‐terminal end of the VHH to enable detection. To facilitate sortase‐based modification, VHHs were placed in a vector produced in‐house that included an N‐terminal Shine Dalgarno sequence, a PelB leader peptide, a C‐terminal c‐Myc‐tag connected by a (Gly4‐Ser)2 linker to a LeuPro‐Glu‐Thr‐Gly (LPETG) sortase tag and then followed by a (His)8‐purification tag. The Myc‐tag was used to identify VHH via dot blot. LPETG tag cleavage is essential for enabling azide incorporation, while the HIS‐tag was incorporated to remove any uncut VHH.

### VHH Production and Purification

VHHs were generated in *Escherichia coli* (*E. coli*) strain BL21 pLyss through autoinduction in a bioreactor, following the methods outlined in a prior study.^[^ ^66^ ^]^ The purification of VHHs was carried out using immobilized metal affinity chromatography by incubation with Talon Superflow beads, and the elution of the beads was done in a Tricorn (10/50) Column (Cytiva Life Sciences). The imidazole from the solution was then removed using 7 kD Zebaspin (Thermo Fisher Scientific) columns according to manufacturer's protocol, followed by size exclusion chromatography (HiLoad 26/600 Superdex 600 200 pg) in an ÄKTA pure chromatography system (Cytiva Life Sciences). Based on the AKTA's measurement, the fractions which contained protein were collected and pooled, and the elutions were moved onto Vivaspin 3 kDa columns (Sartorius, Göttingen, Germany) to concentrate the samples. The purity of the resulting VHH preparations was validated by SDS‐PAGE and PageBlue Protein Staining (Thermo Fisher Scientific).

### Sortase A Production

Sortase A was produced in *E. coli* strain BL21 pLyss (Invitrogen, California, USA) using an autoinduction method and subsequently purified following the previously described protocol.^[^ ^32^ ^]^

### LNP Production and Formulation

Lipid nanoparticles were produced using microfluidic mixing with a NanoAssemblr Benchtop system (Precision Nanosystems, Vancouver, Canada). The synthesis involved combining an ethanolic phase containing lipids with an acidic aqueous phase comprising 100 mm sodium acetate (pH 4.0) and mRNA. This formulation took place at a flow rate ratio of 3:1 (aqueous:organic), with a total flow rate of 9.0 mL min^−1^. The lipids, dissolved in 100% ethanol at a concentration of 5–20 mm, consisted of C12‐200, cholesterol, DSPC and DMG‐PEG in molar percentages of 50/38.5/10/1.5, respectively. For the experiments with LNPs containing fluorescent lipids, the employed molar percentage was 0.2% in detriment of cholesterol. Encapsulation was achieved at a w/w ratio (ionizable lipid/RNA) of 24.5. Right after production, the LNPs underwent dialysis at 4 °C against an excess of phosphate‐buffered saline (pH 7.4) using Slide‐a‐Lyzer dialysis cassettes G2 (membrane cutoff 20 kDa) for 16–28 h.

### Click Chemistry Conjugation of VHH to DSPE‐PEG(2000)‐DBCO

To attach nanobodies to the surface of LNPs, the C‐terminal of the nanobodies was first conjugated with DSPE‐PEG(2000)‐DBCO following the previously described method.^[^ ^32^ ^]^ This process involved cleaving an LPETG‐tag in the presence of sortase A and binding a Gly3‐Lys‐azide to the VHH. The reaction mixture, containing 100 µm VHH, 1000 µm Gly3‐Lys‐azide, and 1 µm sortase A, was diluted in TBS pH 7.4 and incubated for 2 h at 25 °C. After the reaction, purification of VHH‐azide complexes from sortase A and unmodified VHHs was performed through Talon Superflow beads incubation. Subsequently, size exclusion chromatography on Zeba spin 7 kDa desalting columns was used to eliminate excess Gly3‐Lys‐azide from the reaction mixture, and the elutions were moved onto Vivaspin 10 kDa columns to concentrate the samples. A small volume of concentrated DSPE‐PEG(2000)‐DBCO dissolved DMSO was then added to the VHH‐azide intermediate dissolved in TBS at molar ratio of 3:1 (lipid: VHH) to allow an overnight reaction at room temperature in a rotating mill.

### Post‐Insertion of DSPE‐PEG(2000)‐VHH onto the Surface of LNPs

The following day, the VHH‐PEG‐lipid complex and LNPs were combined and incubated at 45 °C for 1 h to facilitate post‐insertion. The molar% of VHH‐PEG‐lipid added to the LNPs was normalized in relation to the total lipid concentration of the LNPs, which varied across experiments, ranging from 0.2% to 1%. After the incubation period, the mixture was poured into 100 kDa dialysis cassettes and dialyzed in excess PBS pH 7.4 overnight at 4 °C. Following dialysis, LNPs were subjected to sterilization using 0.45 µm PVDF membrane filters and concentrated using Amicon Ultra‐15 centrifugation filter columns with a membrane cutoff of 100 kDa (Merck) at 2500 g and 4 °C. The purified LNPs were stored at 4 °C and used within 15 days of production.

### LNP Characterization—Dynamic Light Scattering for Size and Polydispersity Index Measurement

Dynamic Light Scattering (DLS) technique using a Zetasizer Nano S instrument (Malvern Panalytical, Malvern, UK) was employed to determine the hydrodynamic diameter of LNPs. A 4 mW HeNe laser emitting at 633 nm was used for the measurements. The LNPs were diluted in Dulbecco's Phosphate‐Buffered Saline (DPBS, pH 7.4), and scattering measurements were conducted at an angle of 173° and a temperature of 25 °C for a duration of 10 s per measurement, repeated ten times. Each specimen underwent three measurements.

### LNP Characterization—Zeta Potential Measurement

The Zeta potential of the particles was measured with a Zetasizer Nano Z (Malvern Panalytical) after calibrating the device using a Zeta Potential Transfer Standard (Malvern Panalytical). The LNPs were diluted in 10 mm HEPES solution at pH 7.4, and each sample underwent a minimum of three separate measurements.

### mRNA Quantification and Encapsulation Efficiency Assessment

Total mRNA concentration was measured using the Quant‐It Ribogreen RNA Assay kit (Thermo Fisher Scientific) with 2% (v/v) Triton X‐100 in TE buffer. Free mRNA concentration was determined separately in TE buffer alone. Lysed LNPs produced a fluorescent signal indicating total mRNA (µg µL^−1^), while nonlysed samples showed free nonencapsulated mRNA (µg µL^−1^). Reference calibration curves were created for both 2% Triton X‐100 and TE buffer to quantify the mRNA concentration. The encapsulated mRNA concentration was calculated by subtracting the free mRNA concentration from the total, and then used to determine the percentage of encapsulated mRNA according to this formula: ((Total mRNA – Free mRNA)/Total mRNA) × 100.

### LNP Characterization—Dot Blot for VHH Detection in LNPs

Dot blotting was employed for the detection of VHHs in the LNP mixture. Twenty microliters of the final targeted LNPs samples (0.2% DSPE‐PEG(2000)‐VHH post‐inserted) were spun down in Vivaspin 100 kDa columns until all the liquid had filtered through. After reconstitution of the membrane with 5 µL of DPBS, 2 µL of the filtered sample, and 2 µL of the nonfiltered reconstituted sample were directly pipetted onto an Amersham Protran Western blotting nitrocellulose membrane, pore size 0.45 µm (Merck). Subsequently, the membrane was air‐dried for 5 min and subjected to an overnight incubation with blocking buffer (LI‐COR Biosciences, Nebraska, USA) diluted in a 1:1 ratio with TBS‐Tween 0.1% (TBS‐T) at 4 °C. The following day, the blocking buffer was removed, and the primary anti‐c‐Myc antibody (clone 9E10) was applied at a dilution of 1:2000 in blocking buffer (1:1 with TBS‐T). The membrane was then incubated with the primary antibody for 2 h on an orbital shaker at room temperature, followed by thorough washing with TBS‐T thrice for 5 min each, and once with TBS. Subsequently, the secondary antibody, IRDye 800CW Goat anti‐Mouse IgG (LI‐COR Biosciences, diluted 1:10 000 in blocking buffer) was applied and allowed to incubate for 1 h at room temperature. The membrane underwent another round of washing using the same protocol as with the washing of the first antibody and was then imaged using an Odyssey M Imager (LI‐COR Biosciences) at the wavelengths of 700 nm (mRNA‐Cy5.5‐labeled) and 800 nm (VHH).

### LNP Characterization—Gel Electrophoresis Analysis for VHH Click Chemistry and Post Insertion Characterization

Samples from the different steps of the VHH click chemistry to DSPE‐PEG(2000)‐DBCO and subsequent post‐insertion to LNPs (0.2% DSPE‐PEG(2000)‐VHH post‐inserted) were collected and mixed with 4X Laemmli loading buffer containing DTT. The samples were heated at 95 °C for a minimum of 5 min, spun down, and placed on ice. The samples were loaded onto 4–12% gradient Bis‐Tris polyacrylamide gels (Thermo Fisher Scientific) and subjected to gel electrophoresis for separation. Following electrophoresis, the gel was stained for total proteins with PageBlue Protein Staining Solution (Thermo Fisher Scientific) for 30 min.

### LNP Characterization—Antibody Conjugation for dSTORM Imaging

Sodium azide was removed from in‐house‐purified recombinant anti‐Myc and mouse IgG control antibodies (15H6, SouthernBiotech) using 10 kDA MWCO ultrafiltration vials (Biotium). Afterward, these antibodies were resuspended in PBS in a 1 mg mL^−1^ final concentration. Ten micrograms per antibody was labelled with 40 µg mL^−1^ Alexa Fluor 488 – NHS in 100 mm borate buffer pH 8.6 overnight at 4 °C. Next, the labeled antibodies were purified from free dye using 7 kDa MWCO 0.5 mL Zeba Dye and Biotin Removal Spin Columns (Thermo Fisher Scientific). Conjugated antibodies were used immediately or stored at 4 °C.

### LNP Characterization—dSTORM Imaging and Analysis of LNPs

Using ultrasonication twice in milliQ water for 30 min and once in 1 m KOH for 30 min, coverslips (#1.5H, Marienfeld) were cleaned. After drying the coverslips with N_2_ gas, 50‐well CultureWell gaskets (Grace Bio‐Labs, Oregon, USA) were attached to create independent 10 µL wells on the coverslips. Cy5‐DSPE labelled PSMA‐LNPs and nontargeted‐LNPs were immediately added to the coverslip in a 1:10 dilution overnight at 4 °C. Subsequently, LNPs were blocked in 5% BSA for 30 min at room temperature and incubated overnight at 4 °C with 50 µg mL^−1^ of primary conjugated antibody in 5% BSA. After two quick washes with PBS, OxEA pH 8–8.5 imaging buffer was applied. Prior to imaging, channel mapping was executed using a slide containing 0.1 µm TetraSpeck beads (Thermo Fisher Scientific). TIRF acquisition was performed with a Nanoimager‐S (ONI) equipped with a 100x oil‐immersion objective (Olympus, numerical aperture 1.4) and 405 nm (150 mW), 488 nm (1 W), 561 nm (500 mW), and 640 nm (1 W) lasers. In brief, 2500 frames were acquired with 35% power of 640 nm laser and 30% power of 488 nm laser in a sequential manner.

### LNP Characterization—dSTORM Analysis

The collaborative discovery (CODI) online analysis tool (https://alto.codi.bio/, ONI) was used for the filtering of localizations and HDBSCAN cluster analysis. In brief, localizations were filtered on frame index for the 640 nm (100–2500) and 488 nm (2600–5000) channels. Furthermore, the following filtering thresholds were applied for both channels: photon count >200, sigma 50–300 nm, *p*‐value > 1e^−6^ – 0.2, localization precision 0–20 nm. The following cluster parameters were applied: area < 80 000 nm^2^, skew <2.6, kappa: >23 nm. Localizations were included in counting within a 120 nm radius of the centroid of the cluster. Positivity thresholds of localizations per cluster were set at 2. The number of Alexa Fluor 488^+^ clusters was expressed as a percentage of the Cy5^+^ clusters.

### In Vitro Transfection Experiments—Cell Culture Conditions

B16‐F10, a melanoma cell line which was originally isolated from mice, was used as a negative control for PSMA expression in targeting assays. B16‐F10‐PSMA, a stable cell line, used as a positive control for PSMA expression, was generated by transfection of B16‐F10 cells with the human PSMA gene in a pcDNA3 vector following the strategy described.^[^ ^67^ ^]^ The B16‐F10‐PSMA cell line was kindly provided by Wytske Van Weerden from Erasmus MC Rotterdam. B16‐F10‐PSMA were cultured in Dulbecco's Modified Eagle Medium (DMEM) supplemented with L‐Glutamine (Thermo Fisher Scientific), 20 µm 2‐mercaptoethanol (Merck), 1.2 µg mL^−1^ G418 sulfate (BioIVT, New York, USA) for plasmid selection and 10% fetal bovine serum (FBS)(Merck). DU145 and B16‐F10 cells were cultured in Dulbecco's Modified Eagle Medium (DMEM) supplemented with L‐Glutamine (Thermo Fisher Scientific) and 10% fetal bovine serum (FBS) (Merck). LNCaP cells were cultured in Roswell Park Memorial Institute (RPMI) 1640 Medium (Thermo Fisher Scientific), also supplemented with 10% FBS. All cell lines were cultured in a Biosafety level 1 (BSL1) laboratory in the presence of 100 µg mL^−1^ streptomycin and 100 U mL^−1^ penicillin (Thermo Fisher Scientific) under standard conditions at 37 °C and 5% CO_2_.

### In Vitro LNP Transfection Experiments

In separate 96‐well flat‐bottom plates, 30 000 cells of LNCaP and DU145 and 5000 cells of B16‐F10 and B16‐PSMA were seeded. After 24 h, the culture medium was changed for fresh culture medium containing 10% nonheat‐inactivated FCS. At that point, LNPs encapsulating Cy5‐labeled EGFP mRNA were added to the wells at a concentration of 50–200 ng encapsulated mRNA per well. The cells were then incubated for 24 h at 37 °C and 5% CO_2_. The cells were then washed twice with DPBS, trypsinized and resuspended in 250 µL DPBS 2%FBS. The well content from all the wells in the plate was moved into 96 U‐bottom well plates (Greiner, Kremsmünster, Austria), where uptake (Cy5) and functional transfection (EGFP) were analyzed on a FACS Canto II flow cytometer (Becton, Dickinson and Company, New Jersey, US). Results were shown as a percentage of Cy5/EGFP^+^ cells and as mean fluorescent intensity (MFI) of Cy5 or EGFP. To determine the gating for % of Cy5/EGFP^+^, PBS‐treated cells were used. The measured MFIs for Cy5/EGFP were corrected by subtracting the MFIs obtained for PBS‐treated cells. Data represent mean ± SD (*n* = 3 wells) with at least 5000 cells per well.

### PSMA Expression Analysis

This experiment was employed as a validation to ensure the expression of PSMA in the PSMA^+^ cell lines. The cells were rinsed with warm PBS to eliminate dead cells before adding warm full medium. A cell scraper was used to detach the cells from the flask surface, and the resulting medium containing floating cells was transferred to a 15 mL tube and centrifuged at 300 g for 5 min. Subsequently, the supernatant was discarded prior to resuspending the cell pellet in cold PBS containing 1% sodium azide and 3% bovine serum albumin (BSA). Two volumes comprising 6×10^5^ cells each were diluted up to 1 mL with cold PBS containing 1% sodium azide and 3% BSA. Both tubes underwent a 30‐min incubation period in darkness with a PSMA‐APC antibody (Biolegend, California, USA) (diluted 1:333), or with IgG APC‐H7 (BD Biosciences, New Jersey, USA) (diluted 1:333), which served as a negative control. The cells then underwent two cold PBS 1% sodium azide 3% BSA washes by centrifugation at 4 °C, with the supernatant being discarded between washes. Subsequently, the pellets were suspended in PBS 1% sodium azide 3% BSA before being analyzed for PSMA/IgG expression using the APC‐A channel on a FACS Canto II flow cytometer (Becton, Dickinson and Company). Data represent mean ± SD (*n* = 3 wells) with at least 5000 cells per well.

### Anti‐PSMA VHH Dose‐Dependent Uptake Blockade

In order to give proof that anti‐PSMA LNPs uptake is PSMA mediated, a transfection assay was conducted with different concentrations of spiked unconjugated anti‐PSMA‐VHH. In separate 96‐well flat‐bottom plates, 5000 cells of B16‐F10 and B16‐PSMA were seeded. After 24 h, the culture medium was changed for fresh culture medium containing 10% nonheat‐inactivated FCS. Initially, cells underwent pre‐treatment with various concentrations of free VHH prior to the addition of LNPs. The cells were subjected to 5‐min incubations with 15, 1.5, or 0 nmols of unconjugated anti‐PSMA VHH. These amounts were chosen because they represent 1000x (15 nmols) and 100x (1.5 nmols), referring these to the hypothetical amounts of VHH present in anti‐PSMA LNPs encapsulating 200 ng of mRNA if total conjugation is assumed. So, as an example, the 1000x condition would mean that there is 1000‐fold more free unconjugated VHH than the amount of conjugated VHH expected onto the surface of anti‐PSMA LNPs encapsulating 200 ng of mRNA (assuming 100% conjugation). The cells were then treated with anti‐PSMA LNPs and R2‐LNPs at 200 ng of Cy5‐labeled EGFP encapsulated mRNA per well. The cells were then incubated for 24 h at 37 °C and 5% CO_2_. The cells were then washed twice with DPBS, trypsinized and resuspended in 250 µL DPBS 2% FBS. The well content from all the wells in the plate was moved into 96 U‐bottom well plates (Greiner), where uptake (Cy5) and functional transfection (EGFP) were analyzed on a FACS Canto II flow cytometer (Becton, Dickinson and Company). Results are shown as a proportion of the Cy5 (APC‐A channel) and EGFP (FITC‐A channel) MFI signal for each of the LNP treatments in respect to their paired conditions without pre‐treatment of unconjugated anti‐PSMA‐VHH. This approach allows for the comparison of the treatment effects by controlling for any potential influence of the unconjugated VHH pre‐treatment. The measured MFIs for Cy5/EGFP were corrected by subtracting the MFIs obtained for PBS treated cells. Data represent mean ± SD (*n* = 3 wells) with at least 5000 cells per well.

### Animal Experiments—Zebrafish Housing and Maintenance

In vivo experiments have been performed in zebrafish (*Danio rerio*) Casper embryos from 0 to 4 days post fertilization (dpf), in accordance with European Animal Welfare Legislation “*on the protection of animals used for scientific purposes*”, following the 3Rs principles (Convention ETS – No. 123, Directive No. *2010/63/EU*) and INSERM U1266‐IPNP internal Zebrafish Facility rules. The study and all the procedures were approved by CEEA Ethical Committee and French institutional organizations (agreement No. C75‐14‐03). In vivo experiments have been performed in zebrafish (*Danio rerio*) Casper embryos from 0 to 4 days post fertilization (dpf), in accordance with European Animal Welfare Legislation “*on the protection of animals used for scientific purposes*”, following the 3Rs principles (Convention ETS – No. 123, Directive No. *2010/63/EU*) and INSERM U1266‐IPNP internal Zebrafish Facility rules. The study and all the procedures were approved by CEEA Ethical Committee and French institutional organizations (agreement No. C75‐14‐03). All the animals were staged and cared according to standard protocols^[^ ^68^ ^]^ and FELASA recommendations.^[^ ^69^ ^]^ Adults were maintained at a density of ≈7–8 fishes/L per tank in reverse osmosis‐filtered water at 28 °C, with a daily photoperiod of 14 h light/10 h dark. Fishes were fed twice per day with artemia (brine shrimp)‐based dry food (75, 150, 300, or 400 µm‐granules, depending on the developmental stage – DIETEX France). Matings were made in groups of maximum 12 fishes (at sex ratio of 1 male: 2 females or 1 male: 3 females, to reduce the frequency of aggression events^[^ ^70^ ^]^) into polycarbonate autoclavable 1.7 L‐sloped mating systems filled with aquarium water at 28 °C. Males and females were kept physically separated overnight, to increase sexual libido. The next morning (08:30 a.m. up to 11:30 a.m.) the divider was taken away, leading to eggs and sperm release and further fertilization.^[^ ^71^ ^]^ Zebrafish embryos were collected in Egg water‐medium into 10 cm‐Petri dishes, and kept at 28 °C in incubators with photoperiod, to maintain their natural circadian rhythm (14 h light/10 h dark per day), until 6 dpf. Egg water‐medium 10X stock was made by dissolving 3.75 g of Instant Ocean Salts Mix (Aquarium Systems, Ohio, USA) and 1 g of Calcium sulfate dihydrate (CaO4S‐2H2O, Sigma‐Aldrich) in 1.25 L reverse osmosis‐filtered water. The working solution was 1X Egg water‐medium diluted in reverse osmosis‐filtered water. No harmful phenotype was observed during experiments. Fishes’ health and housing conditions were evaluated periodically through sanitary controls headed by a veterinary doctor.

### Animal Experiments—LNCaP Cells Xenografts into the Perivitelline Space (PVS) of Zebrafish Embryos

At 2 dpf, embryos chorions were manually removed one‐by‐one under the stereoscope using sharp forceps, or enzymatically dechorionated in bulk by incubation in Egg‐water supplemented with 2 mg mL^−1^ Pronase endo‐exoproteases mix (Roche) at room temperature for a brief time (1–10 min). The turgidity of the chorions was checked constantly by gently depressing them with a poker: when chorions no longer returned to a spherical shape, the Pronase treatment was considered complete. In parallel, LNCaP cells to transplant were brought at 90% confluence in adhesion into a T‐75 flask and pre‐incubated with 200 nm MemBright‐488 lipid dye diluted in serum‐free RPMI for 30 min at 37 °C. After two washes in 1X PBS and trypsin‐mediated detachment, labelled cells were pelleted at 300 g for 5 min at 4 °C. The pellet was then resuspended in 1X PBS and another step of centrifugation at 300 g for 5 min at 4 °C was done to remove further debris. The cell pellet was then resuspended in sterile 1X PBS into a 1.5 mL tube reaching a concentration of ≈0.25–0.5×10^6^ cells µL^−1^, put in an ice bucket and brought into the zebrafish facility. Simultaneously, dechorionated zebrafish embryos at 2 dpf were anaesthetized into a 10 cm‐Petri dish with 1X Tricaine in Egg water‐medium for 5 min. After anesthesia, embryos were aligned in open air onto a 2% agarose plate bed using a homemade hairpin loop, and always ensuring that embryos didn't dry by adding a film of 1X Tricaine in Egg‐water during the whole microsurgery procedure. Only well‐developed embryos, were selected for xenografts. Borosilicate capillary tips (Harvard Apparatus, Massachusetts, USA) using a Sutter Instrument Model P‐30 vertical micropipette puller (heat = 800; pull = 300) were pulled. At this point, LNCaP cells were resuspended by gentle tapping the microcentrifuge tube, aspirated (≈10 µL of suspension) and back‐loaded into a borosilicate capillary using a micro‐loader tip (Eppendorf Corporate, Hamburg, Germany). Once filled with cells, the borosilicate capillary was mounted into a Narishige GJ‐8 Magnetic Stand with micromanipulator (Science Products GmbH, Hofheim am Taunus, Germany), and oriented with the tip toward the PVS. The tip of the capillary was then cut with a blunt end using sharp forceps. For xenograft microinjection, SYS‐PV820 Pneumatic PicoPump (World Precision Instruments, Florida, USA) was used set as follows: Hold pressure = vent (3 psi); Eject pressure = vent; Range = 100 ms. After carefully piercing in the middle of the embryo's yolk, lowering the angle of the needle and cautiously pushing until the tip of the needle reached the PVS, cells were injected by pressing the microinjector pedal, forming a solid mass located as far as possible from the heart, and with a diameter similar to the embryo's eye as reference (≈10 nL in volume, so ≈200–400 cells). In case of cells clogging, the pressure was slightly increased and/or the capillary tip further cut. After injection, embryos with the xenografts were first kept anaesthetized for 5–10 min in a new 10 cm‐Petri dish within 1X Tricaine‐Egg water medium and after they were shifted into fresh Egg‐water medium and incubated at 34 °C overnight. The first day post injection (dpi) [3 dpf] LNCaP xenografts were observed under a fluorescence microscope, and the ones containing few cells, presenting cardiac oedema or with cells inside the yolk were discarded. All the embryos selected for further experiments presented LNCaP metastases in the caudal vein plexus (CVP) in the tail at 3 dpf.

### Animal Experiments—LNPs Intravenous Injection in the Duct of Cuvier of Zebrafish Embryos

At 3 dpf, LNCaP‐xenografted zebrafish embryos were first aligned onto a 35 mm Ibidi high µ‐Dish with optical transparent polymer coverslip bottom (Ibidi GmbH, Gräfelfing, Germany) and embedded in 0.5% low‐melting agarose (Condalab, Madrid, Spain) dissolved in Egg‐water medium, and supplemented with 164 mg L^−1^ Tricaine (MS‐222). After 10–15 min, once the agarose solidified, a suspension of 0.2% DSPE Rhodamine‐labelled LNPs bearing BFP mRNA in sterile 1X PBS was mixed 1:10 v/v with 0.5% water dissolved Phenol red solution (Merck) and charged into a borosilicate capillary using a microloader tip (Eppendorf Corporate). The borosilicate capillary was then mounted into a Narishige GJ‐8 Magnetic Stand with micromanipulator (Science Products, GmbH), and oriented with the tip toward the *Duct of Cuvier* of the zebrafish embryo. The tip of the capillary was then cut to obtain a piercing, oblique‐angled end. Once reached the *Duct of Cuvier*, a volume of 2 nL of LNPs is microinjected, corresponding to a final dosage of ≈1 mg kg^1^ mRNA. After LNPs microinjection, the embryos were de‐embedded and put back into 10 cm Petri dish filled with Egg‐water at 34 °C. After 7 hpi, the zebrafish embryos were fixed in 4% PFA in Egg‐water, aligned, and mounted between two 24 mm x 50 mm Thorlabs' Precision Cover Glasses with Mowiol 4–88 aqueous mounting medium, securing the borders with Vaseline. Images of the embryos were acquired at Leica SP5 inverted confocal microscope STED CW equipped with three photomultiplier modules (PMT) with 20x oil immersion objective and processed on Leica LAS AF Lite 4.0 software.

### Ethical Statement on Mice Experiments

All animal experiments were conducted in accordance with the ethical standards and regulations set forth by the Utrecht Animal Welfare Body and complied with the Dutch Experiments on Animals Act (WOD) license AVD10800202115026. The experiments adhered to the guidelines outlined in the “Guide for the Care and Use of Laboratory Animals”. The animals had continuous access to water and standard chow ad libitum and were kept in a consistent environment with a 12‐h light/dark cycle. The animals were randomly assigned to different treatment groups and cages, and blinding of the operators was carried out when possible.

### Biodistribution and Functional Delivery in B16‐F10‐PSMA Engrafted Mice

After a 7‐day acclimatization period, male C57BL/6NCrl mice (Charles River Laboratories, 20−28 g, 7−10 weeks) 5×10^6^ B16‐PSMA cells in 50 µL unsupplemented medium were subcutaneously injected in the right flank of the animals. Tumor growth was monitored until it reached 300 mm^3^, when treatments were administered. For treatments, mice received intravenous (i.v.) LNP injections in the tail vein of 0.2% anti‐PSMA LNPs or 0.2% R2‐LNPs, labeled with DSPE‐Cy5 and encapsulating firefly luciferase mRNA (0.5 mg mRNA kg^−1^ animal). Twenty‐four hours post‐injection, live bioluminescence was performed on all animals. The animals were anaesthetized with 4% isoflurane, the hair around their tumors was shaved with an electric razor, and they were injected intraperitoneally with 2.5 mg D‐luciferin (Promega, Wisconsin, USA) in a total volume of 100 µL. After 10 min, the luminescence in live animals was evaluated with an IVIS RT PhotonImager (BioSpace Lab, Nesles‐la‐Vallée, France) and quantified using the M3 Vision Software (BioSpace Lab). After live imaging, the animals were then terminated by cervical dislocation and their organs were collected, washed in a DPBS bath, and analyzed for luciferase signal in the IVIS RT PhotonImager. The tumors were split in two: one half was put in a 15‐mL flask containing 5 mL cold DPBS, for flow cytometry analysis to find out in which tumor cell types there was uptake of LNPs; the other half of the tumor and the selected organs were snap‐frozen in liquid nitrogen and stored at −80 °C for tissue lysate analysis and histology. The tissues collected included tumor, liver, spleen, heart, lungs, and kidneys.

### Tissue Lysates Preparation for Fluorescence and Luminescence Analysis

Tissue lysates were prepared from tumor, liver, spleen, lungs, kidneys, and heart. Tissues were thawed and weighed ≈100 mg of each organ was transferred to reinforced 2 mL Bead Mill tubes (VWR International, Pennsylvania, USA) containing stainless steel beads (2.8 and 5 mm) (Qiagen, Hilden, Germany). Five microliters of 1X Cell Culture Lysis Reagent (Promega) supplemented with a 100X diluted Protease/Phosphatase inhibitor cocktail (Cell Signaling Technology, Massachusetts, USA) were added per milligram of tissue. Homogenization was carried out using a Mini bead‐beater (Bertin Technologies, Montigny‐le‐Bretonneux, France) for 60 s at 5000 rpm. After centrifugation for 10 min at 10 000 g and 4 °C, the supernatant was transferred to a new tube. For Cy5 measurement, 50 µL of the supernatant was transferred into a black 96‐well plates where fluorescence was measured using a Spectramax ID3 instrument (Molecular Devices, California, USA) at 640/685 nm for excitation/emission wavelengths, respectively. Luciferase enzymatic activity was assessed using a Spectramax ID3 equipped with an injector (Molecular Devices). Ten microliters of supernatant were placed into a white 96‐well plate (Greiner), followed by injector‐mediated dispensing of 50 µL of Luciferase assay reagent (Promega) to each well. The reaction mixture was then incubated for 2 s before luminescence was measured and integrated over a period of 10 s.

### Tumor and Liver Cryosectioning, Imaging, and Analysis

Eight micrometer liver and tumor cryosections were cut using a SLEE MNT cryostate (SLEE medical GmbH, Nieder‐Olm, Germany). After cutting, the slides were stained and cover slipped manually by using DAPI Vectashield HardSet Antifade Mounting Medium (Vector Laboratories, California, USA), and H&E staining was performed and cover slipped using a Clearvue™ coverslip (Thermo Fisher Scientific). Imaging of the H&E slides was performed using a Hamamatsu XR (Hamamatsu, Japan) and the slides were analyzed on necrosis percentage by expert pathologists from the UMC Utrecht (TNG, RGO). Regarding the imaging and quantification of Cy5 signal in the tumors (LNP uptake), 15 images of each of 2 distal tumor cryosections from every tumor were captured at 20x magnification using a Zeiss Axio Observer Z1 Microscope equipped with an AxioCAM MRm (Carl Zeiss AG, Oberkochen, Germany). Random areas of the tumors were focused using the DAPI channel, and images were taken from different channels upon the excitation of their respective lasers; namely: DAPI (nuclei of the cells), Cy5 (LNP uptake), and Cy3 (autofluorescence). The same laser intensities and visualization settings were applied to process and extract the images. Images were analyzed using a custom‐made script in ImageJ. In brief, Cy3 channel images depicting autofluorescent spots were thresholded based on intensity. Next, the pixels containing Cy3 signal were removed from the Cy5 channel image, after which the total intensity and area of Cy5 was quantified per image.

### Tumor Sample Preparation and Analysis by Flow Cytometry

Mice‐derived tumors were preserved in 5 mL DPBS on ice and then moved to Petri dishes. Afterward, 2.5 mL of tumor digestion buffer was added, and the tumor tissue was finely chopped with surgical blades. A stock of 100 mL of Tumor digestion buffer consisted of 120 µL DNAse I, grade II (Merck) (1 mg mL^−1^), 33 mL collagenase type 3 (Cell Signaling Technology) (10 mg mL^−1^), and 87 mL DMEM. The minced tissue was then kept at 37 °C for 60 min. Following this incubation period, the mixture was transferred to a 15 mL tube and vigorously pipetted up and down to break up any clumps. It was subsequently filtered through a Falcon 70 µm Cell Strainer (Corning, New York, USA), rinsed with 10 mL DMEM, and centrifuged at 500 g for 7 min at 4 °C. The supernatant was discarded, and the cells were resuspended in 2.5 mL ACK lysing buffer (Thermo Fisher Scientific). After a 5‐min resting period, 20 mL of DPBS were introduced to deactivate the lysing buffer. The cells underwent another filtration through a Falcon 70 µm Cell Strainer (Corning), followed by centrifugation and resuspension in DMEM. Cell counting was carried out using Trypan Blue solution 0.4% (Thermo Fisher Scientific) in a Luna‐ II Automated Cell Counter (Logos Biosystems, Gyeonggi‐do, South Korea), and then 1×10^6^ viable cells per well were placed in a U‐bottom 96‐well plate in duplicate. Following centrifugation and removal of the supernatant, the cells were reconstituted with 50 µL of Live/Dead staining solution for 15 min at room temperature with agitation in darkness. Antibody concentrations were separately prepared in a different set of plates, and 50 µL of the antibody mix were added to the plate containing the cells. The live/dead staining solution consisted of TruStain FcX PLUS (Biolegend) (1:250) and Zombie Aqua (Biolegend) (1:200) in DPBS. The antibody mix consisted of Brilliant Violet 711 anti‐mouse CD45 Antibody (Biolegend) (1:1000), CD31 Rat anti‐Mouse, FITC, Clone: MEC 13.3 (BD Biosciences) (1:62.5) and PE anti‐human PSMA (FOLH1) Antibody (Biolegend) (1:20) in FACS buffer. After 25 min on a plate shaker in the dark, 150 µL of FACS buffer (2% BSA in PBS, 0.2 µm filtered) was added, and the plate underwent centrifugation before the cells were washed two times with 200 µL of FACS buffer. Following this, the cells were reconstituted in 100 µL of 4% PFA in PBS and incubated at 4 °C for 20 min. After subsequent centrifugation and washing, the cells were suspended again in FACS buffer and stored covered with aluminium foil overnight at 4 °C. The following day, the cells underwent centrifugation, the supernatant was carefully removed, and the cells were resuspended in 200 µL of FACS buffer for flow cytometry analysis. Samples were acquired on a 4‐laser BD LSRFortessa X‐20 Cell Analyzer (BD Biosciences). Analysis was conducted using FlowJo software v10.9.

### Statistical Analysis

The zebrafish datasets (Figure 4D,E) were previously tested for normality using the Shapiro‐Wilk test. Outliers were evaluated and excluded only when justified by a technical error. Quantitative data are presented as mean ± standard deviation (SD). For in vitro experiments, the sample size (n) refers to the number of independent wells or technical replicates per condition (typically *n* = 3). For in vivo studies, the sample size (n) corresponds to individual embryos (zebrafish) or animals (mice), as specified in the figure legends. Statistical significance was assessed using two‐tailed unpaired Student's *t*‐tests for comparisons between two groups, and typically a two‐way ANOVA with Šídák's multiple comparisons test for comparisons involving more than two groups. To determine the statistical significance of LNP quantification in tumor sections, a nonparametric one‐way ANOVA (Kruskal–Wallis test) with Dunn's multiple comparisons was used. A *p*‐value < 0.05 was considered statistically significant. Figures include exact *p*‐values where relevant, and figure legends specify the statistical tests used, sample sizes (*n*), and data presentation formats. All statistical analyses were performed using GraphPad Prism v10 (GraphPad Software, California, USA).

## Conflict of Interest

RMS is the VP of Preclinical R&D at Nanocell Therapeutics, a company working in the nucleic acid‐lipid nanoparticle field.

## Supporting information



Supporting Information

## Data Availability

The data that support the findings of this study are available from the corresponding author upon reasonable request.
