# Supplementary material for: Nanobody‐Decorated Lipid Nanoparticles for Enhanced mRNA Delivery to Tumors In Vivo
Source: Adv Healthc Mater. 2025 Jul 4;14(24):2500605. doi: 10.1002/adhm.202500605 (PMC12447046; doi:10.1002/adhm.202500605)
Supplement: Supplementary file 1 — Supporting Information [file ADHM-14-0-s001.docx]

**Nanobody-decorated lipid nanoparticles for enhanced mRNA delivery to tumors *in vivo***

Pol Escudé Martinez de Castilla ^1^, Vincenzo Verdi ^2,3^, Willemijn de Voogt ^1^, Mariona Estapé Sentí ^1^, Arnold C. Koekman ^1^, Julian Rietveld ^1^, Sven van Kempen ^4^, Qiangbing Yang ^1^, Juliette van Merris ^1^, Guido Jenster ^5^, Martin E. van Royen ^6^, Marcel H. Fens ^7^, Sander A. A. Kooijmans ^1^, Wytske M. van Weerden ^5^, Guillaume van Niel ^2,8^, Pieter Vader ^1,9^, Raymond M. Schiffelers ^1^

^1^CDL Research, University Medical Center Utrecht, Utrecht, The Netherlands

^2^Université Paris Cité, INSERM U1266, Institut de Psychiatrie et Neurosciences de Paris, Paris, France

^3^Laboratory of Cancer Biology and Genetics, National Cancer Institute, National Institutes of Health (NIH), Bethesda, MD, United States

^4^Department of Pathology, University Medical Center Utrecht, Utrecht, The Netherlands

^5^Department of Urology, Erasmus MC, Rotterdam, The Netherlands

^6^Department of Pathology, Erasmus MC, University Medical Center Rotterdam, Rotterdam, The Netherlands

^7^Department of Pharmaceutics, Utrecht Institute for Pharmaceutical Sciences (UIPS), Utrecht University, The Netherlands

^8^Centre de Recherche en Cancérologie et Immunologie Intégrée Nantes Angers (CRCI^2^NA), Nantes Université, France

^9^Department of Experimental Cardiology, University Medical Center Utrecht, Utrecht,

The Netherlands

Corresponding author:

Raymond M. Schiffelers; CDL Research, University Medical Center Utrecht, Heidelberglaan 100, 3584 CX Utrecht, The Netherlands, Tel +31 6 37610587;

r.schiffelers@umcutrecht.nl

**Supporting information**

| **Experiment** | **Nanoparticles** | **Size**  **(d. nm)** | **PdI**  **(0-1)** | **Zeta potential (mV)** | **RNA encapsulation efficiency (%)** |
| --- | --- | --- | --- | --- | --- |
| *In vitro* transfections | Anti-PSMA 1%  Cy5-labeled EGFP mRNA | 117.3 ± 10.4 | 0.099 ± 0.016 | -4.18 ± 0.53 | 96.33 ± 0.25 |
| *In vitro* transfections | Anti-PSMA 0.2%  Cy5-labeled EGFP mRNA | 118.0 ± 10.7 | 0.093 ± 0.010 | -3.04 ± 0.34 | 97.57 ± 1.04 |
| *In vitro* transfections | R2 1%  Cy5-labeled EGFP mRNA | 112.8 ± 3.8 | 0.114 ± 0.025 | -2.15 ± 0.09 | 93.24 ± 0.89 |
| *In vitro* transfections | R2 0.2%  Cy5-labeled EGFP mRNA | 108.2 ± 3.4 | 0.116 ± 0.008 | -2.27 ±  0.45 | 93.55 ± 1.12 |
| *In vitro* transfections | DSPE-PEG 1%  Cy5-labeled EGFP mRNA | 126.9 ± 5.9 | 0.273 ± 0.015 | -1.19 ± 0.17 | 94.73 ± 2.01 |
| *In vitro* transfections | DSPE-PEG 0.2%  Cy5-labeled EGFP mRNA | 107.8 ± 1.9 | 0.156 ± 0.003 | -1.23 ±  0.61 | 96.28 ± 0.15 |
| *In vitro* transfections | Uncoated  Cy5-labeled EGFP mRNA | 104.7 ± 2.9 | 0.105 ± 0.014 | 0.91 ± 0.26 | 96.26 ± 0.66 |
| Zebrafish xenograft LNCaP | Anti-PSMA 0.2%  DSPE-Rhodamine BFP mRNA | 104.0 ± 1.4 | 0.125 ± 0.013 | -3.11 ± 0.41 | 97.77 ± 1.33 |
| Zebrafish xenograft LNCaP | R2 0.2%  DSPE-Rhodamine BFP mRNA | 116.3 ± 4.7 | 0.212 ± 0.003 | 0.36 ± 0.15 | 98.15 ± 0.56 |
| Mouse xenograft B16-F10-PSMA | Anti-PSMA 0.2%  DSPE-Cy5 luciferase mRNA | 99.9 ± 1.2 | 0.073 ± 0.016 | -2.0 ± 0.90 | 97.05 ± 1.09 |
| Mouse xenograft B16-F10-PSMA | R2 0.2%  DSPE-Cy5 luciferase mRNA | 101.1 ± 1.7 | 0.081 ± 0.015 | 1.0 ± 0.17 | 96.27 ± 0.19 |

**Table S1 | Nanoparticle characterization details from all experiments.** Size, PdI, Zeta potential and RNA encapsulation efficiency were measured for all the tested nanoparticles. The results are represented by mean ± SD (n = 3 measurements).

**
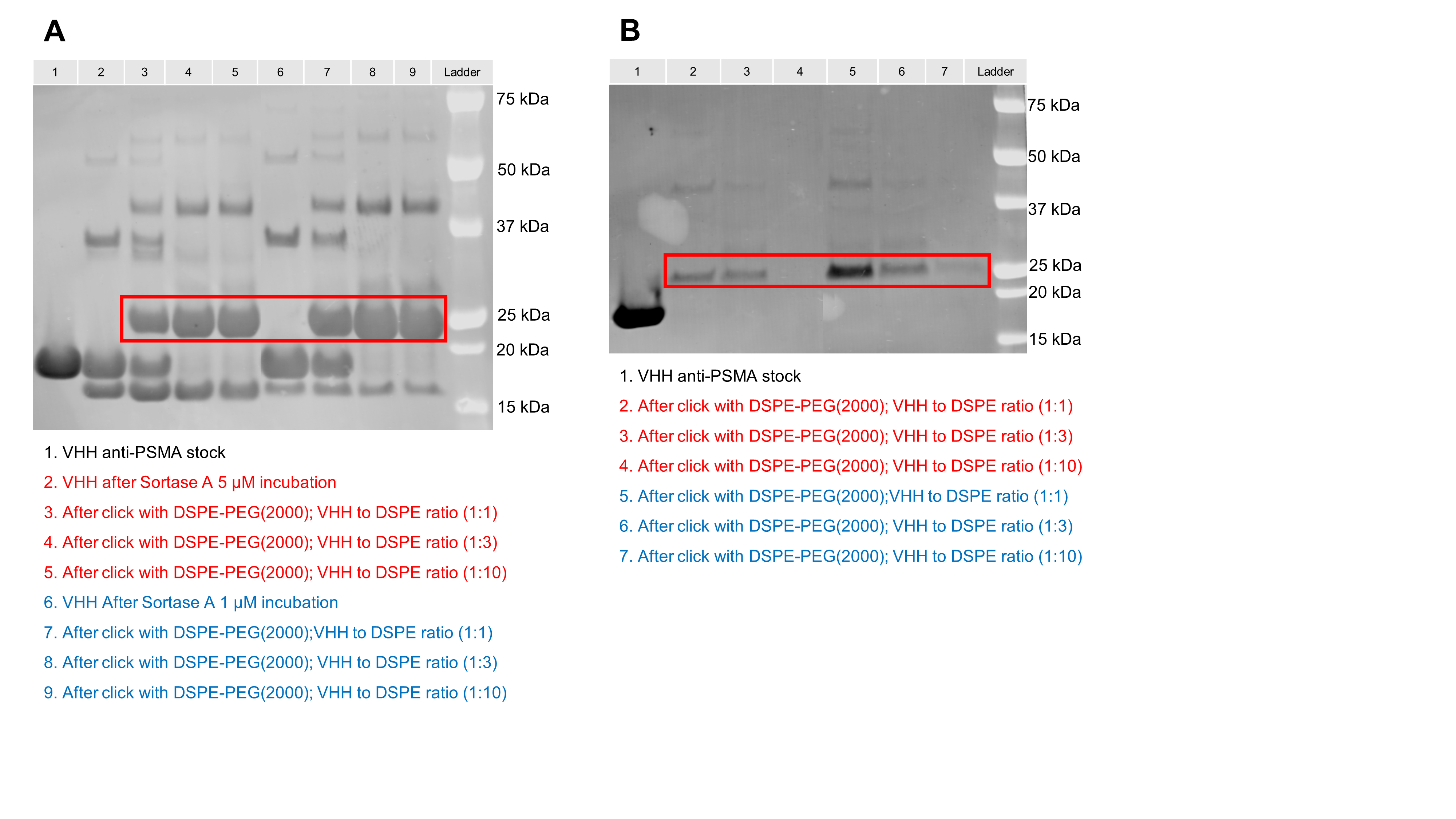
**
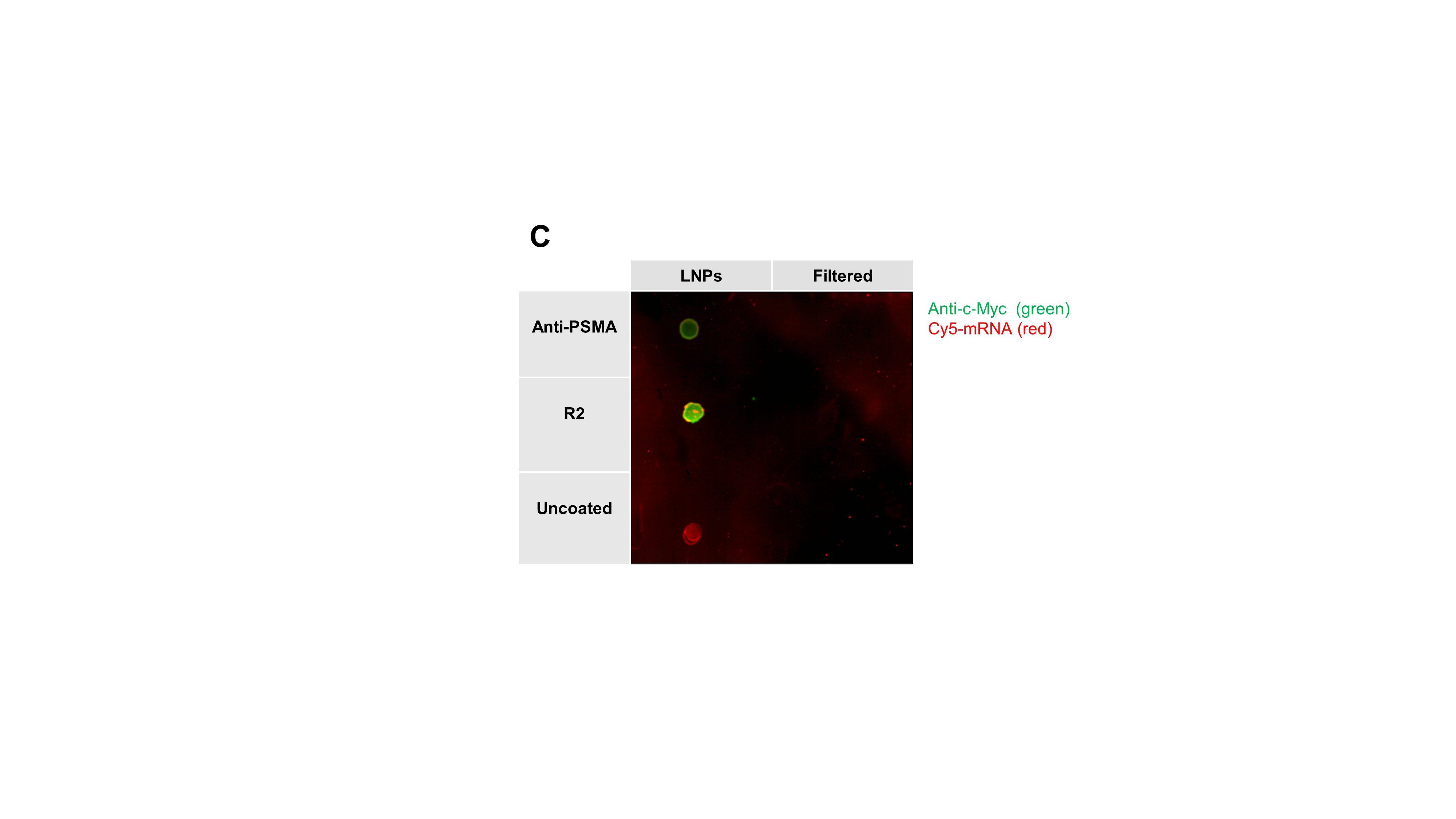


**Figure S1 |** **Validation of VHH click chemistry and post-insertion in LNPs by** **gel electrophoresis analysis and Dot Blot (DB).** Samples from various steps of VHH click chemistry **(A)** and from post-inserted LNPs **(B)** were collected, run on an electrophoresis gel, transferred to a membrane and analyzed by total protein. Marked inside a red rectangle, at around 25 kDa, the desired clicked DSPE-PEG(2000)-VHH band can be found. **(C)** We used dot blotting to detect VHHs in the final targeted-LNP solution. Targeted-LNPs were filtered through Vivaspin^TM^ 100 kDa columns and both the reconstituted non-filtered LNPs and the filtered solution were pipetted onto a nitrocellulose membrane, where they were probed with anti‐c‐Myc antibody. Dot blot results are displayed as a superposed image of 700 nm (Cy5-mRNA) and 800 nm (Anti-c-Myc) channels. Regarding the figure legends from panel A and B, conditions using 5 μM of Sortase A are denoted in red, while conditions employing 1 μM of Sortase A are denoted in blue.


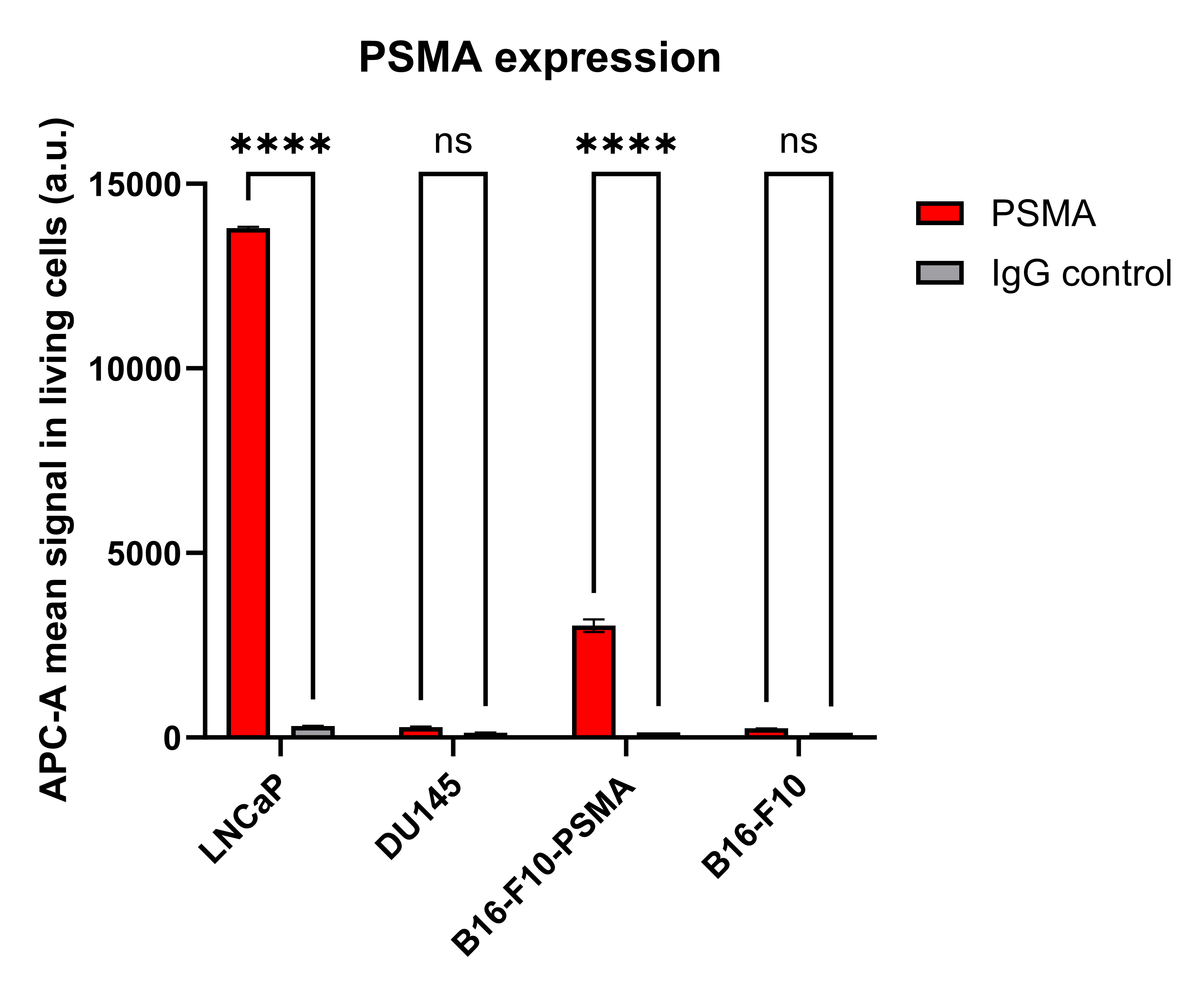


**Figure S2 | LNCaP and B16-F10-PSMA express PSMA and DU145 and B16-F10 do not.** This validation experiment ensured PSMA expression in our PSMA+ cell lines. Cells were rinsed with warm PBS to remove dead cells, then detached using a cell scraper. After centrifugation, the cell pellet was resuspended in cold PBS containing 1% sodium azide and 3% BSA. Cells were divided into two tubes, incubated with PSMA-APC antibody or IgG APC-H7 (negative control) in darkness. After incubation, cells were washed twice with cold PBS containing 1% sodium azide and 3% BSA, then analyzed for PSMA/IgG expression using the APC-A channel on a flow cytometer. A Two-Way ANOVA with Šídák's correction for multiple comparisons test was performed comparing the mean signals of each condition in between the different cell lines. ****, *p*-value <0.0001; ns: no significant difference. Data represent mean ± SD (n=3 wells) with at least 5000 cells per well.


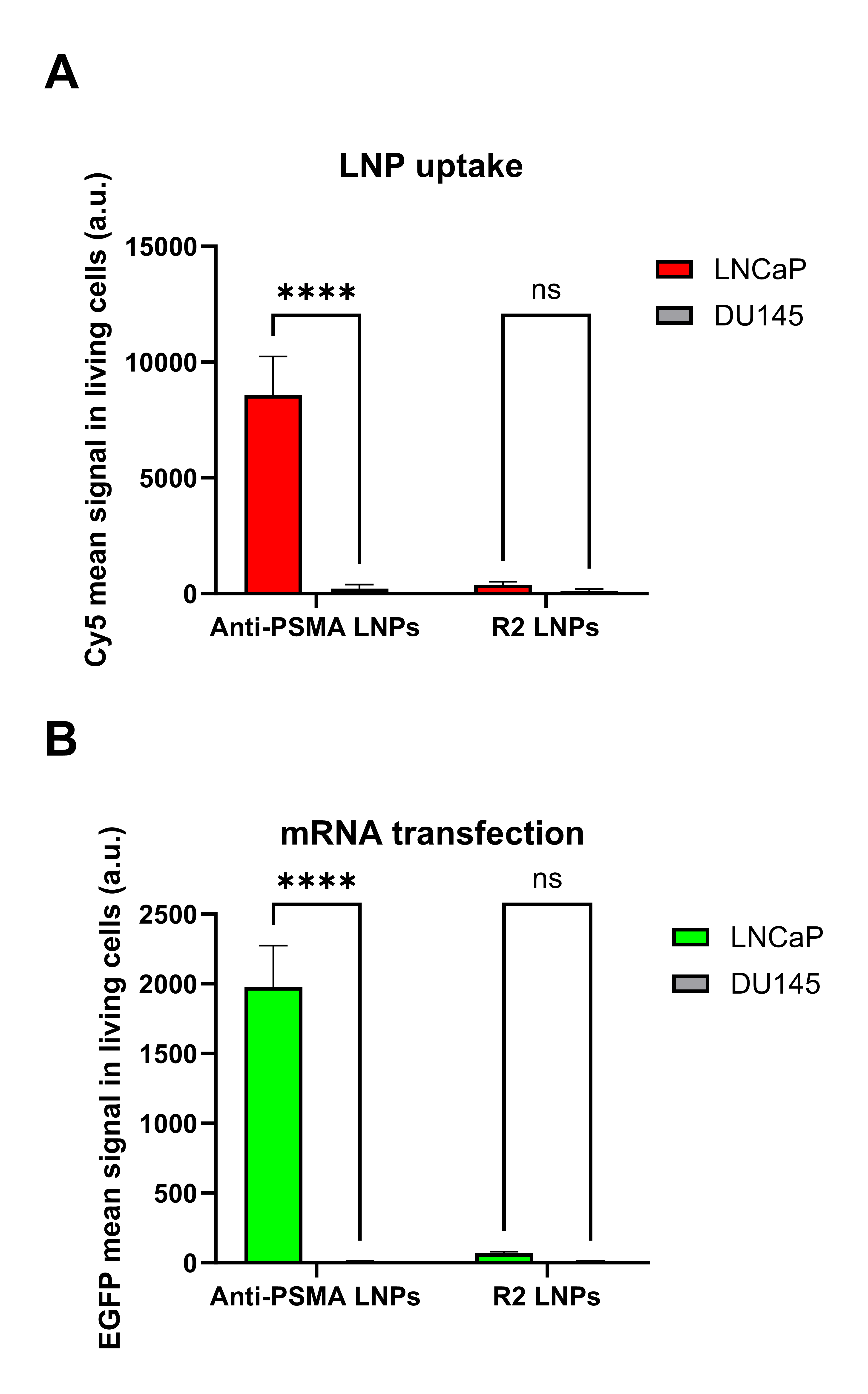


**Figure S3 | Enhanced LNP uptake and mRNA transfection of anti-PSMA LNPs in LNCaP cells.**  Flow cytometry analysis for uptake **(A)** and mRNA transfection **(B)** 24 h after addition of 200 ng of targeted-LNPs (0.2% DSPE-PEG(2000)-VHH post-inserted) encapsulating Cy5-labeled EGFP mRNA in LNCaP (PSMA^+^) and DU145 (PSMA^-^) cell lines. A Two-Way ANOVA with Šídák's correction for multiple comparisons test was performed comparing the mean signals of each condition in between the different cell lines. ****, *p*-value <0.0001; ns: no significant difference. Data represent mean ± SD (n=3 wells) with at least 5000 cells per well.


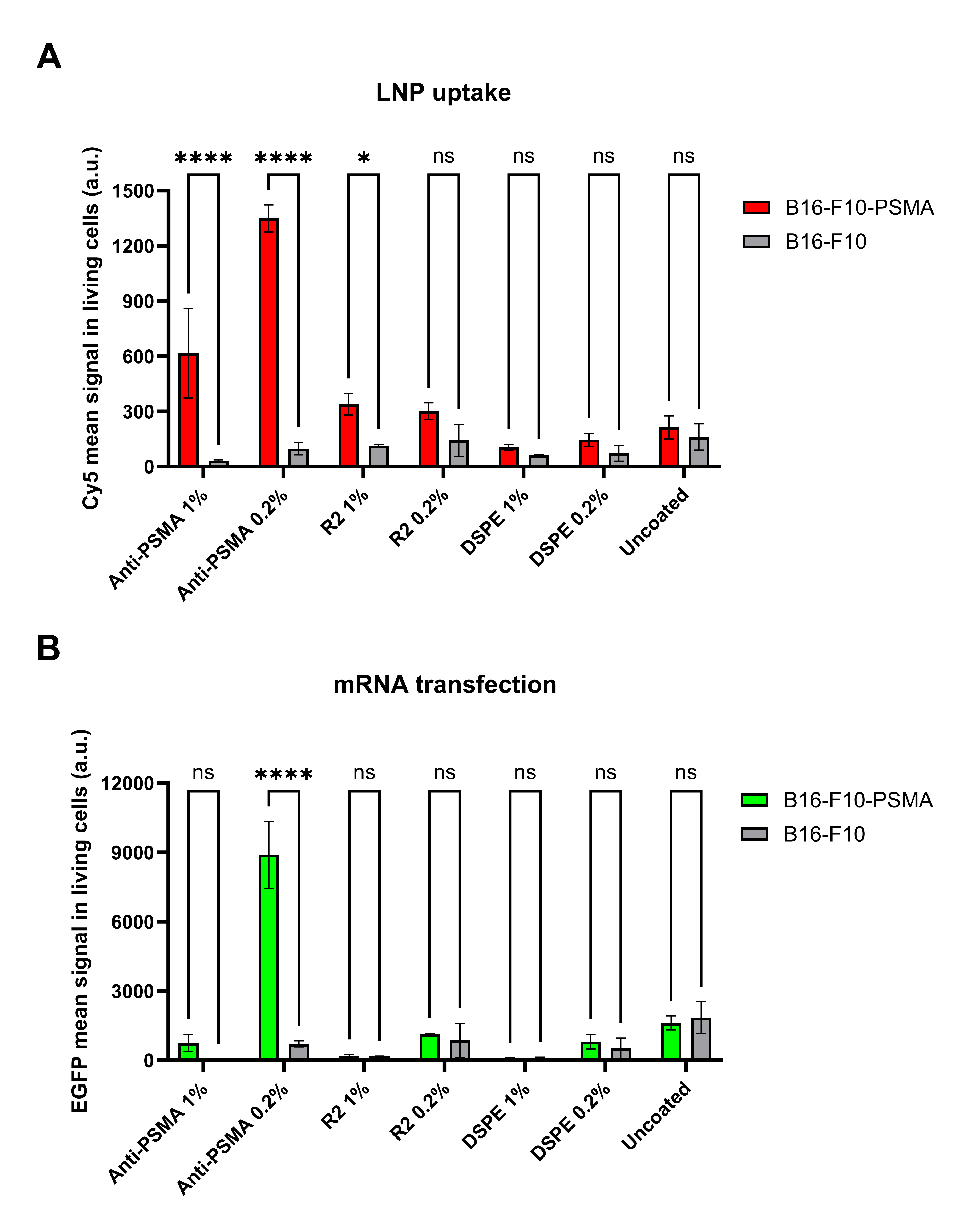


**Figure S4 | Effects on LNP uptake and mRNA transfection of varying % of post-inserted DSPE-PEG(2000)-VHH onto the surface of LNPs.**  Flow cytometry analysis for uptake **(A)** and mRNA transfection **(B)** 24 h after addition of 75 ng of targeted-LNPs encapsulating Cy5-labeled EGFP mRNA in in B16-F10-PSMA & B16-F10 cell lines. A Two-Way ANOVA with Šídák's correction for multiple comparisons test was performed comparing the mean signals of each condition in between the different cell lines. ****, *p*-value <0.0001; *, *p*-value < 0.05; ns: no significant difference. Data represent mean ± SD (n=3 wells) with at least 5000 cells per well.

**Figure S5a |** Example #1 Anti-PSMA LNPs (20x oil immersion obj. SP5)


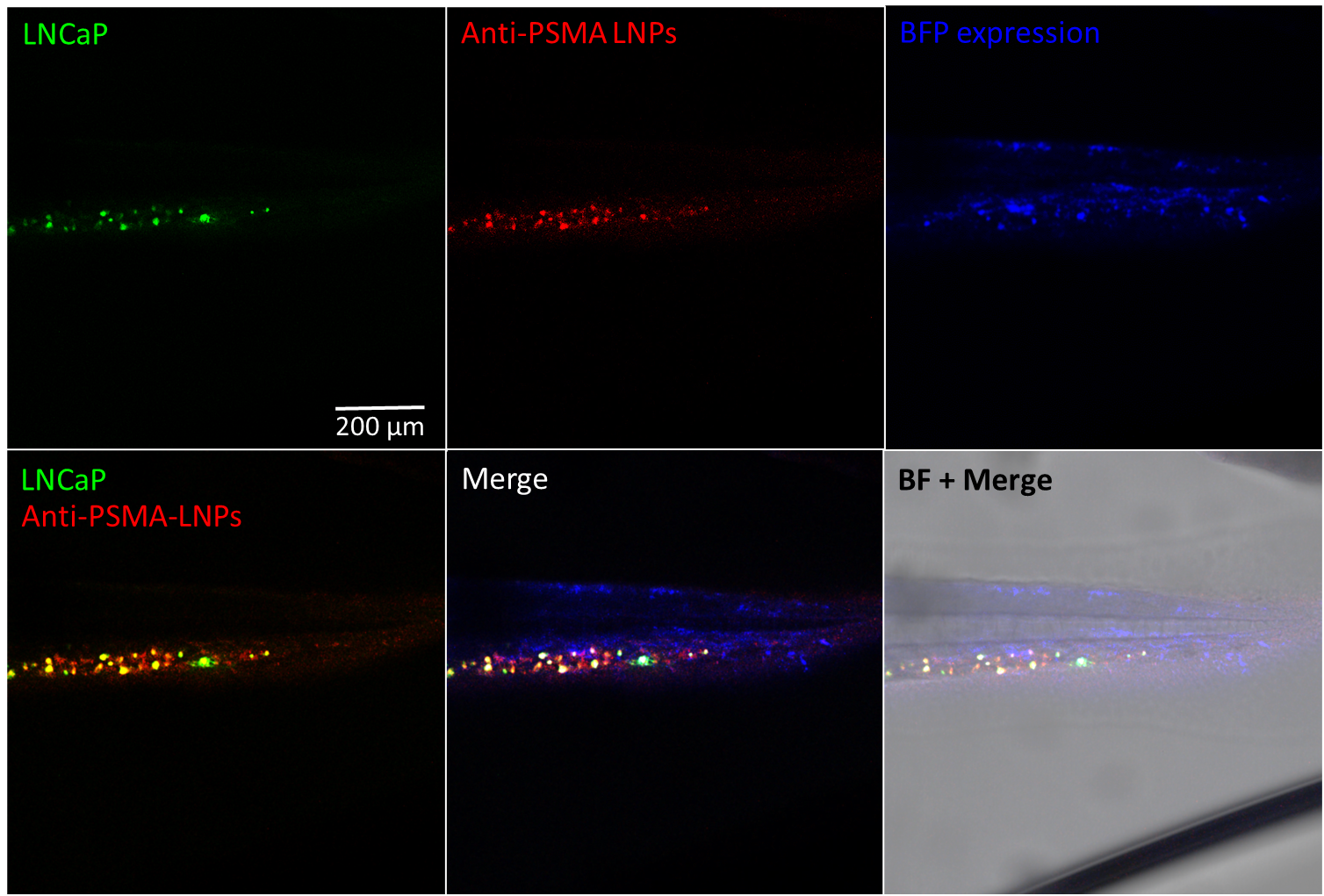


**Figure S5b** | Example #2 Anti-PSMA LNPs (20x oil immersion obj. SP5)


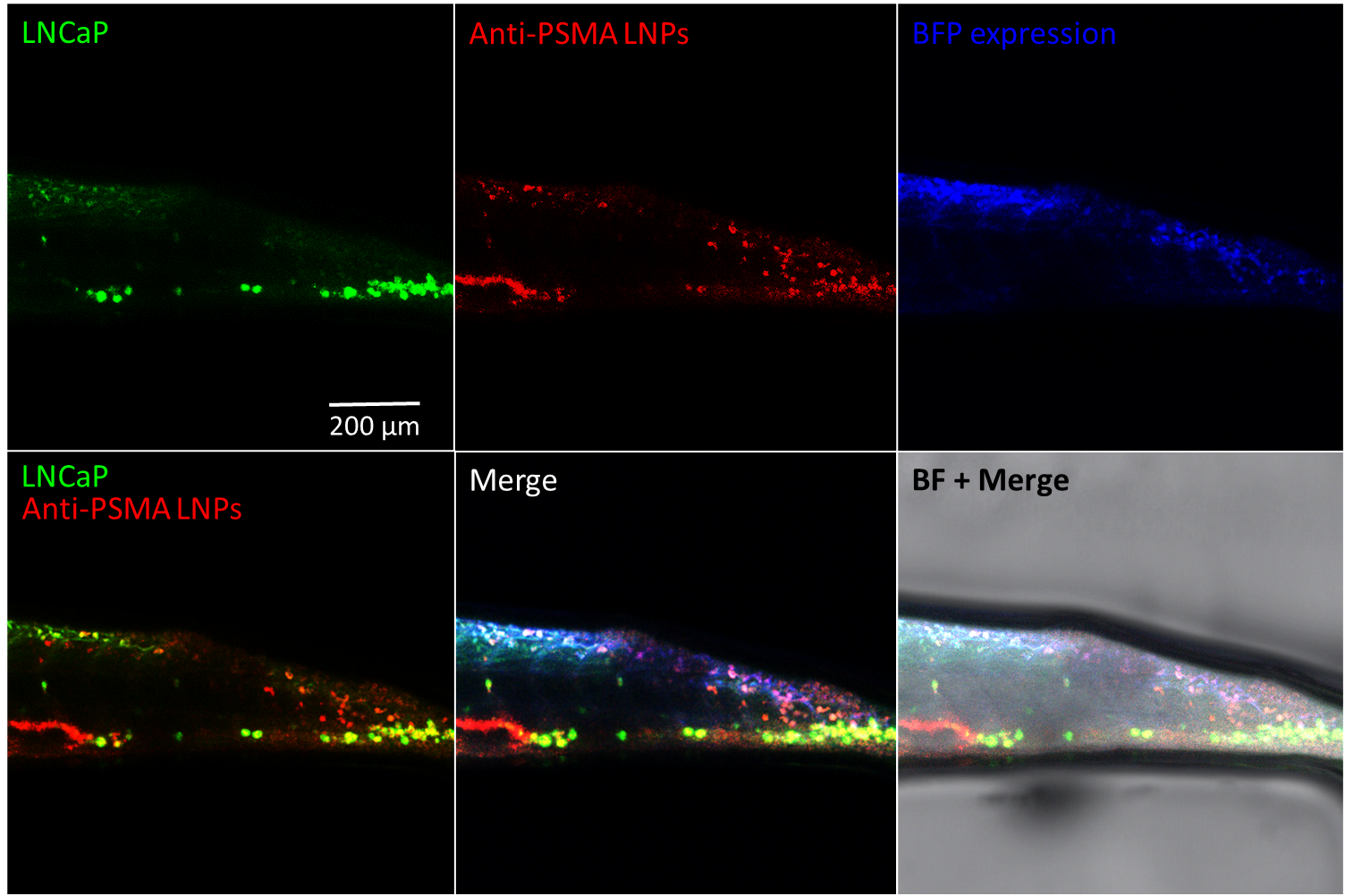


**Figure S5c |** Example #3 Anti-PSMA LNPs (20x oil immersion obj. SP5)


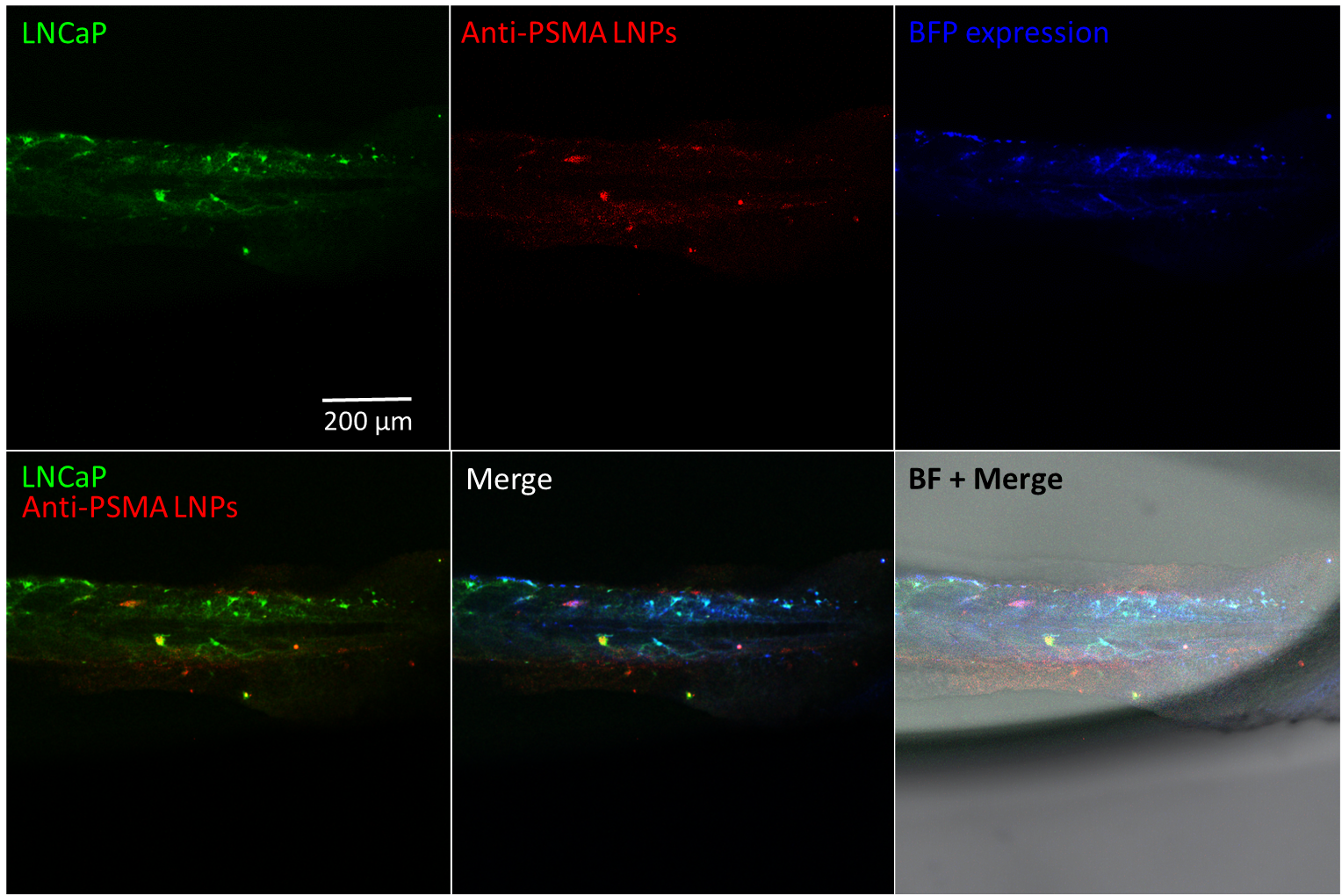


**Figure S5d |** Example #4 Anti-PSMA LNPs (20x oil immersion obj. SP5)

**
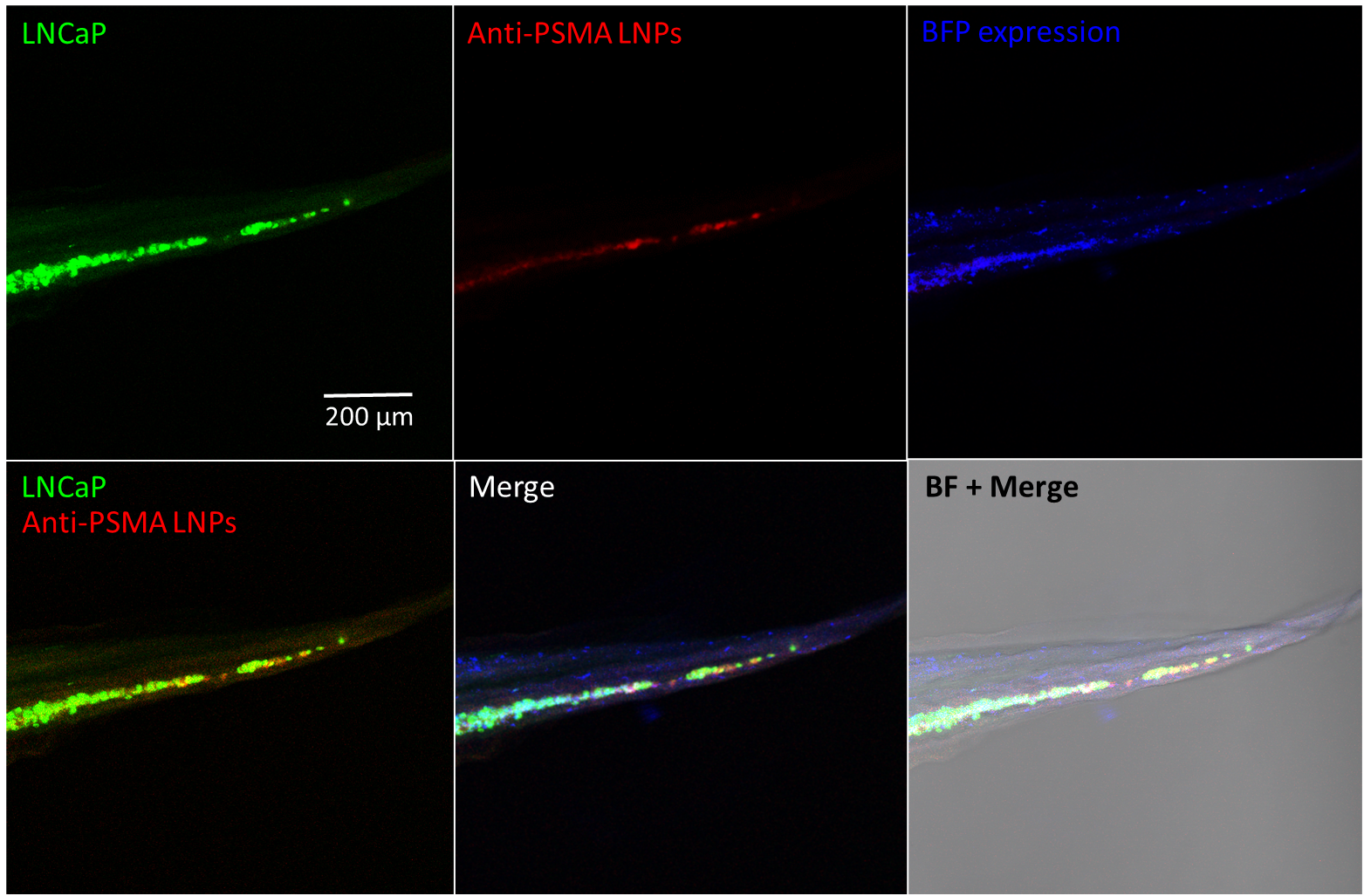
**

**Figure S5e |** Example #1 R2 LNPs (20x oil immersion obj. SP5)

**
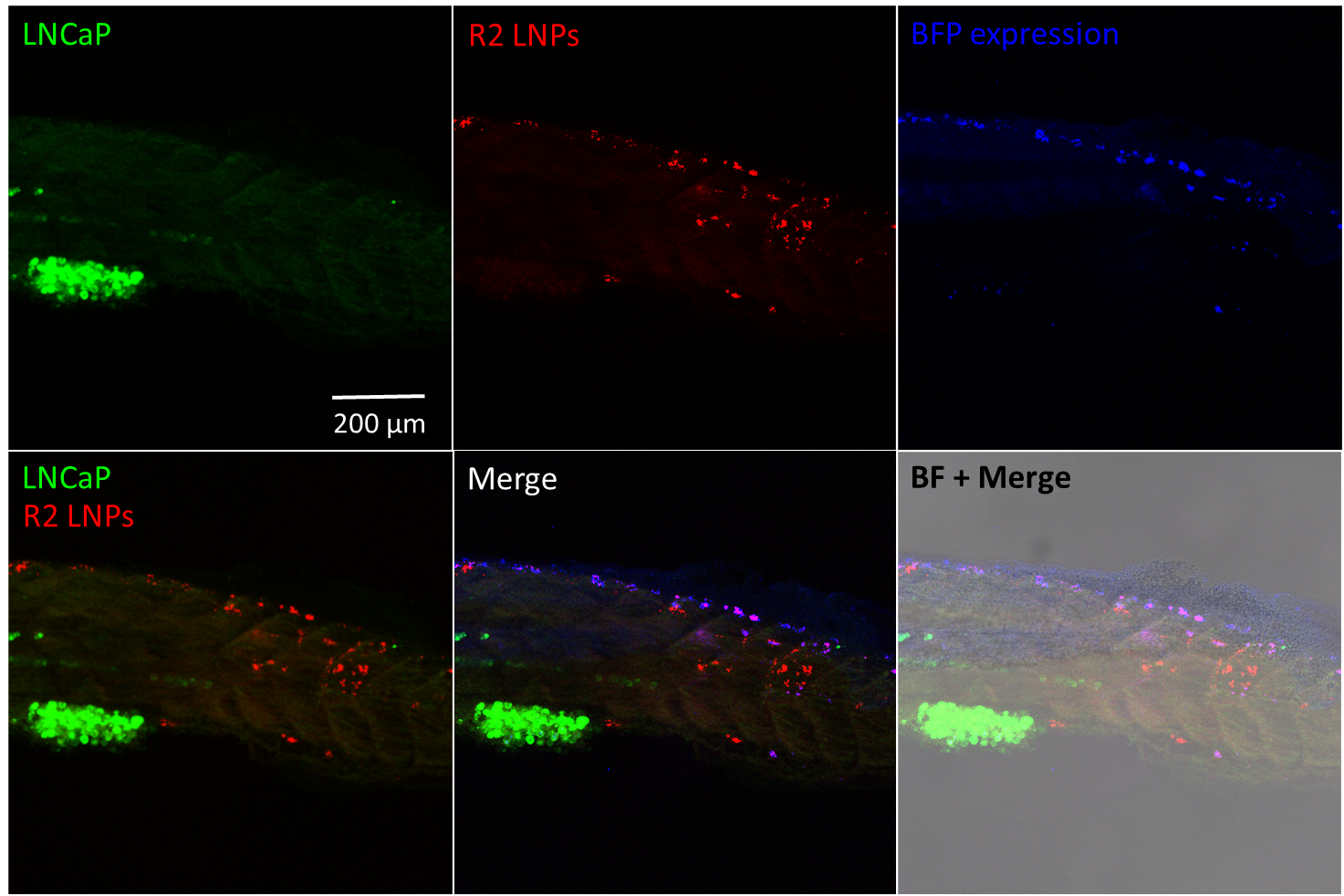
**

**Figure S5f |** Example #2 R2 LNPs (20x oil immersion obj. SP5)

**
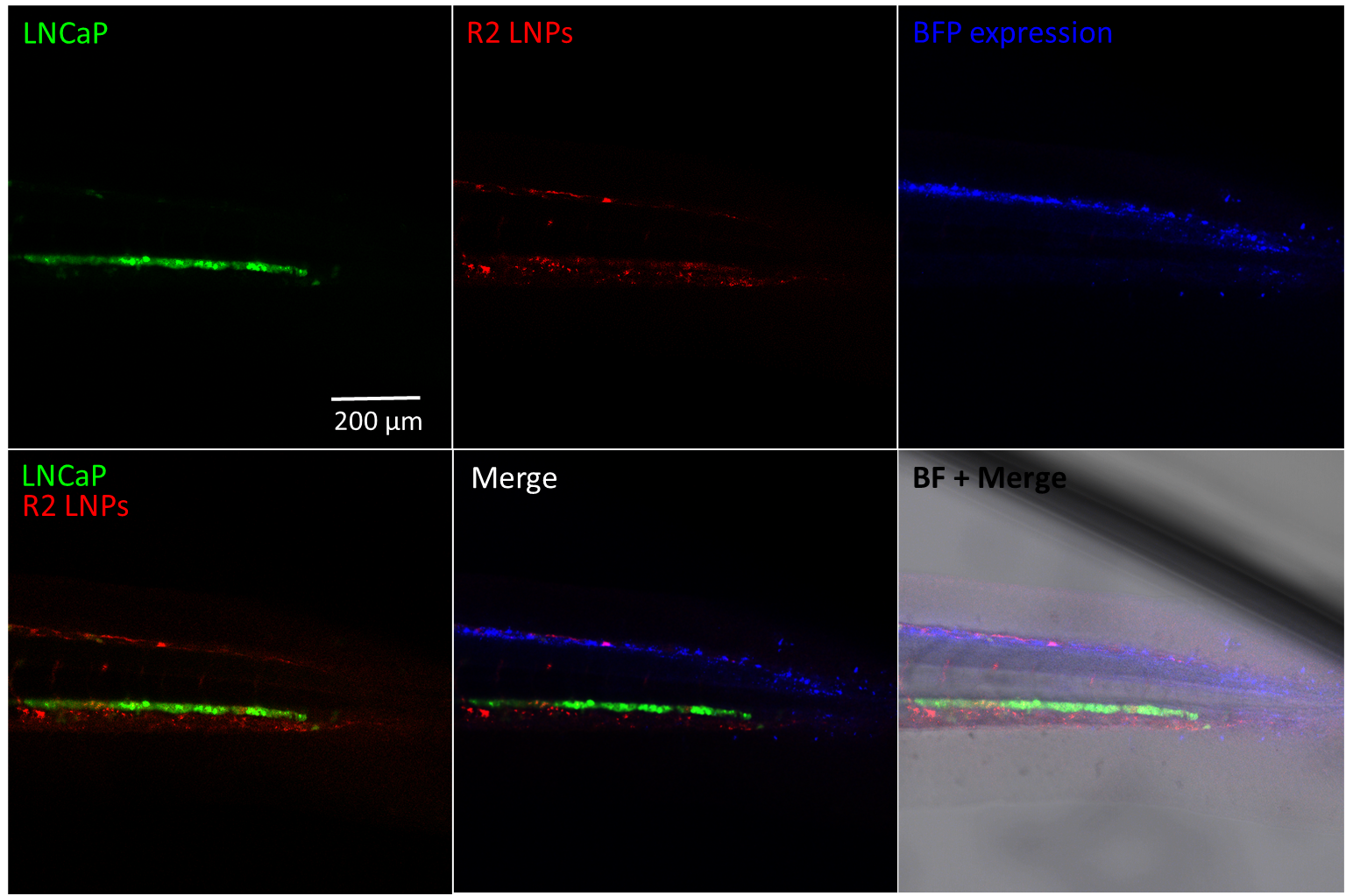
**

**Figure S5g |** Full embryo example #4 Anti-PSMA LNPs (20x oil immersion obj. SP5)

**
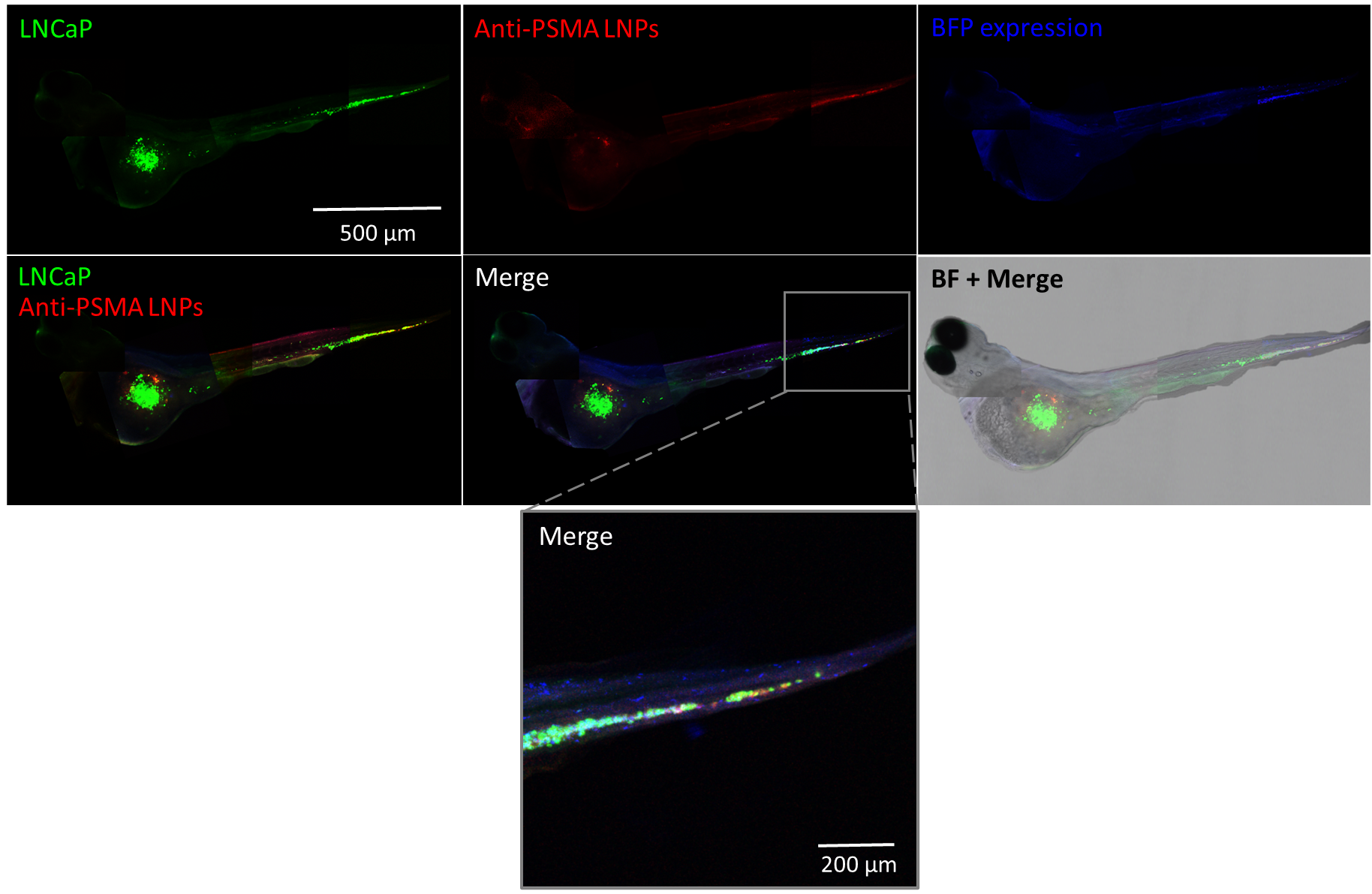
**

**Figure S5h |** Full embryo example #2 R2 LNPs (20x oil immersion obj. SP5)

**
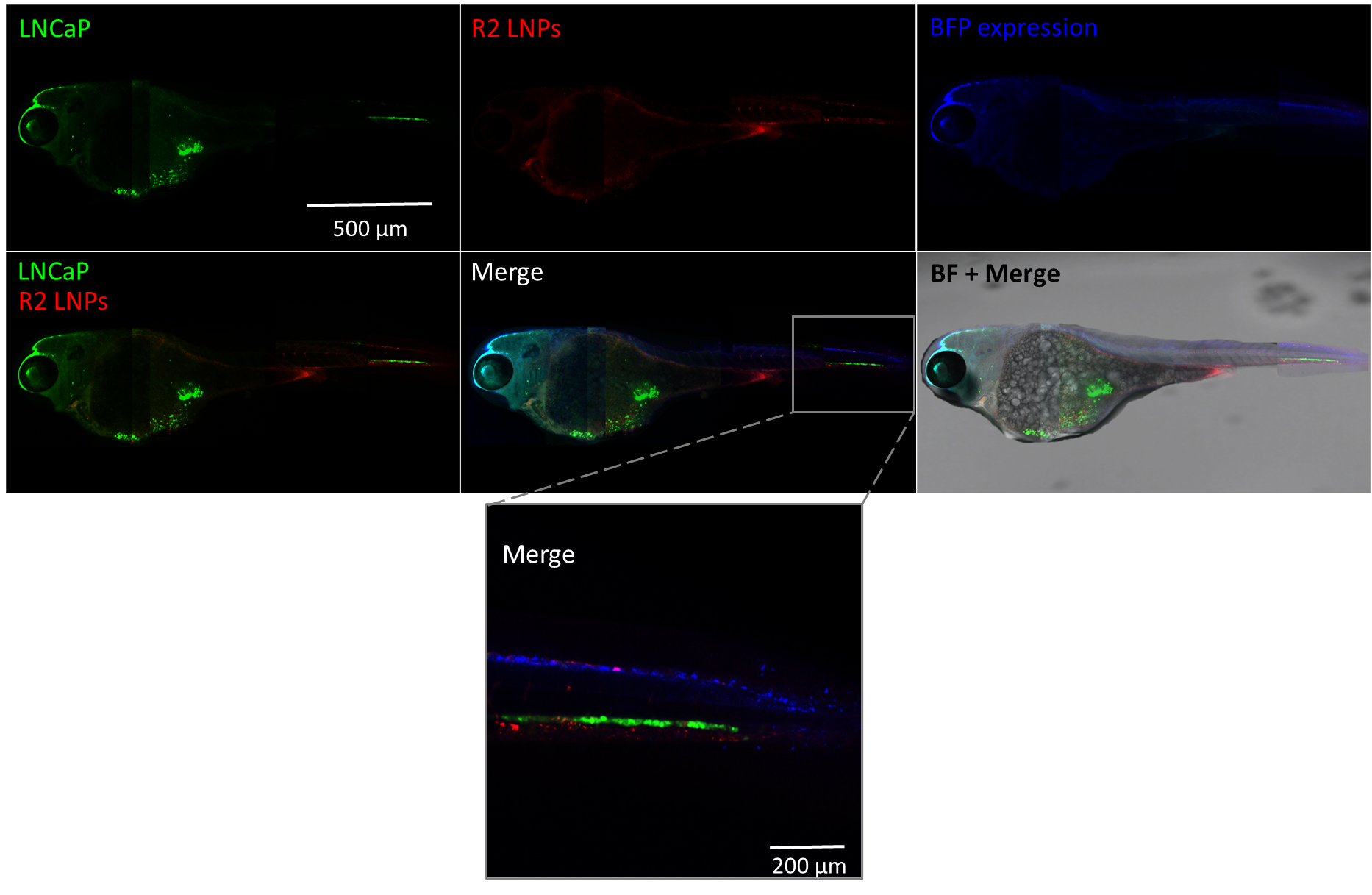
**

**Figure S5i |** Example #1 Anti-PSMA LNPs (30x silicon immersion obj. Spinning Disk)

**
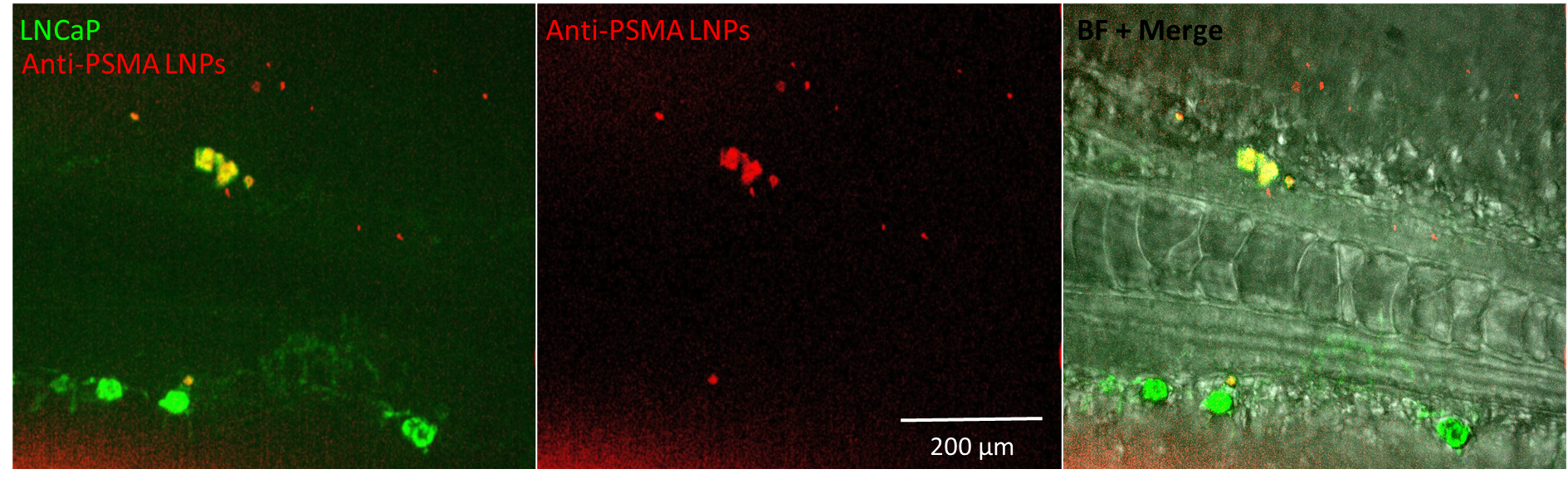
**

Targeted tumor cells

Note: Anti-PSMA LNPs target LNCaP cells infiltrating into the vasculature, but not LNCaP cells in the interstices

**Figure S5j |** Example #2 Anti-PSMA LNPs (30x silicon immersion obj. Spinning Disk)

**
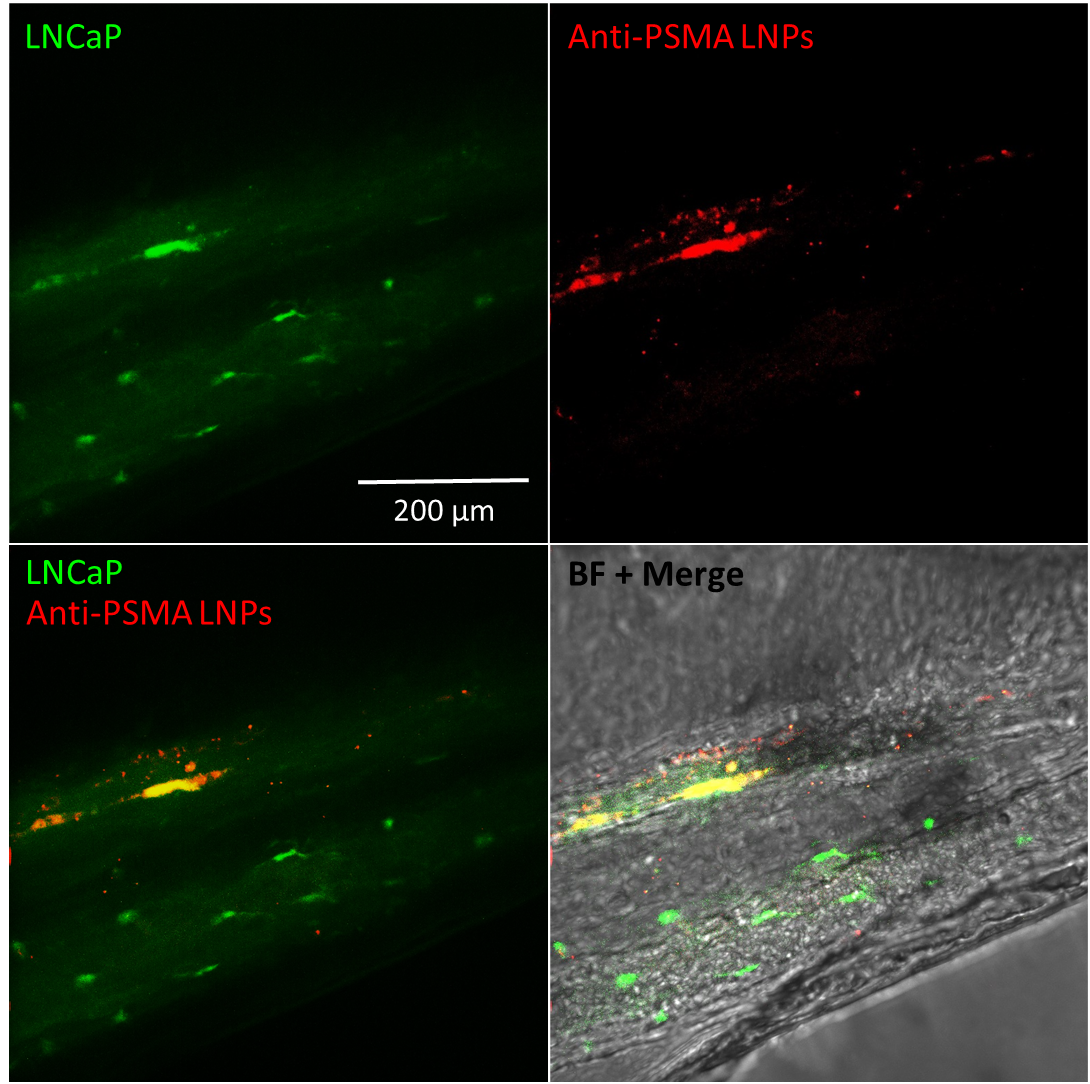
**

Targeted tumor cells

**Figure S5k |** Example #3 Anti-PSMA LNPs (30x silicon immersion obj. Spinning Disk)

**
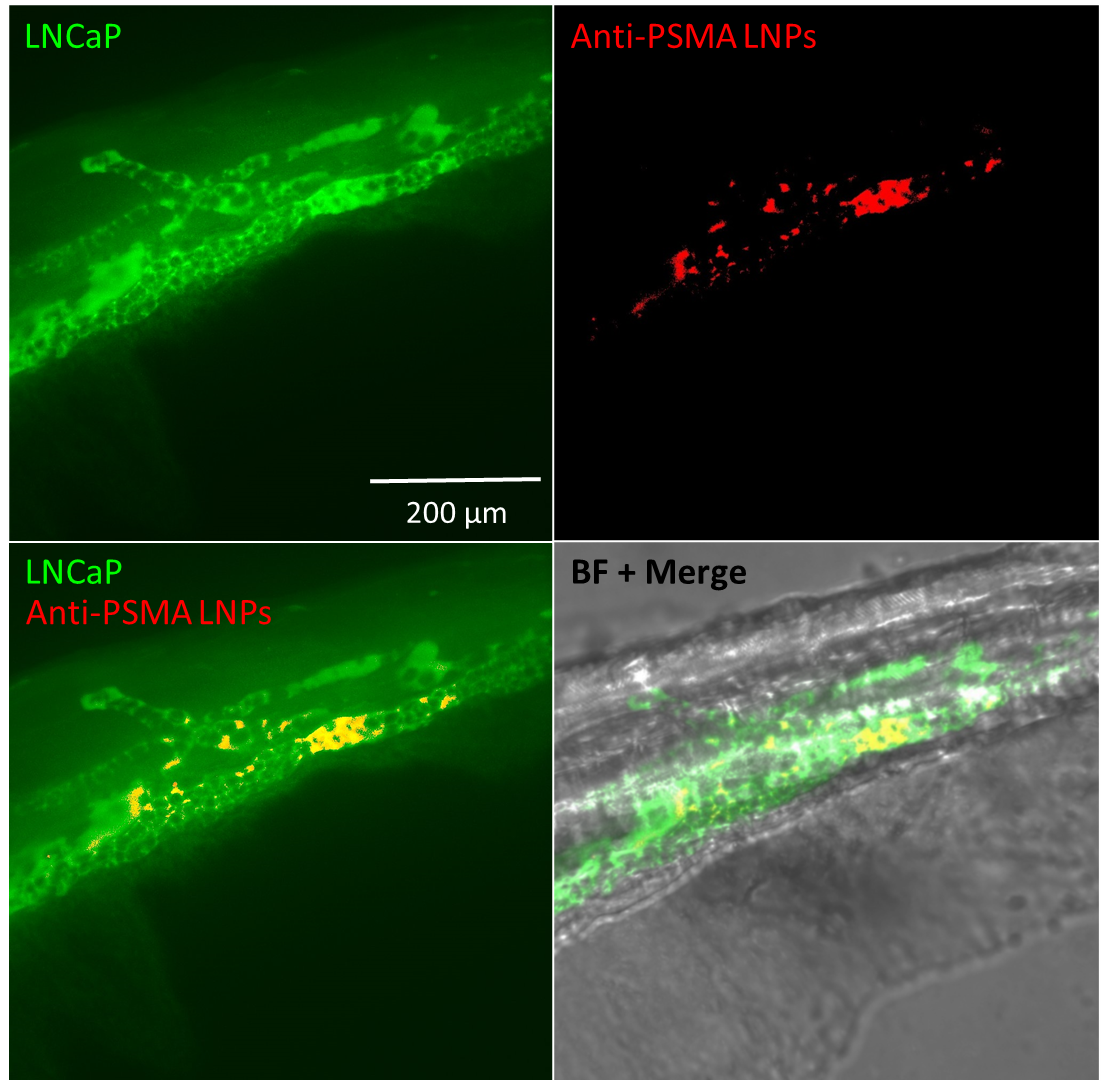
**

**Figure S5l |** Example #1 R2 LNPs (30x silicon immersion obj. Spinning Disk)

**
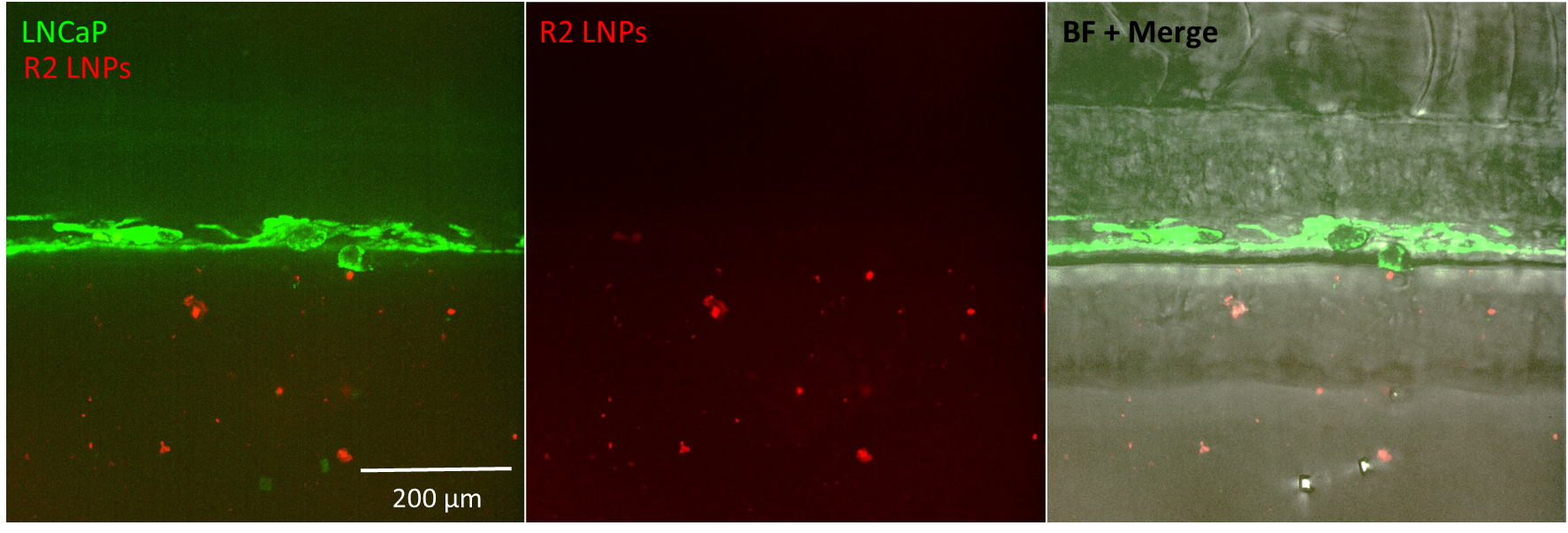
**

**Figure S5m |** Example #2 R2 LNPs (30x silicon immersion obj. Spinning Disk)

**
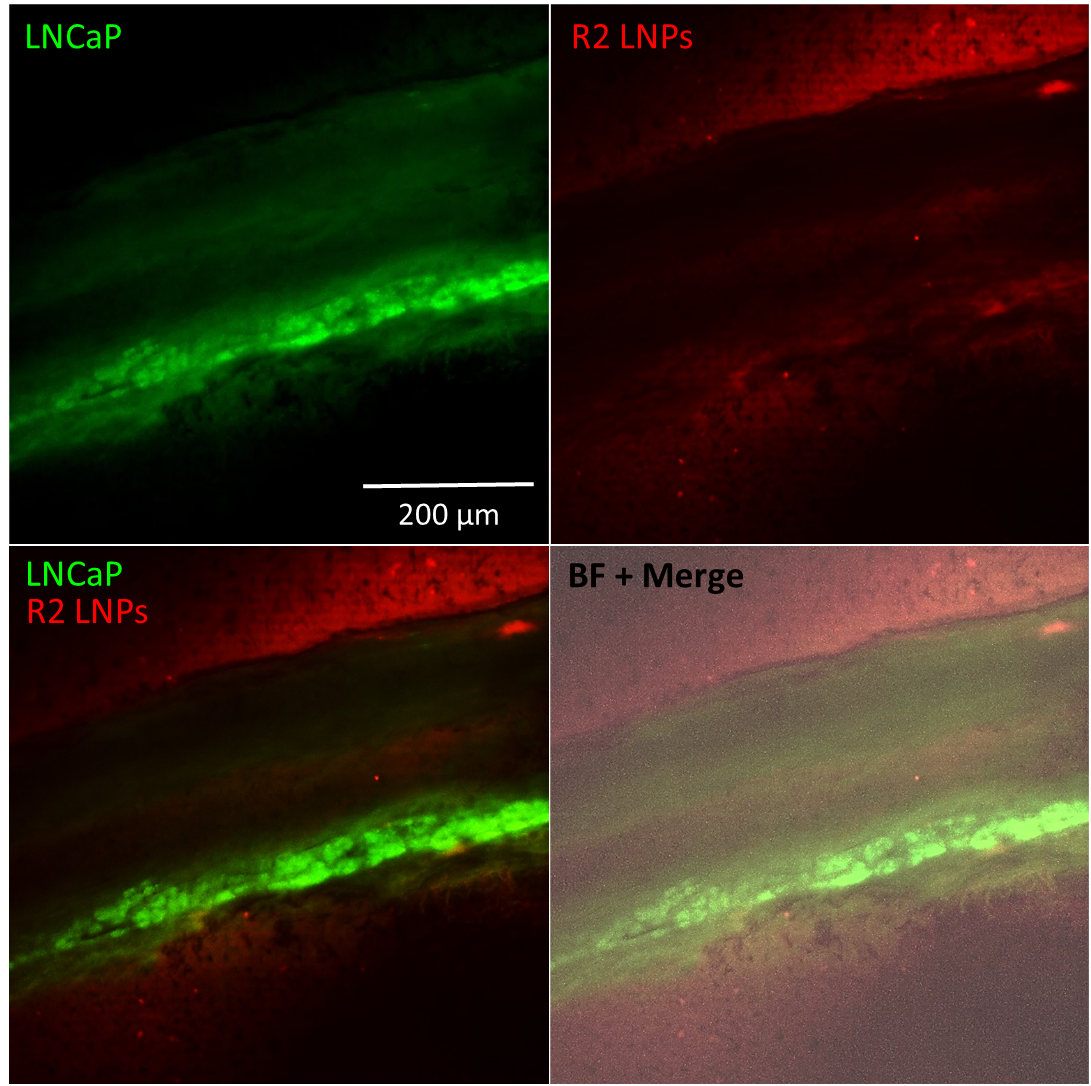
**

**Figure S5 | Anti-PSMA LNPs show targeted mRNA delivery to metastatic PCa cells in a zebrafish xenograft model.** LNCaP (PSMA^+^) cells were engrafted in the PVS at 2 dpf. LNPs were i.v. injected in the Duct of Cuvier at 3 dpf. After 7 h, embryos were fixed with 4% PFA in egg-water. Imaging of the embryos was performed using a Leica SP5 inverted confocal microscope with 20x oil immersion objective and for more detailed images of cells we used a spinning disk microscope at 30x silicon immersion objective, as indicated for each of the image sets. Anti-PSMA LNPs **(A-D,G, I-K)** showed enhanced targeting and functional BFP mRNA transfection in LNCaP cells when compared to R2-LNPs **(E,F,H,L,M)**. For the full embryo images, these are an ensemble of different 20x images recomposed together (G,H). Color legend: Green= LNCaP cells marked with Membright-488™. Red= LNPs containing 0.2% DSPE-Rhodamine. Blue= BFP protein expression. White arrows indicate the accumulation of LNPs (I-M). Injected dose: ~1 mg kg^-1^ mRNA. Injection volume: 2 nL. PVS= perivitelline space; i.v.= intravenous; ZF= zebrafish; dpf= day post fertilization.


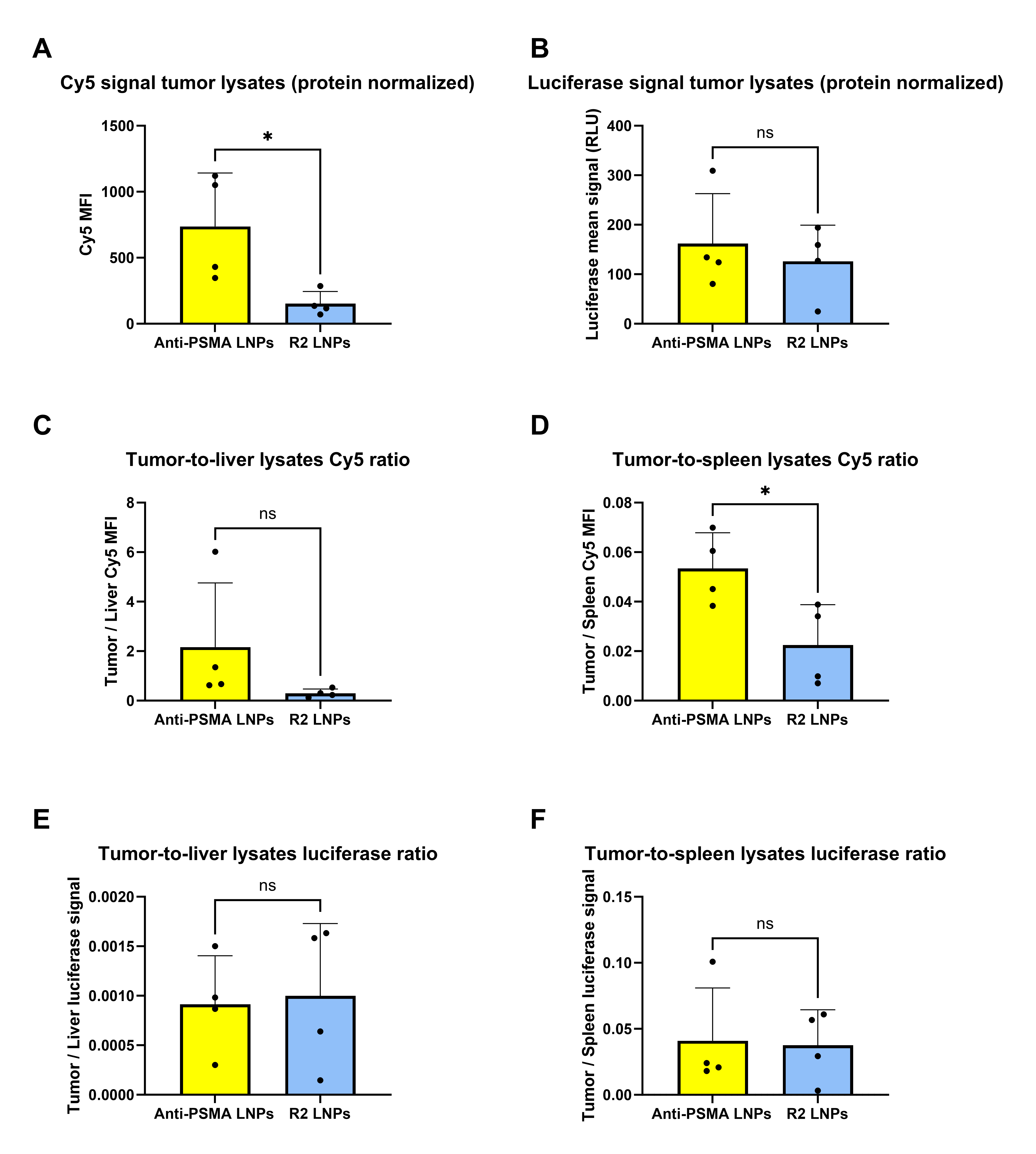


**Figure S6 | Anti-PSMA LNPs exhibited higher tumor-to-liver and tumor-to-spleen accumulation ratios than R2 LNPs, but not mRNA transfection. (A)** Cy5 MFI signal and **(B)** luciferase mean signal in tumor lysates; normalizing on total amount of protein instead of tumor weight. **(C)** Tumor-to-liver and **(D)** Tumor-to-spleen Cy5 MFI ratios per mg of tissue. **(E)** Tumor-to-liver and **(F)** Tumor-to-spleen mean luciferase (RLU) ratios per mg of tissue. For all the graphs statistical differences were assessed with a two-tailed unpaired t-test. Data represent mean ± SD (n=4 animals). *, *p*-value < 0.05; ns: no significant difference.

**Figure S7a |** Anti-PSMA LNPs; B16-F10-PSMA tumor cryosections

**
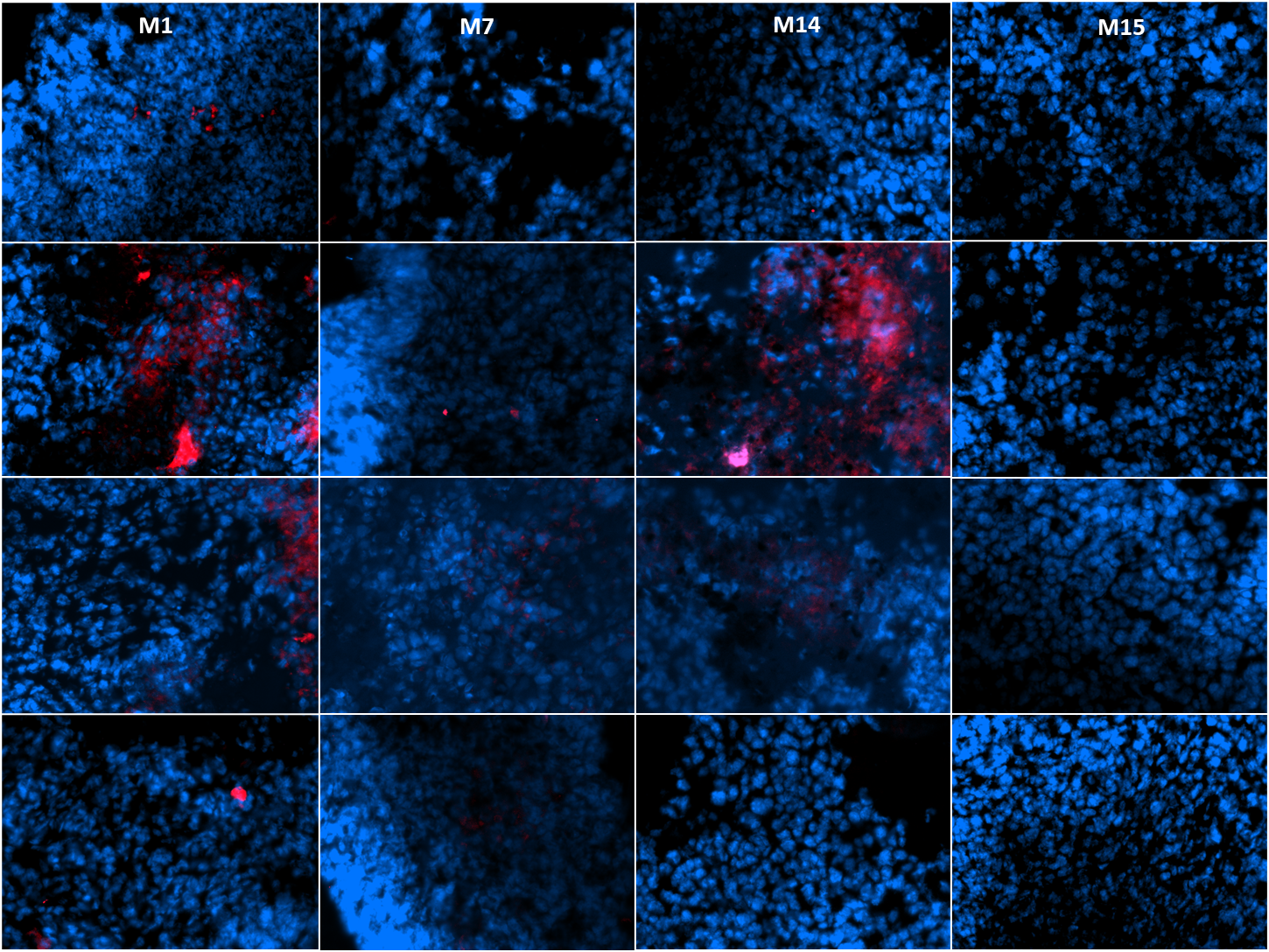
**

**Figure S7b |** R2 LNPs; B16-F10-PSMA tumor cryosections

**
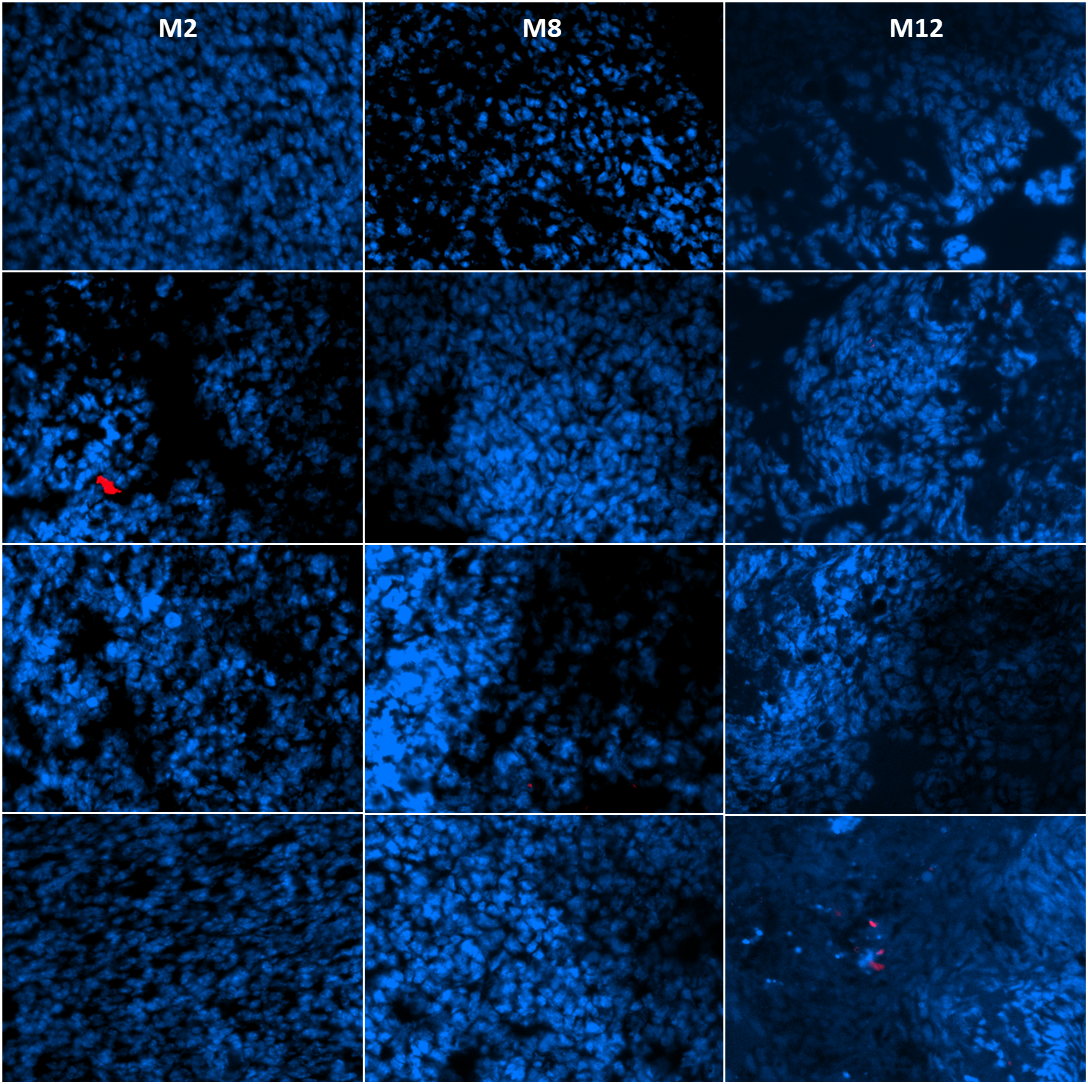
**

**Figure S7c |** PBS; B16-F10-PSMA tumor cryosections

**
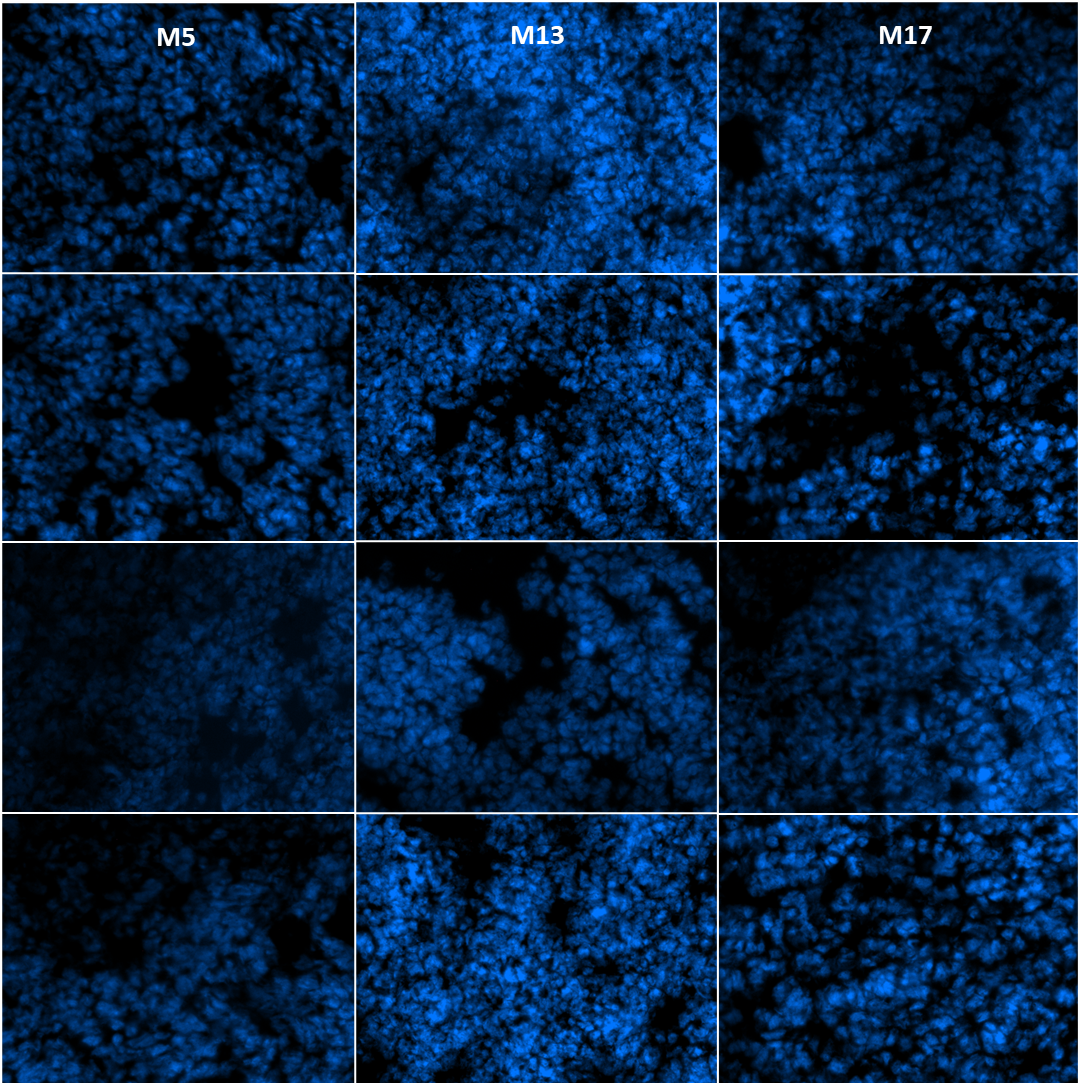
**

**Figure S7d |** Example of quantification of Cy5 signal by subtraction of Cy3 autofluorescence

**
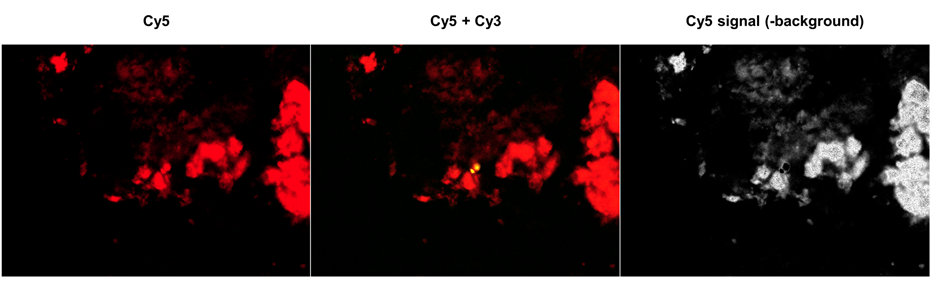
**

**Figure S7 | Cryosections of B16-F10-PSMA tumors revealed enhanced accumulation of anti-PSMA LNPs.** Histological images of B16-F10-PSMA tumors from mice treated with anti-PSMA LNPS **(A)**, R2 LNPs **(B)**, and PBS **(C)** were captured using a Zeiss Axio Observer Z1 Microscope at 20x magnification. Images were focused on random areas of the tumors using the DAPI channel and obtained from different channels upon excitation with corresponding lasers: DAPI (cell nuclei), Cy5 (LNP uptake), and Cy3 (autofluorescence). Consistent laser intensities and visualization settings were applied during image acquisition and processing. **(D)** Example of quantification of Cy5 signal by subtraction of Cy3 autofluorescent signal. The quantification analysis of these images was performed with a custom-made script in ImageJ.


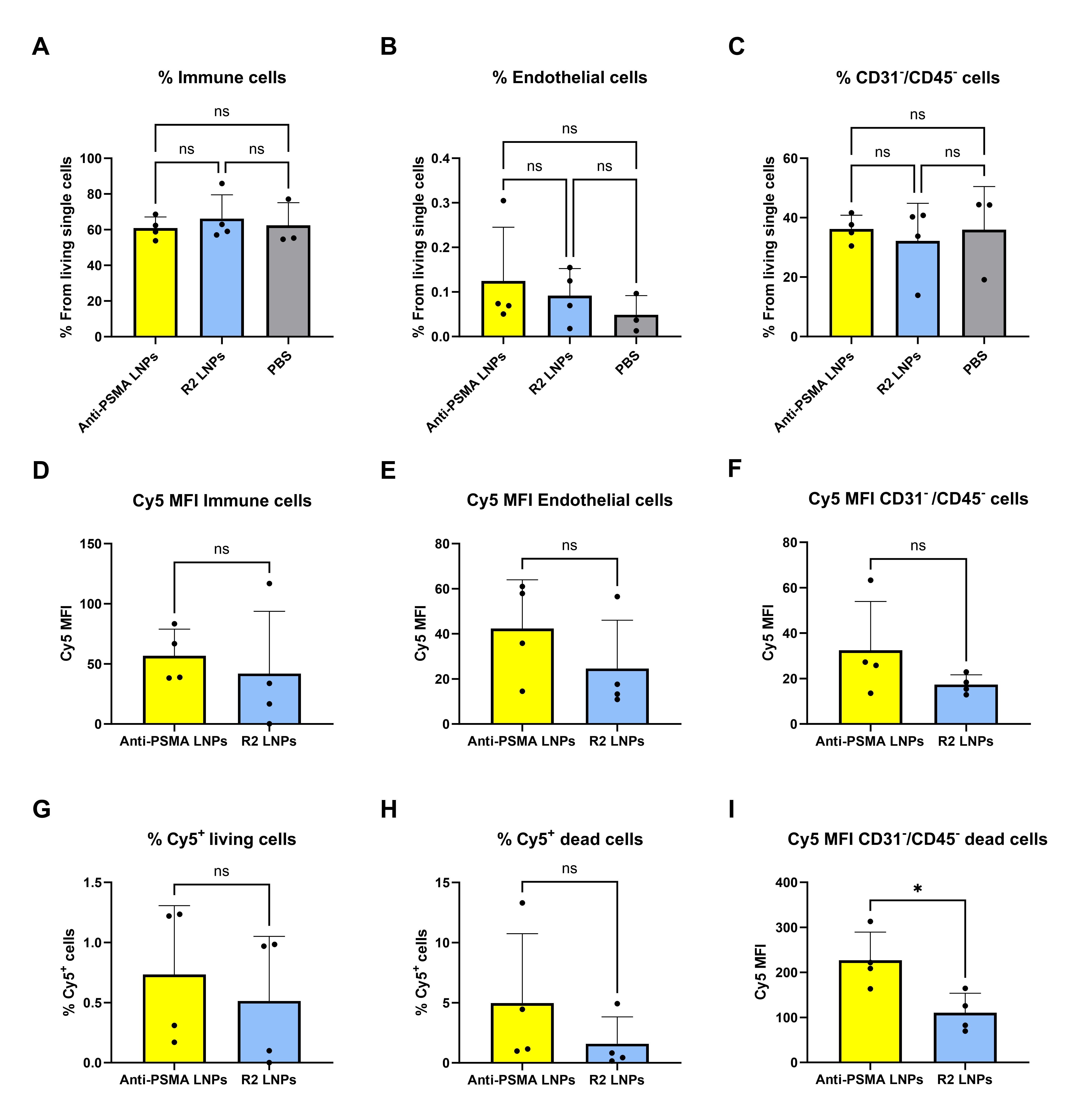


**Figure S8 | Anti-PSMA LNPs show a trend toward increased accumulation in CD31^-^/CD45^-^ cells compared to R2 LNPs, as shown by flow cytometry.** Percentages of **(A)** immune cells (CD31^-^/CD45^+^), **(B)** endothelial cells (CD31^+^/CD45^-^) and **(C)** CD31^-^/CD45^-^ cells out of living single cells. Cy5 MFIs for living **(D)** immune cells (CD31^-^/CD45^+^), **(E)** endothelial cells (CD31^+^/CD45^-^) and **(F)** CD31^-^/CD45^-^ cells. **(G)** Percentage of Cy5^+^ living cells and **(H)** percentage of Cy5^+^ dead cells. **(I)** Cy5 MFI for dead CD31^-^/CD45^-^ cells. For the graphs (A-C) a One-Way ANOVA with Tukey correction for multiple comparisons test was performed. For the graphs (D-I) statistical differences were assessed with a two-tailed unpaired t-test. Data represent mean ± SD (n=4 animals for LNP treated animals; n=3 for PBS). *, *p*-value < 0.05; ns: no significant difference.


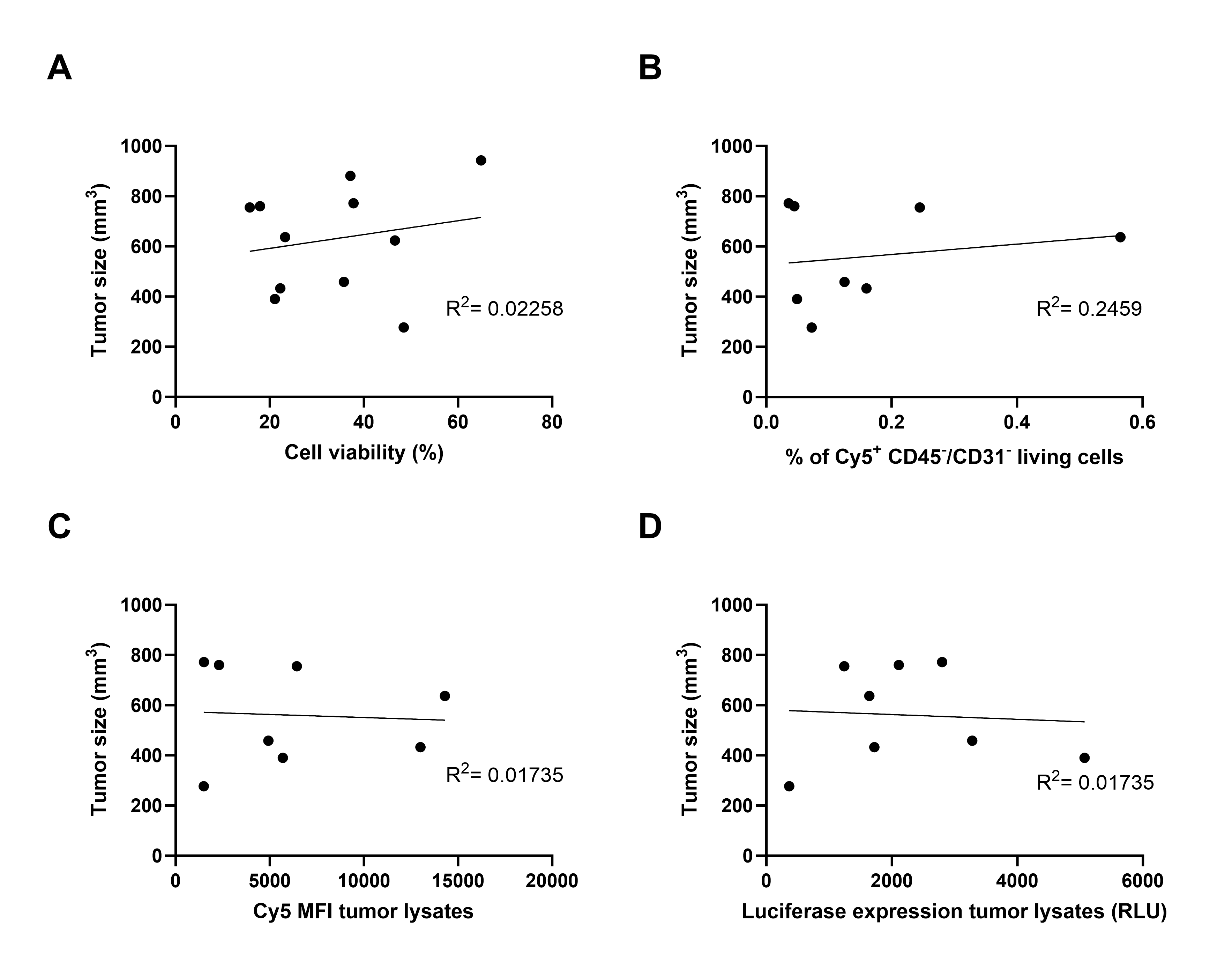


**Figure S9 | Tumor size had no influence in cell viability, LNP uptake or functional mRNA delivery.** Influence of tumor size on **(A)** % of cell viability, as determined by ZA^-^ staining, and on **(B)** the percentage of Cy5^+^ CD45^-^/CD31^-^ living cells, as measured through flow cytometry. Influence of tumor size on **(C)** Cy5 MFI and **(D)** luciferase expression in tumor lysates. Data represent n=11 animals for panel A and n=8 animals for panels B-D (PBS animals excluded). Correlation is analyzed by linear regression (0-1).

**A**


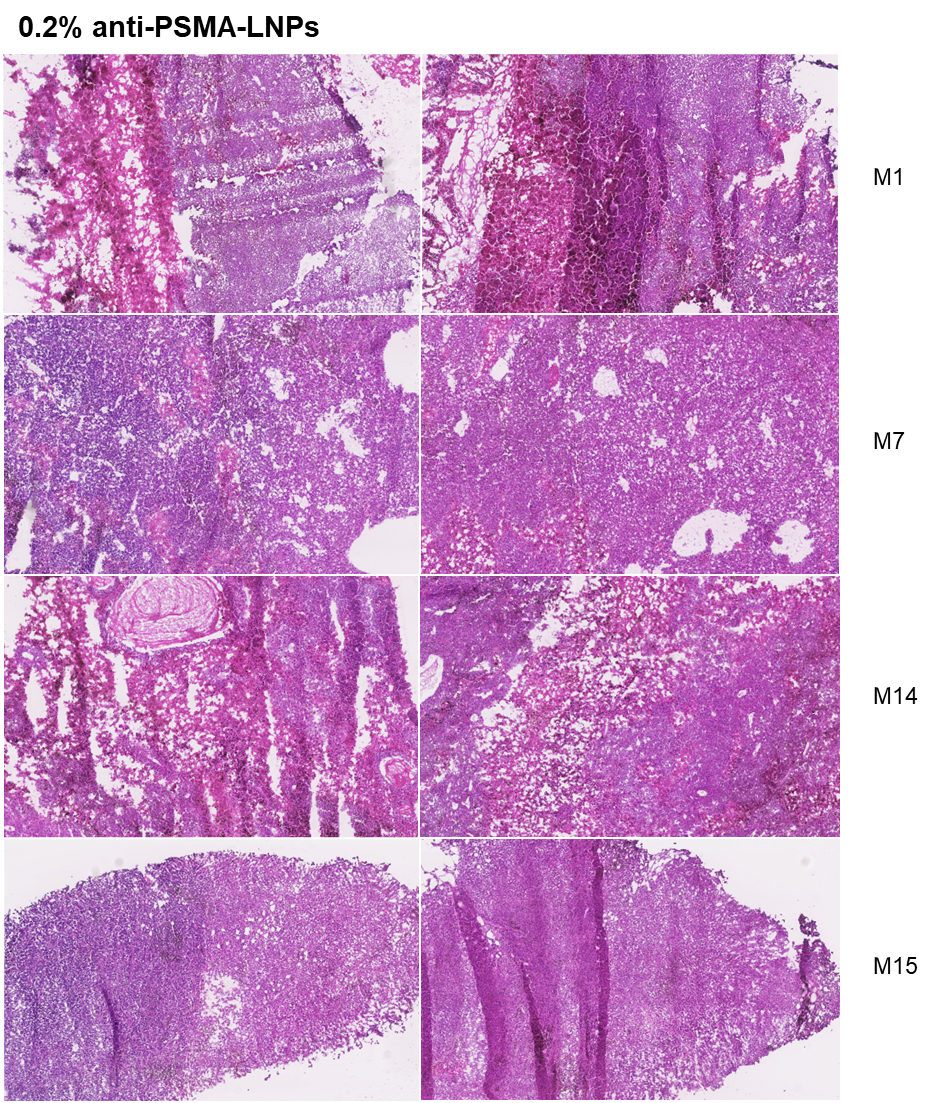


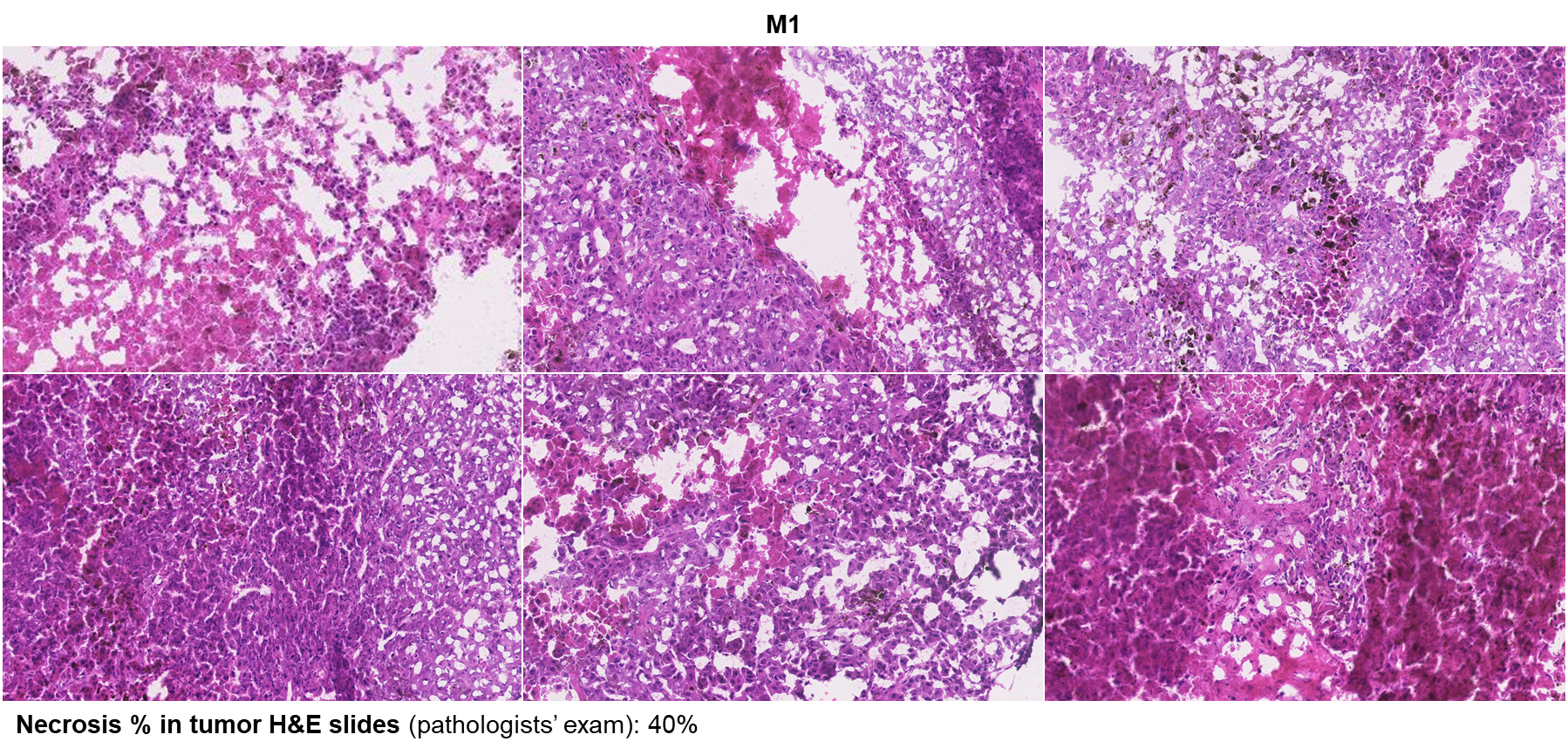


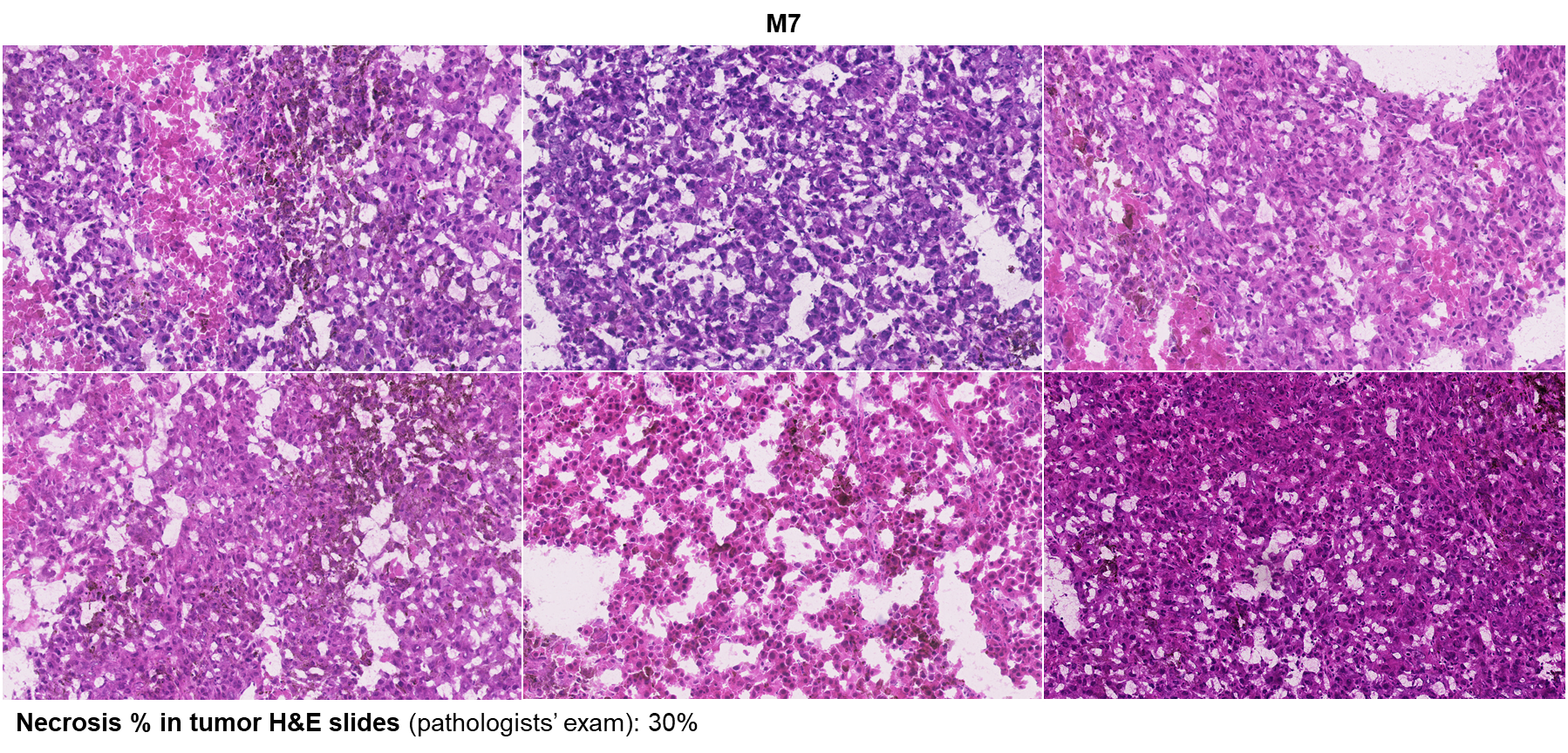

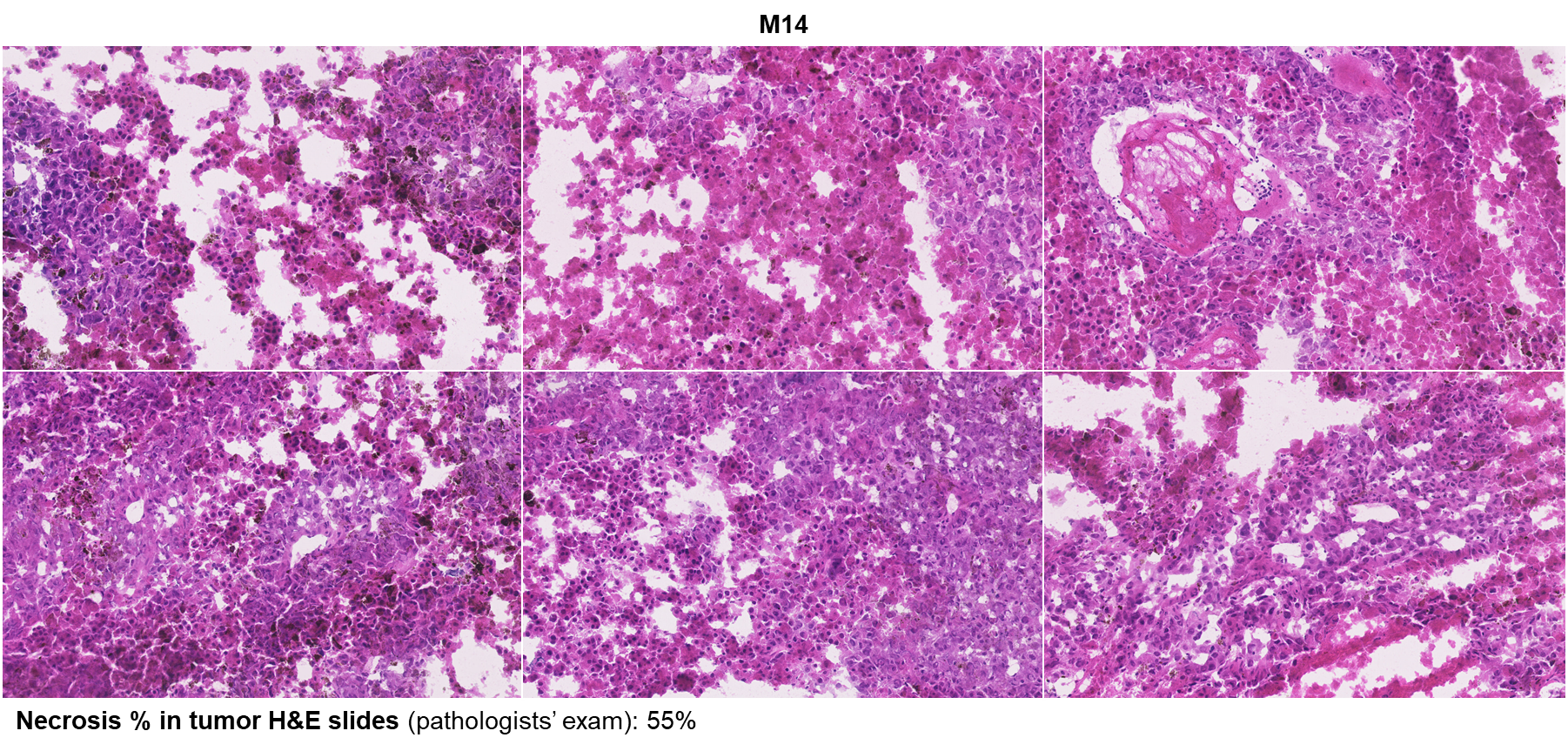


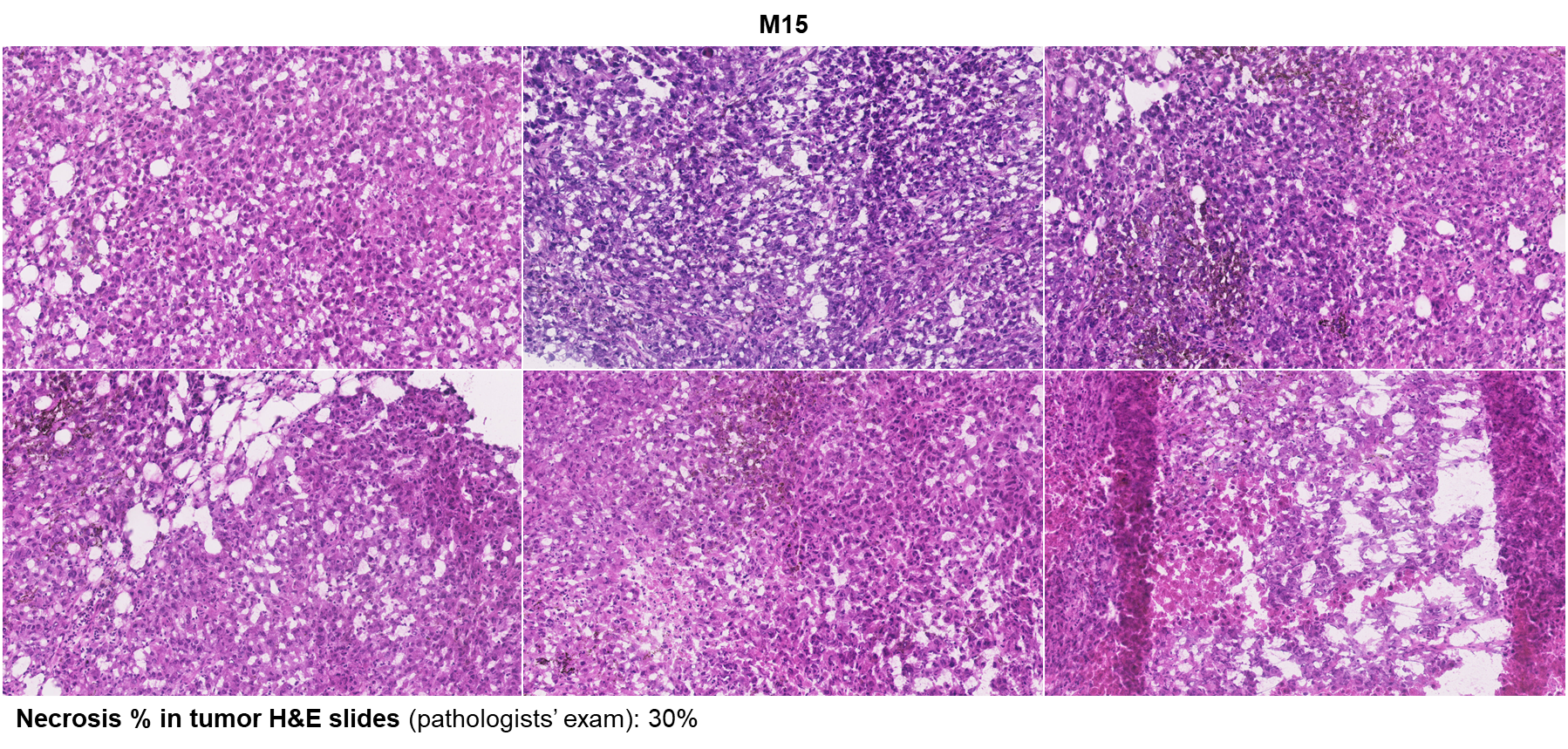


**B**


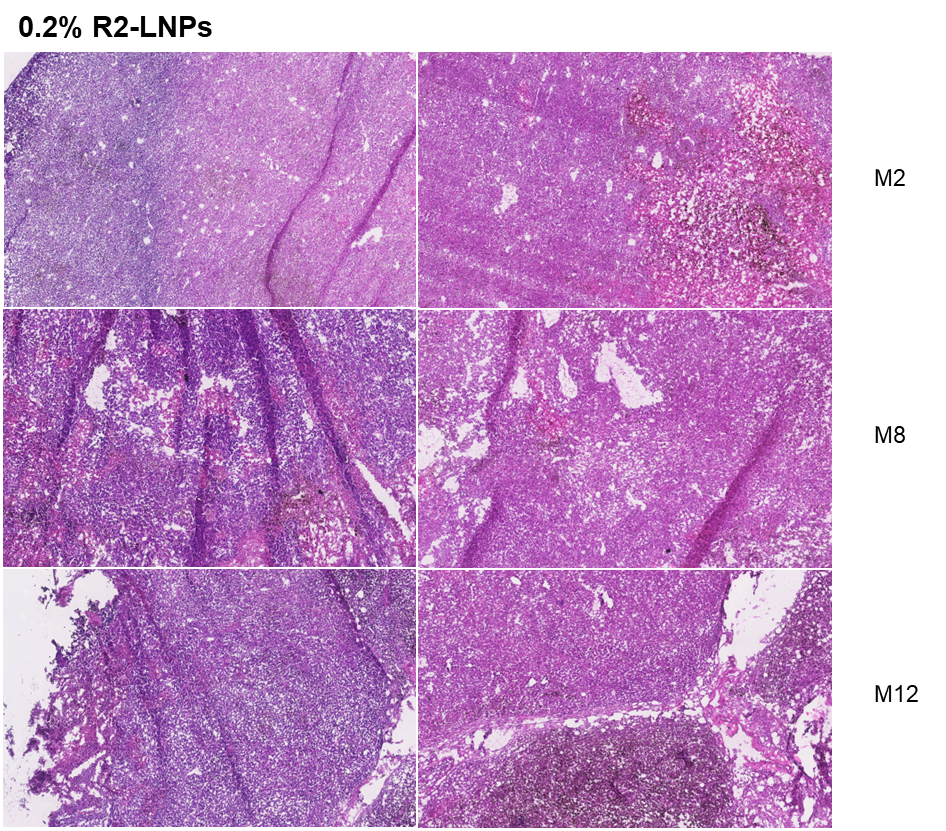


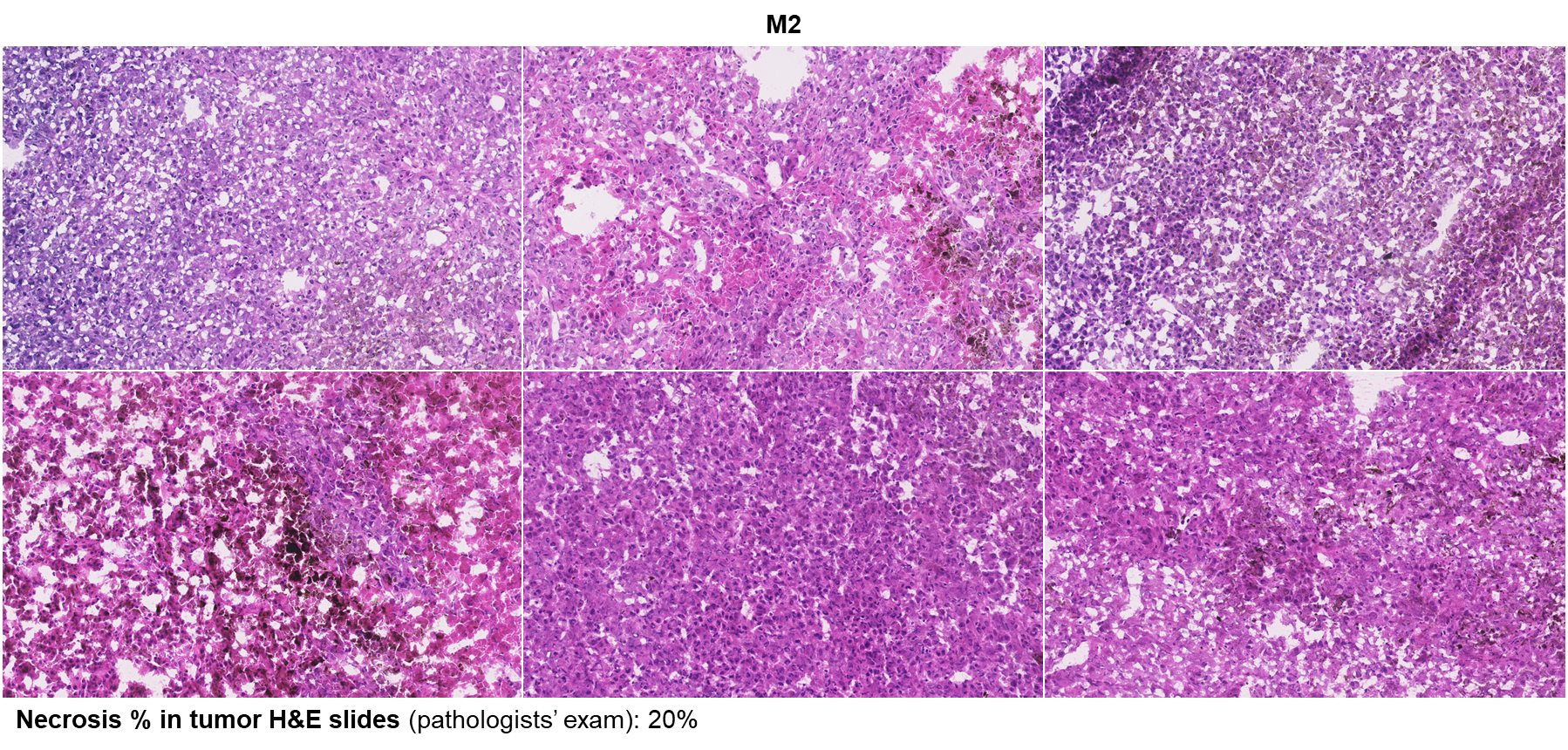


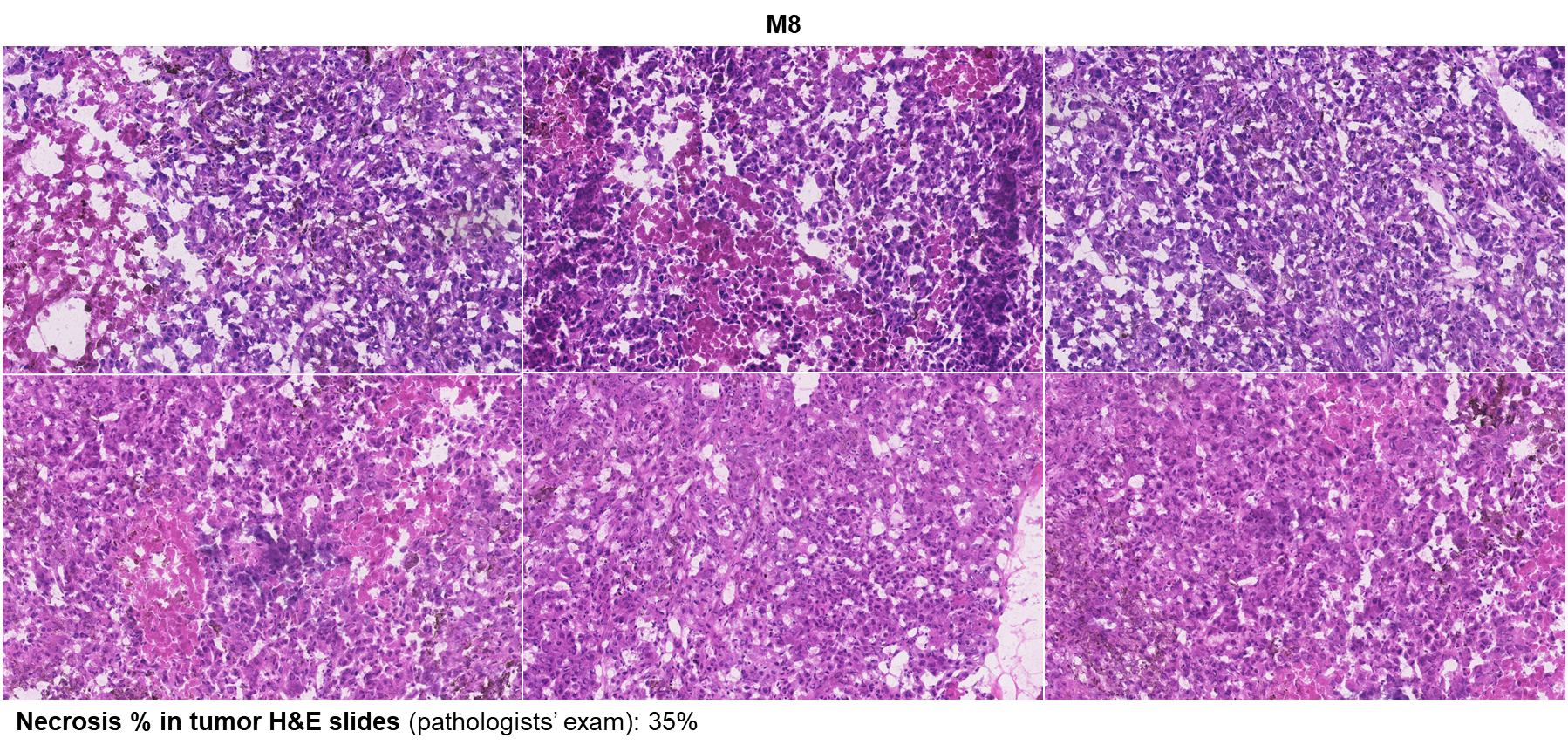


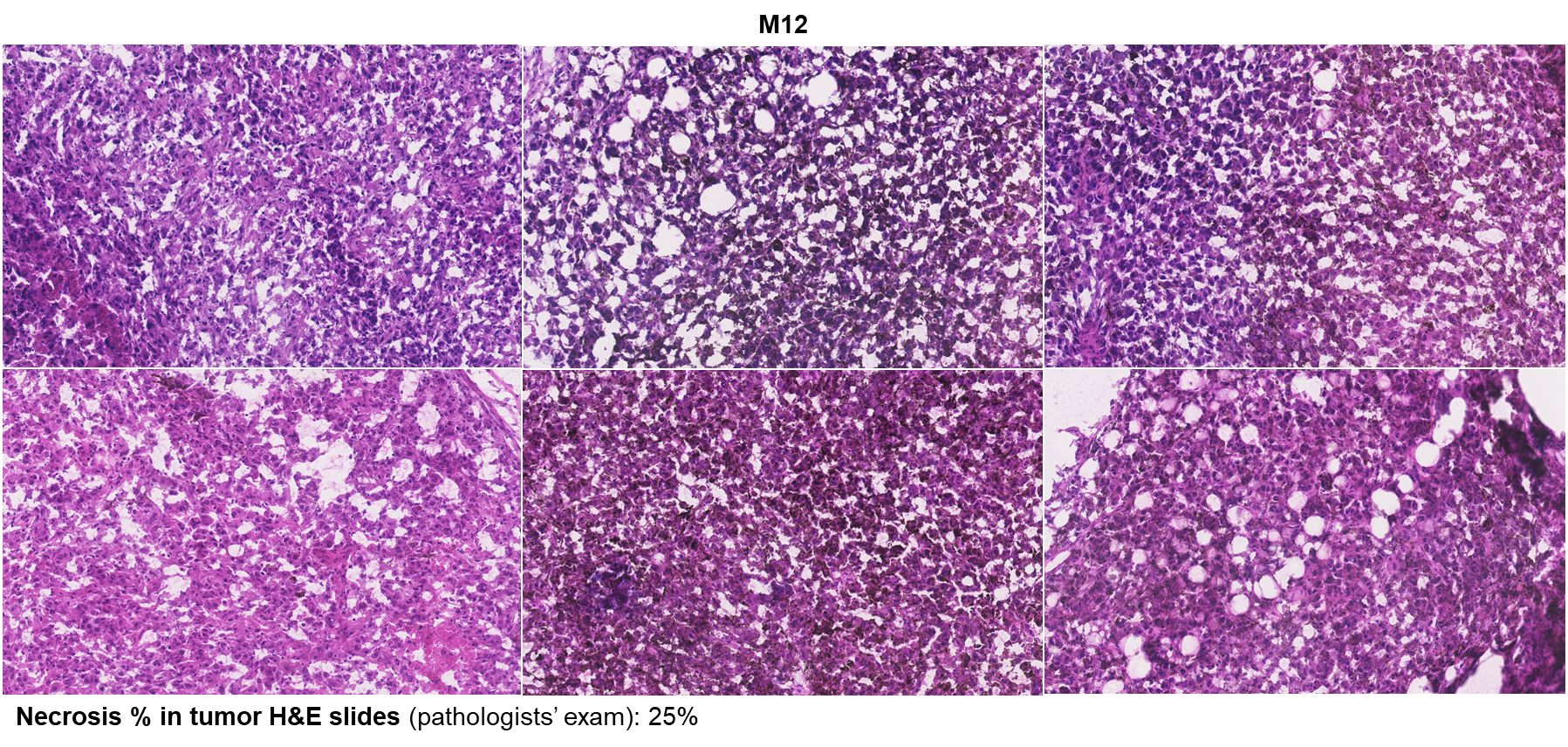


**C**


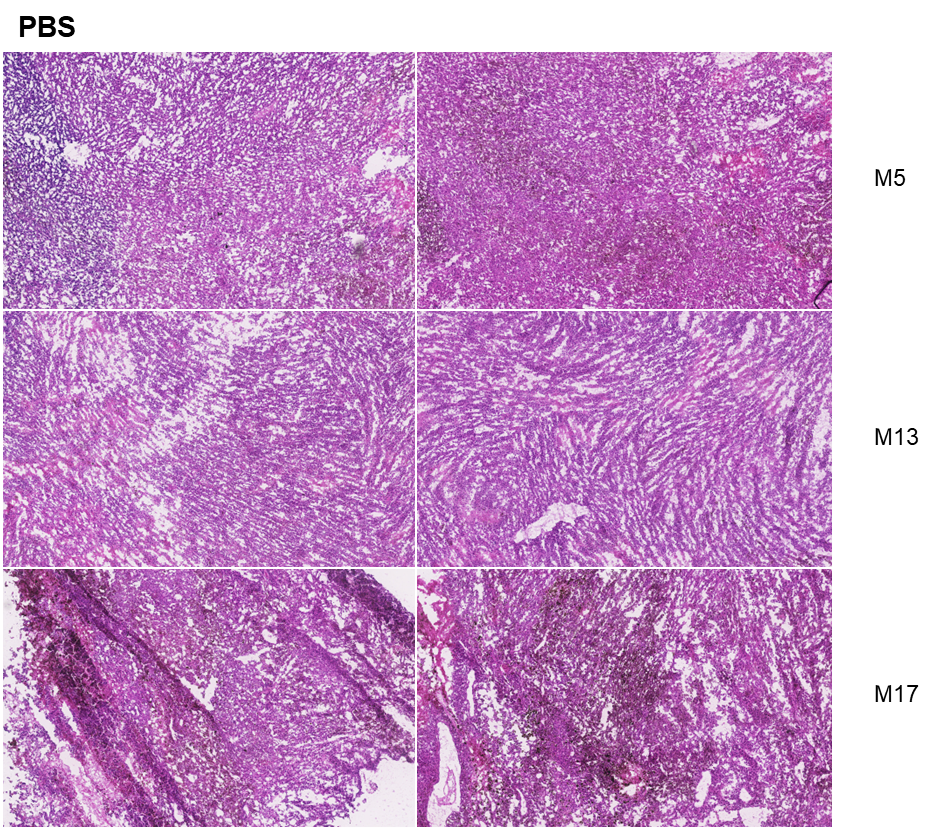


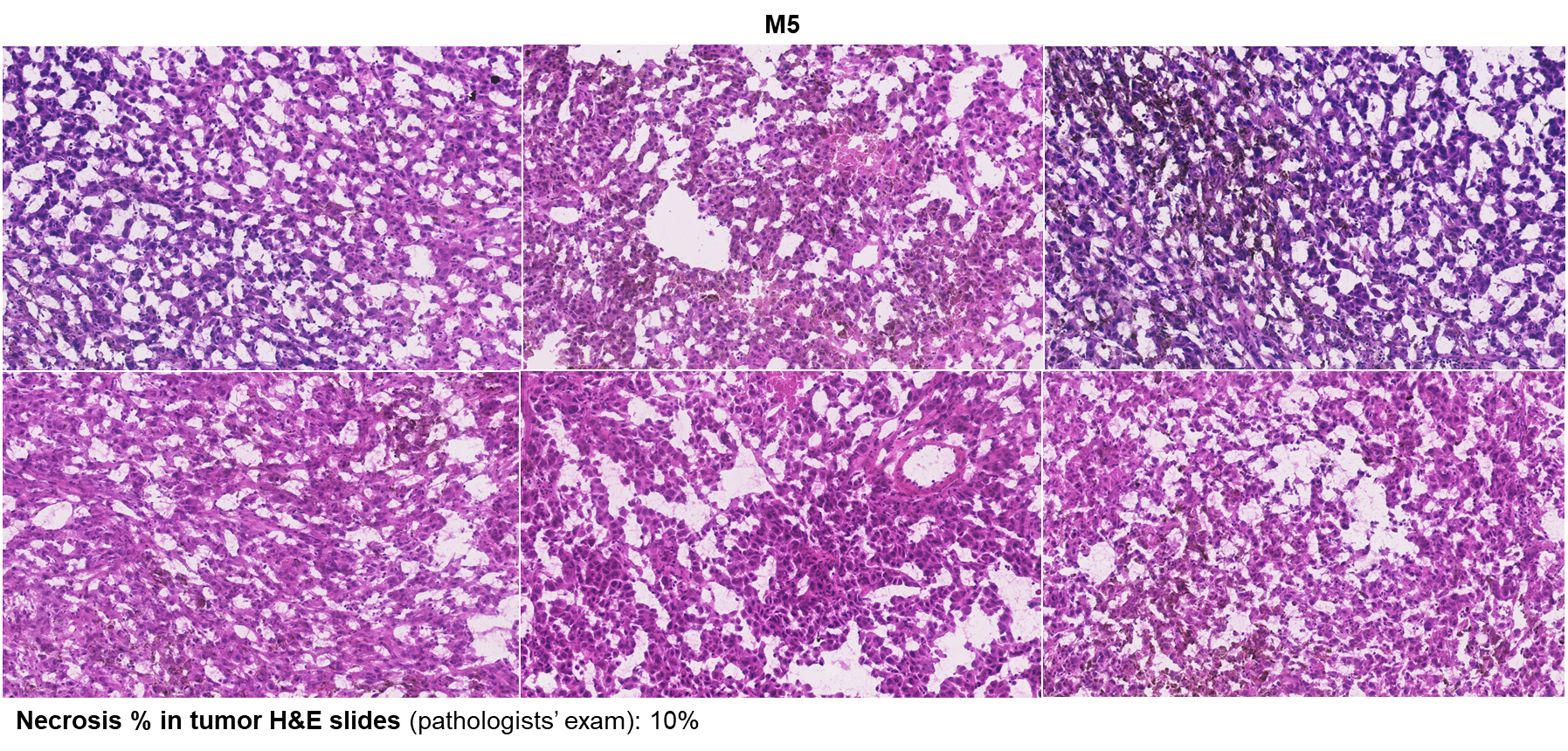


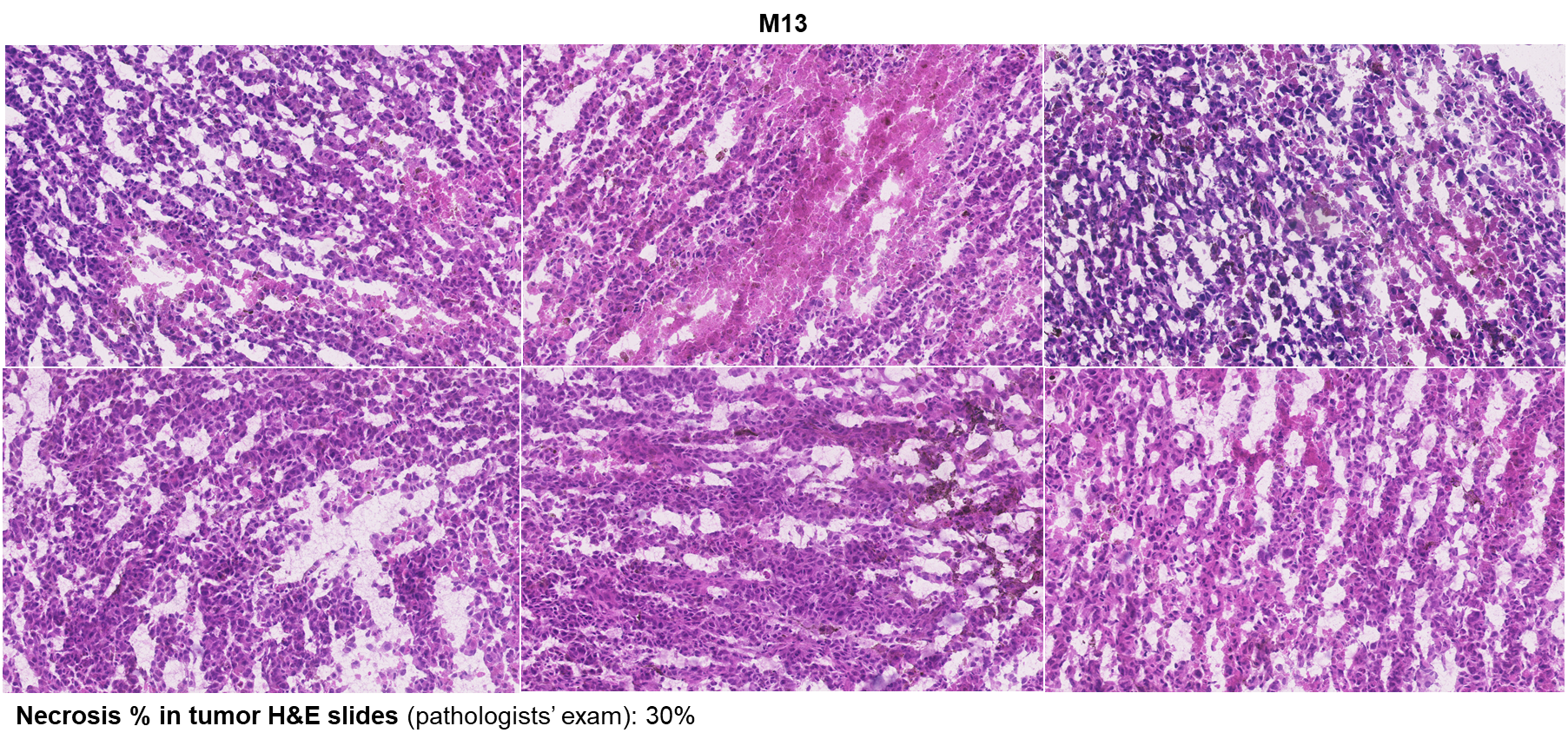


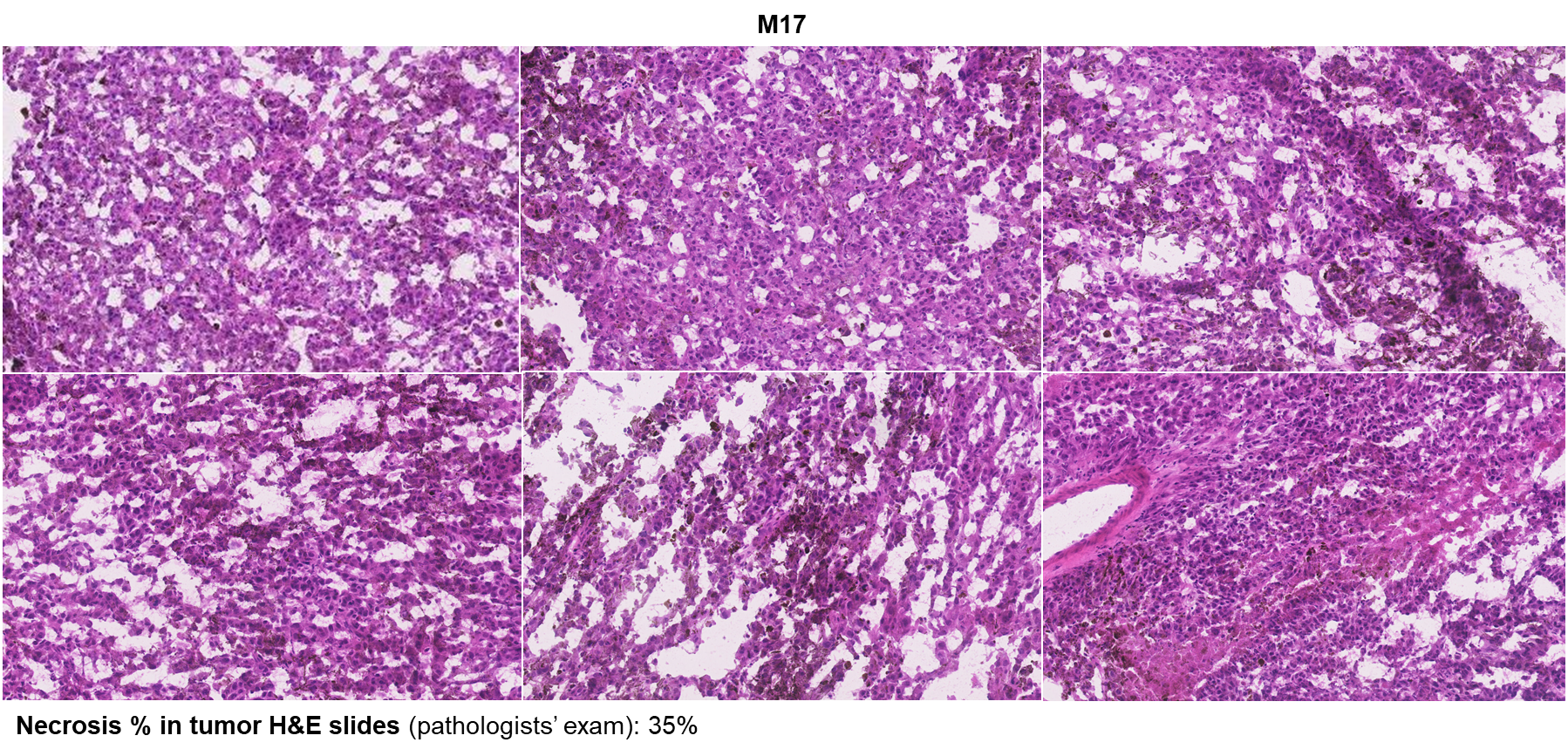


**Figure S10 | H&E analysis of tumor cryosections reveals variable necrosis levels across tumors, as determined by expert pathologists.** Representative H&E-stained images at 5× magnification from two distal cryosections per tumor, followed by three 20× magnification images from each section, are shown for tumors from animals treated with Anti-PSMA-LNPs (**A**), R2-LNPs (**B**), or PBS (**C**). The average percentage of necrosis was estimated by two independent pathologists from UMC Utrecht (TNG, RGO). Data represent n=3-4 animals.


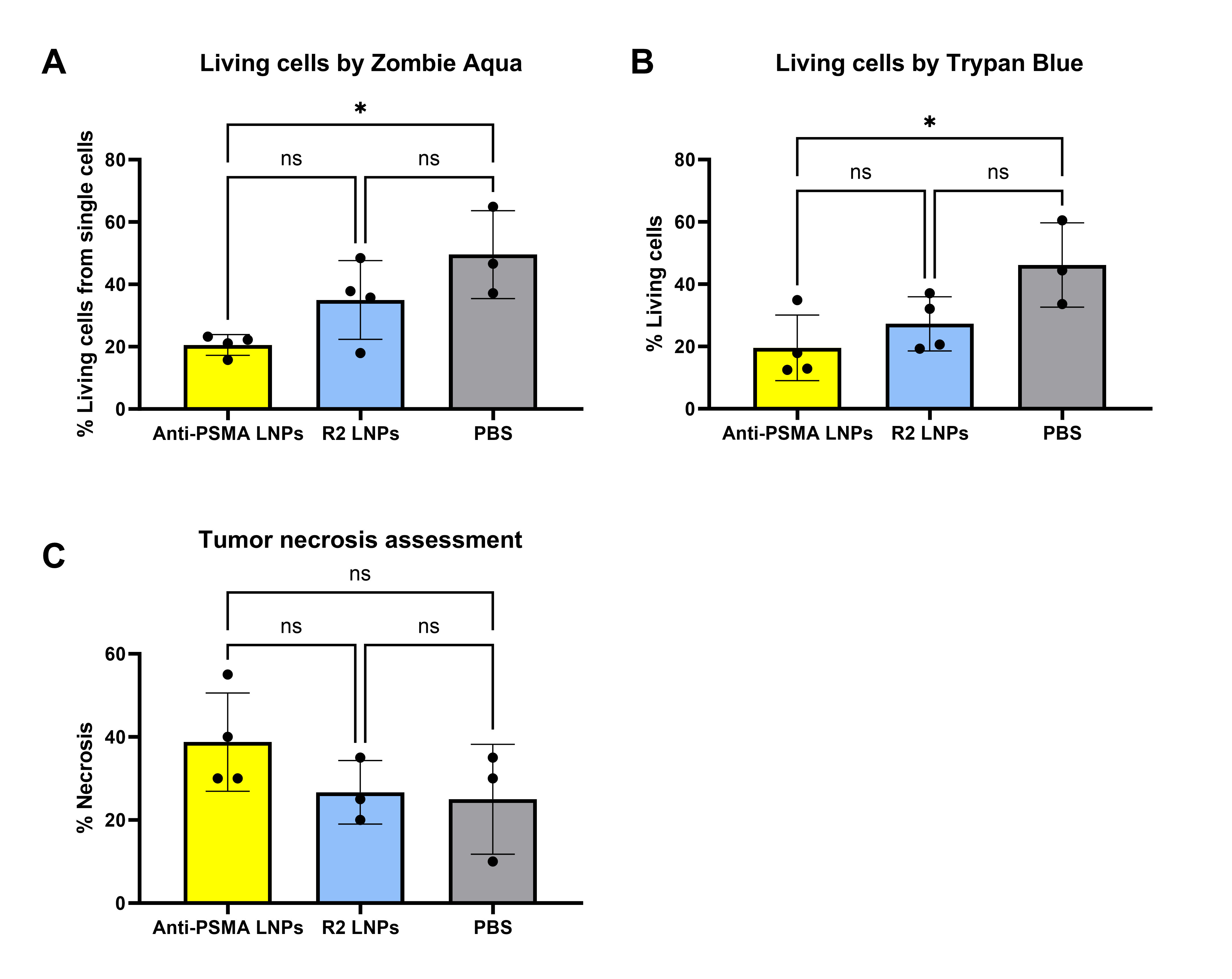


**Figure S11 | Different treatments might have an effect on tumor cell viability.** Cell viability determined by Zombie Aqua^-^ staining **(A)** through flow cytometry or by Trypan blue staining **(B)** in tumor single cell suspensions. **(C)** Tumor necrosis evaluation by expert pathologists from the UMC Utrecht based on two independent H&E cryosections of each tumor. A One-Way ANOVA with Tukey’s multiple comparisons test was performed. Data represent mean ± SD (n=3-4 animals). *, *p*-value < 0.05; ns: no significant difference.


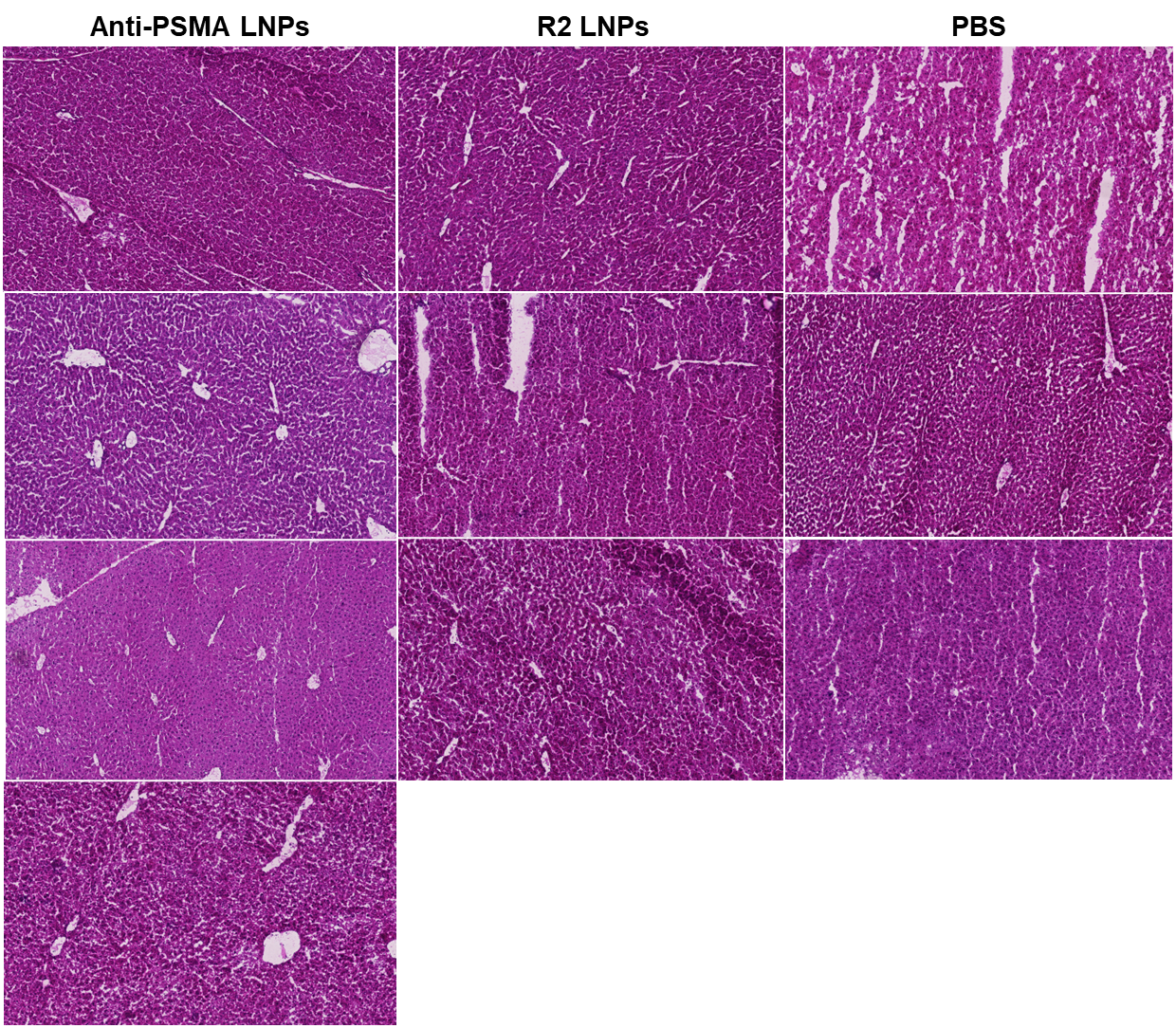


**Figure S12 | H&E analysis of liver cryosections shows no evidence of necrosis across treatment groups, as determined by expert pathologists.** Representative H&E-stained images at 10× magnification from the livers of each treated animal. The average percentage of necrosis was estimated by two independent pathologists from UMC Utrecht (TNG, RGO). Data represent n=3-4 animals.


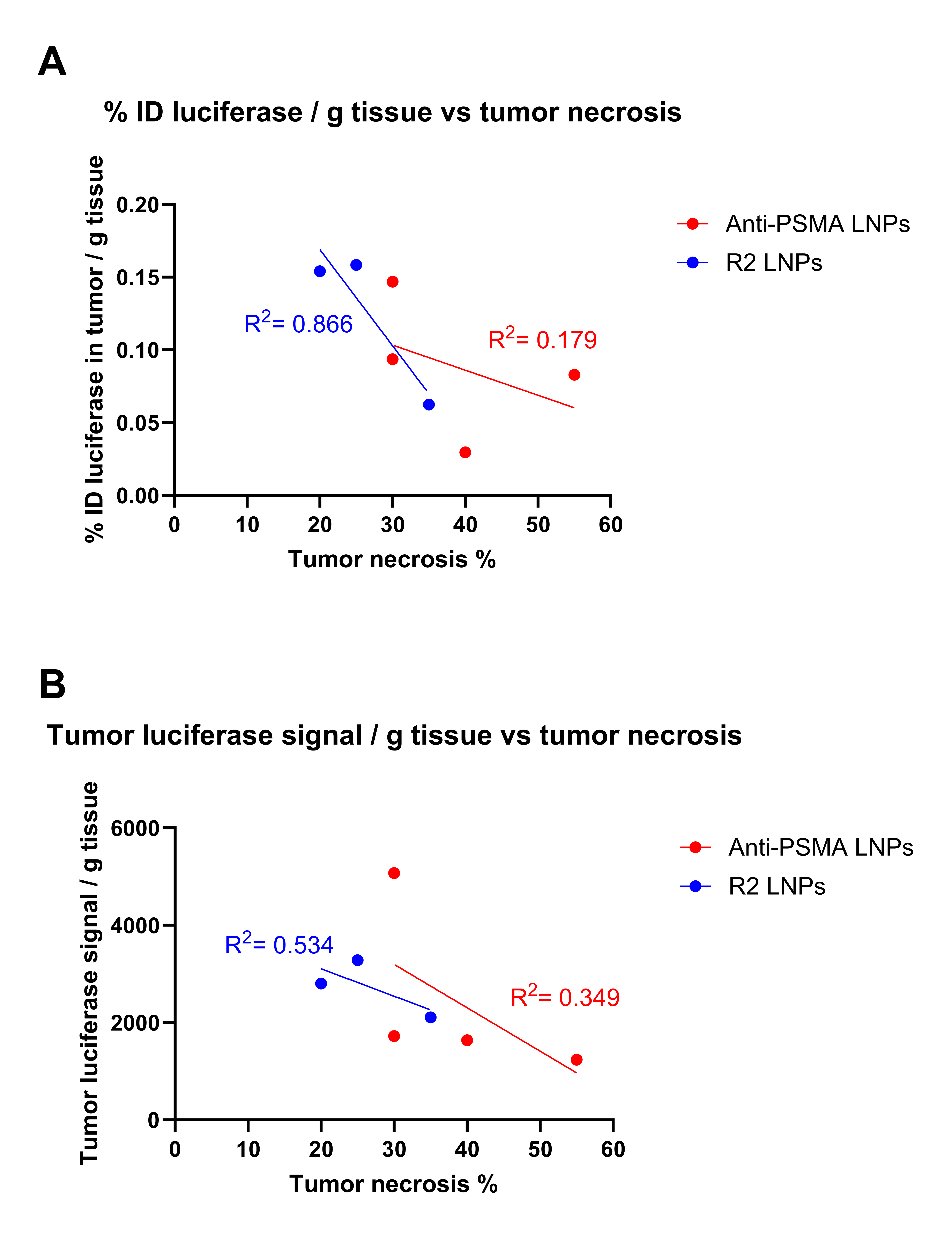


**Figure S13 | Tumor necrosis negatively correlates with LNP-mediated functional mRNA delivery.** (**A**) %ID luciferase / g of tissue in the tumor plotted in relation with the % of tumor necrosis estimation for each animal that received a LNP-treatment. **(B)** Tumor luciferase signal / g of tissue plotted in relation with the % of tumor necrosis estimation for each animal that received a LNP-treatment. R^2^ represents the R squared from a simple linear regression of the X and Y axis for each treatment. Data represent n=3-4 animals (4 animals treated with anti-PSMA-LNPs an 3 animals treated with R2-LNPs).
